# Base-Resistant Ionic Metal-Organic Framework as a Porous Ion-Exchange Sorbent

**DOI:** 10.1016/j.isci.2018.04.004

**Published:** 2018-04-12

**Authors:** Aamod V. Desai, Arkendu Roy, Partha Samanta, Biplab Manna, Sujit K. Ghosh

**Affiliations:** 1 Department of Chemistry, Indian Institute of Science Education and Research (IISER), Dr. Homi Bhabha Road, Pashan, Pune 411 008, India; 2 Centre for Energy Science, IISER Pune, Pune 411 008, India

**Keywords:** Coordination Chemistry, Materials Chemistry, Porous Material

## Abstract

A systematic approach has been employed to obtain a hydrolytically stable cationic metal-organic framework (MOF). The synthesized two-dimensional Ni(II)-centered cationic MOF, having its backbone built from purely neutral N-donor ligand, is found to exhibit uncommon resistance over wide pH range, particularly even under highly alkaline conditions. This report presents a rare case of a porous MOF retaining structural integrity under basic conditions, and an even rarer case of a porous cationic MOF. The features of stability and porosity in this ionic MOF have been harnessed for the function of charge- and size-selective capture of small organic dye through ion-exchange process across a wide pH range.

## Highlights

• Design and synthesis of a rare chemically stable cationic MOF is presented
• The MOF exhibits uncommon resistance under highly alkaline conditions
• The features endow ion exchange of bulky organic pollutants over a wide pH range

## Graphical Abstract

**Figure:**
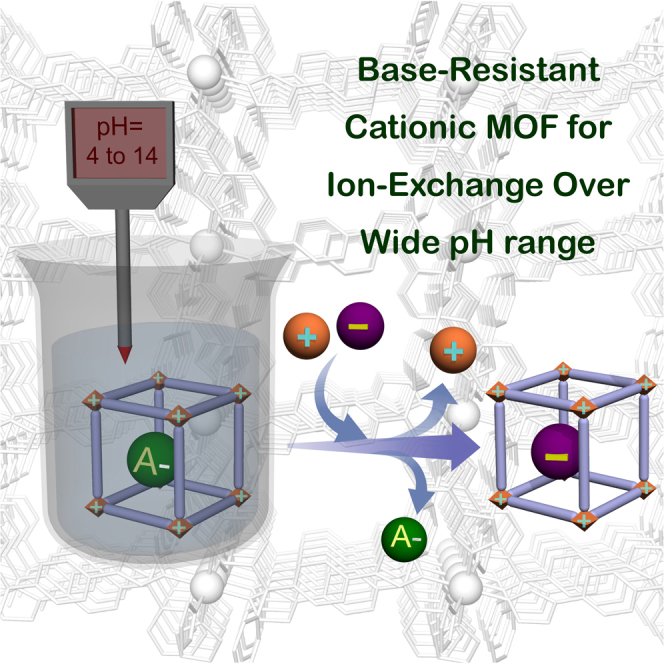

## Introduction

Metal-organic frameworks (MOFs) or porous coordination polymers (PCPs) have rapidly evolved as an important subset of porous materials (Long and Yaghi, 2009, Horike et al., 2009, Furukawa et al., 2013, Zhou and Kitagawa, 2014, Howarth et al., 2017, Maurin et al., 2017). The interest in this domain has expanded in recent years owing to the wide range of applicability exhibited by these materials (Farrusseng et al., 2009, Horcajada et al., 2012, Cui et al., 2012, Sen et al., 2012, Li and Xu, 2013, Ramaswamy et al., 2014, Falcaro et al., 2014, Howarth et al., 2015, Sun et al., 2016, Li et al., 2016a, Aguilera-Sigalat and Bradshaw, 2016, Bao et al., 2016, Lustig et al., 2017). MOFs can be broadly segregated into two classes, based on the charge of the coordination network, viz., neutral and ionic MOFs (i-MOFs); i-MOFs are further classified into cationic and anionic (Karmakar et al., 2016, Li et al., 2016b). MOFs afford significant advancement over congener polymeric materials owing to their crystalline nature, which furnishes precise structure-property correlation. Despite several advantages, there remain a few core issues such as hydrolytic and chemical stability that have stalled the progress of MOFs for real-time applications (Canivet et al., 2014, Burtch et al., 2014, Qadir et al., 2015, Hendon et al., 2017). Although a few benchmark MOF compounds having hydrolytic or chemical stability are known, the majority of them are found to be stable predominantly in acidic pH (Ferey et al., 2005, Park et al., 2006, Cavka et al., 2008, Howarth et al., 2016, Wang et al., 2016a, Wang et al., 2016b, Wang et al., 2016c, Liu et al., 2015, Duan et al., 2017, Bai et al., 2016). The infrequent MOFs exhibiting base resistance are typically neutral frameworks built from azolate-based ligands (Howarth et al., 2016). Development of i-MOFs is seeking greater relevance owing to them being potentially viable alternatives to conventional ion exchangers for various applications (Karmakar et al., 2016, Oliver, 2009, Banerjee et al., 2016, Kumar et al., 2017, Li et al., 2017a, Zhu et al., 2017a, Zhu et al., 2017c, Liu et al., 2017). The challenges of stability assume greater relevance for cationic MOFs, which generally are vulnerable to disintegration in aqueous medium or mild acid/basic conditions. To overcome the limitations of weak hydrolytic or chemical stability, design strategies that can provide robust compounds offering resistance are highly sought after. Surveying the literature, some reviews have chalked out broad principles guiding the design of water and chemically stable, porous MOFs. These include strengthening the metal-ligand bond and shielding this bond from the influence of foreign species (Qadir et al., 2015, Duan et al., 2017). The proper choice of the metal ions or the suitable kind of the organic ligand have also been found to play a crucial role in bestowing stability to a compound. This formal outline has generally been derived from stable benchmark compounds, which in most cases are neutral MOFs. The systematic design and development of stable cationic MOFs from the insights gained in literature reports is extremely uncommon (Karmakar et al., 2016).

With this background we sought to focus on the development of approaches for designing stable cationic MOFs. Typically, cationic MOFs are fabricated from neutral N-donor ligands, which render cationic frameworks and afford the presence of uncoordinated, substitutable anions (Fei et al., 2010, Schoedel et al., 2011, Ma et al., 2012, Manna et al., 2013, Manna et al., 2016, Chen et al., 2013, Hou et al., 2013, Sheng et al., 2017, Zhu et al., 2017b). For affording stability, ligands with higher pKa have found preference (Colombo et al., 2011, Zhang et al., 2012, Pettinari et al., 2016, He et al., 2016, Rieth et al., 2016), and hence ligands with imidazole/triazole-donating units can be more effective as neutral donor ligands (Chen, 2016). Furthermore, the smaller size of five-membered donating moieties can render greater density of the ligands around the metal nodes by feasibility of hexa-coordination, which can shield the metal nodes from the influence of external species. In general, higher dentate ligands are better suited for generating higher dimensional frameworks. In the present discourse, the additional benefit of such linkers is in affording superior kinetic stability (Wang et al., 2016b). Likewise, the appropriate selection of metal center is vital while fabricating stable systems. The choice of the metal node is directed by its ability to bind to the donor groups of the ligands and the resistance to dissociation of the resulting bonds. Among transition metals that bind equally well with N- and O-donor ligands, Ni(II)-based MOFs have been found to offer remarkable hydrolytic stability and, in certain cases, resistance to varying chemical environments (Howarth et al., 2016, Wang et al., 2016b, Colombo et al., 2011, Desai et al., 2016, Lv et al., 2017). In case of cationic MOFs, although the uncoordinated anions are not a direct part of the framework backbone, their choice can be significant in the preparation. From the existing literature, it is observed that organic sulfonates, which are bulky molecules, are known to bind to metal centers typically at higher temperatures only (Fei and Oliver, 2011, Fei et al., 2013, Sergo et al., 2015, Yang and Fei, 2017). In the current context, such compounds can adapt the function of template anions for the creation of voids and can be an integral part of the framework.

Combining the above-mentioned facets, herein we report the synthesis of a two-dimensional (2D) Ni(II)-centered cationic MOF, viz., [{Ni(L)_2_}·(BPSA)·xG]_n_ (L is the ligand; BPSA is 4,4′-biphenyldisulfonic acid; and G is the guest; it is hereafter referred to as IPM-MOF-201, where IPM stands for IISER Pune Materials). The compound is built from a tridentate ligand having terminal imidazole rings and free organosulfonate anions. The compound was found to exhibit extraordinary base resistance, which is uncommonly observed in porous MOFs and even more infrequently observed among i-MOFs. The stability over wide pH range has been tapped for trapping small organic dye molecules across different pH conditions.

## Results

### Synthesis and Characterization of IPM-MOF-201

**Figure 1:**
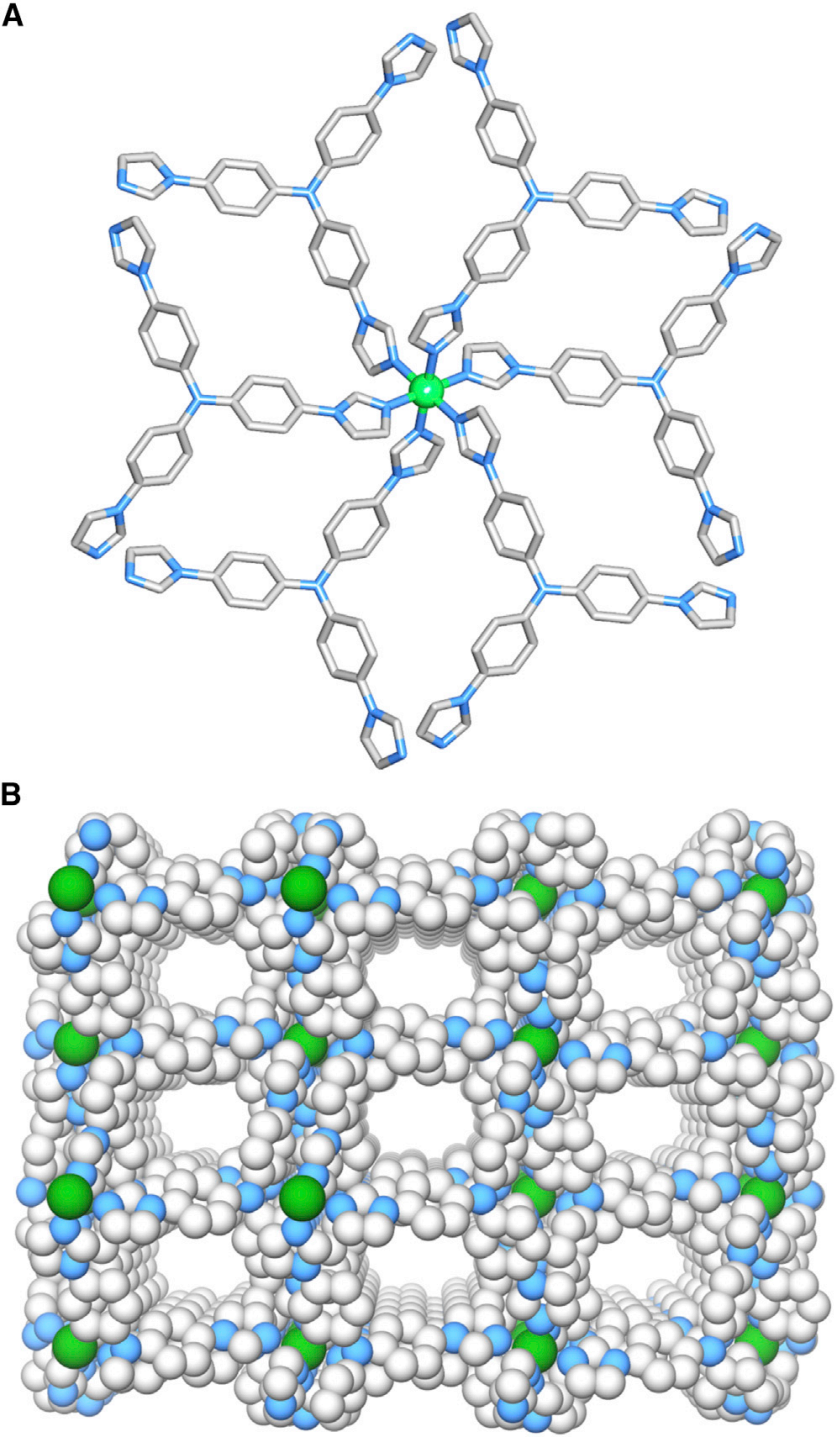
Structural Features and Characterization of IPM-MOF-201 (A) Coordination environment in IPM-MOF-201. (B) Perspective view of packing in IPM-MOF-201 showing porous channels. (Hydrogen atoms and disordered anions have been omitted for clarity. Color code: Ni, green; C, gray, N, blue). See also Figure S1–S19 and Table S2.

Hexagonal-shaped single crystals of compound IPM-MOF-201 were obtained in a solvothermal reaction at 130°C from a mixture of NiSO_4_·*x*H_2_O, BPSA, and ligand (L) (1:1.5:1), in a solvent system of N,N′-dimethylformamide (DMF) and water (2:1) (Scheme S1; see Transparent Methods). Compound IPM-MOF-201 was found to crystallize in *R*-3 space group, as revealed by single-crystal X-ray diffraction (SC-XRD) studies (Table S2). The asymmetric unit is composed of one Ni(II) cation with 1/6 occupancy, one ligand (L) with 1/3 occupancy, and disordered solvent and organic anions (Figure S1). The presence of the organic anion (BPSA) was validated by the ^1^H-nuclear magnetic resonance (NMR) image obtained after digesting the MOF in D_3_PO_4_/D_2_O, followed by neutralization by NaOD (Figure S11; Scheme S2). The metal center is octahedral with coordination from six nitrogen atoms of six independent ligand units (Figure 1A). We determined the topology of the cationic framework to understand the structure further (Blatov, 2004). The analysis revealed that the compound has a (3,6)-connected binodal kgd topology (Figure S8). Notably, this topology is not commonly found in the domain of MOFs (Zheng et al., 2008, Yao et al., 2011, Mitina and Blatov, 2013, Wang et al., 2014a, Liu et al., 2014, Guillerm et al., 2014). The rational choice of building units for obtaining compounds with such topology would be tris-monodentate trigonal ligands. In the context of the present work, the selection of the ligands would be limited to neutral N-donor linkers. The CSD (Cambridge Structural Database) screening approach suggests that MOFs having tridentate five-membered ligands having neutral donating sites with Ni(II) nodes, and crystallization in such packing modes have not been profoundly explored yet, which can lead to realization of chemically stable frameworks. The important feature of this topology is in the creation of intrinsic porosity (Zheng et al., 2008) (Figures 1B and S2–S5), as the resulting 2D sheets are non-planar. In the context of the present study, the non-planarity of the ligands is well suited as it keeps the metal nodes sterically crowded and enclosed within the 2D layers (Figure S6 and S7). The networks crystallizing in kgd topology disfavor interpenetration, which furnishes large voids in the overall packing (Zheng et al., 2008). Based on the PLATON calculations, the solvent-accessible void in IPM-MOF-201 is estimated to be 2754 Å^3^ (49%), considering only the cationic network.

Basic characterization of the compound was carefully performed before all subsequent studies. The purity of the bulk phase was validated using powder X-ray diffraction (PXRD) patterns (Figure S9). The peaks corresponding to the ligand were found to be retained in the Fourier transform infrared (FTIR) spectrum for IPM-MOF-201, along with the peak corresponding to S-O stretching frequency (Figure S10) (Li et al., 2014, Samanta et al., 2017a). Field Emission Scanning Electron Microscope (FESEM) images of IPM-MOF-201 confirmed the hexagonal morphology of the crystallites, and Energy-Dispersive X-ray (EDX) spectra (EDX) spectra supported the purity of the crystallites (Figures S12 and S13). Thermogravimetric analysis (TGA) profile suggested initial loss of guest molecules, water, and DMF (Figure S14).

**Figure 2:**
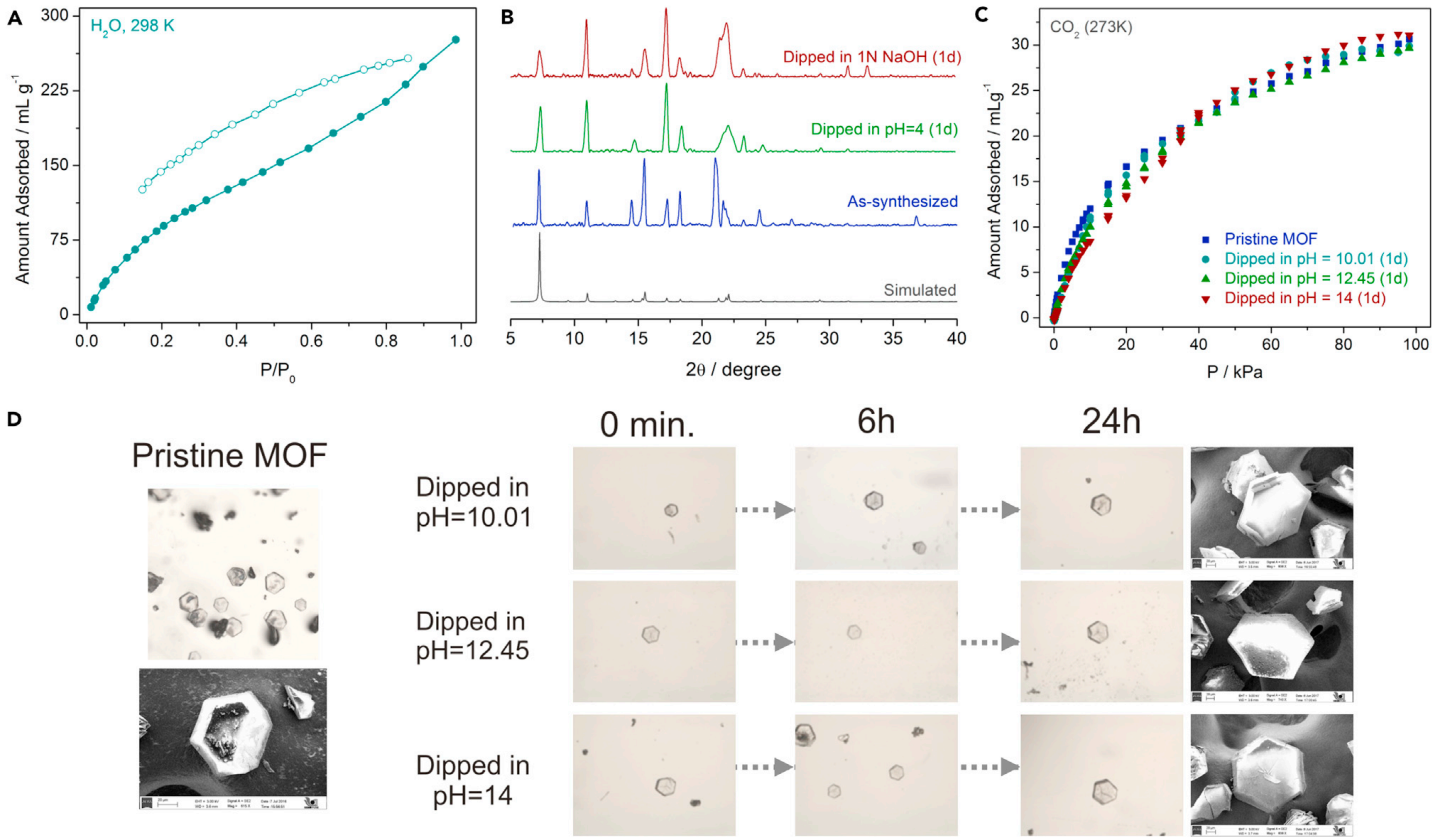
Stability Studies in IPM-MOF-201 (A) Water adsorption isotherm for IPM-MOF-201 at 298 K (closed and open symbols denote adsorption and desorption, respectively). (B) PXRD patterns of pH = 4 (green) and pH = 14 (red) dipped phases, relative to the as-synthesized phase (blue) and simulated pattern (gray). (C) CO_2_ adsorption profiles (273 K) of base-treated phases of IPM-MOF-201. (D) Photographs of different phases recorded by optical microscope and the corresponding FESEM images after 24 hr. See also Figure S20–S38 and Tables S1 and S3.

To substantiate the formation of the compound as the favorable product, synthesis was carried out using different Ni^2+^ salts keeping the molar ratios same. In all the cases, we observed purity of the product from PXRD patterns, FTIR spectra, EDX profiles, and morphological analysis using SEM images (Figures S15–S17). The formation of the compound in different batches validated the favorable formation of IPM-MOF-201. Low-temperature gas adsorption measurements were performed to substantiate the porosity of the compound. We observed almost no uptake for N_2_ (77 K) (Figure S18), whereas there was considerable uptake for CO_2_ (195 K) with strong hysteresis (Figure S18), which suggested strong interactions with the uncoordinated anions. CO_2_ adsorption isotherms were also recorded at 273 K and 298 K, which exhibited uptake of ∼31 mLg^−1^ and ∼20 mLg^−1^, respectively (Figure S19). The water adsorption isotherm (298 K) revealed that the voids present in the compound permitted the entry of water molecules (Figures 2A and S20).

### Stability Studies

To check the hydrolytic stability of the compound over a period, single crystals of IPM-MOF-201 were dipped in water for 30 days, and we found that the morphology of the crystals remained unaffected (Figure S21). The hydrolytic stability was further substantiated by the retention of bulk-phase purity (Figure S22) and CO_2_ adsorption (Figure S31). Enthused from these basic characterizations and the robust nature of the compound in aqueous medium, we then set out to investigate the stability of the compound across varying pH. Initially single crystals of IPM-MOF-201 were dipped in pH solutions of 4, 10, and 12.45, and the retention of the crystallinity was monitored under an optical microscope at different time intervals (Figures 2D and S23).

In all the three cases we found that the crystals remained intact with no significant loss to crystallinity. To extrapolate this observation even further, single crystals were dipped in 1N NaOH (pH = 14) solution and observed at different time intervals, and even in this case the crystals appeared to remain unaffected (Figures 2D and S23). Unit-cell parameters were recorded for the crystals recovered after 1-day treatment at pH = 4 and 1N NaOH. In both the cases the parameters were found to be in close proximity to that of the as-synthesized phase (Table S3), suggesting negligible effect of the pH environment. Upon lowering the pH further, we found dissociation of the framework, which is typically observed for frameworks built from neutral N-donor linkers.

Encouraged by these preliminary observations we then tested this behavior on the bulk scale. Crystals of IPM-MOF-201 were dispersed in separate pH solutions under stirring for 24 hr. The compounds recovered after these treatments were characterized using PXRD, SEM, and gas adsorption measurements. PXRD patterns confirmed the retention of bulk-phase purity (Figures 2B and S24), along with supplementary evidence from FTIR spectra, SEM images, and EDX profiles (Figures 2D and S25–S29). Also, inductively coupledplasma atomic emission spectroscopy (ICP-AES) analysis for the supernatant collected after dipping in pH = 4 and 1N NaOH validated that the compound did not undergo disintegration (Table S1). CO_2_ adsorption measurements at 273 K (Figures 2C and S30) and 298 K (Figure S31) substantiated the resistance of the compound, with almost similar uptakes in all the treated phases, without perturbing the structural integrity (Figure S34). As additional evidence to the above-mentioned observations, we performed NMR studies using deuterated solvents. Crystals of IPM-MOF-201 were dispersed in a solution of NaOD/D_2_O for 1 day (Scheme S3). The solution was centrifuged to separate the filtrate, and CDCl_3_ was added to the residue and rinsed thoroughly. The solution was centrifuged once again and the supernatant collected for recording the NMR spectra (Figure S32). The lack of peaks corresponding to the ligand in the spectra corroborated with the observed base resistance of IPM-MOF-201. In addition, the SEM images of the residue were recorded, and they showed retention of the hexagonal morphology (Figure S33). The residue was subjected to CO_2_ adsorption (298 K), and we found similar uptakes, substantiating the resistance feature (Figure S31). It is noteworthy that the present compound marks an extremely rare example of an MOF exhibiting base resistance and an even rarer case of a cationic MOF retaining integrity under basic conditions. We believe that the structure of the compound affords significant hydrolytic stability owing to the shielding of the metal nodes in the non-planar 2D sheets (Figure S7), as all the coordination sites are occupied by the ligands. It is well understood in the literature that the binding of N-donor linkers to metal nodes can make the pore surface inert and hydrophobic (Zhang et al., 2012). In addition, the utilization of the tridentate ligand with strongly coordinating terminal units affords enhanced stability to the packing (Wang et al., 2016b). Also, Ni(II)-based MOFs are known to be hydrolytically stable, and in certain cases, even stable under extreme chemical conditions (Howarth et al., 2016, Wang et al., 2016b, Colombo et al., 2011, Desai et al., 2016, Lv et al., 2017), providing superior stability to the resulting framework.

As a control experiment to examine the efficacy of the design strategy, we synthesized an isostructural Co(II) MOF keeping all the other reagents and their ratios same. The bulk-phase purity of the thus synthesized MOF, viz., IPM-MOF-201(Co), was validated using PXRD patterns (Figure S35). Although Co-based MOF has been previously reported with ligand (L) (Yao et al., 2011), owing to the bulky anion, the structure obtained is subtly different (Figure S35). Upon primary characterization, the compound was activated in an analogous manner to the Ni(II) compound and then checked for its stability in different pH. Crystals of IPM-MOF-201(Co) were dipped in different pH solutions and monitored under optical microscope at incremental time intervals (Figure S36). Unlike the previous case, the crystals were found to disintegrate with increasing time intervals. This observation was substantiated in the PXRD patterns recorded for the bulk phase after dipping in different conditions (Figure S35). Further control experiments with two previously reported MOFs having similar structures (Yao et al., 2011, Liu et al., 2014) were checked, and in both the cases we observed complete breakdown of the framework under high basic conditions (Figures S37 and S38). These results further support the validity of design strategy in the choice of metal nodes and employment of higher dentate neutral ligands.

### Capture of Anionic Organic Dyes

Along with the critical issue of stability, suitability of MOFs for real-time application in varying chemical environments is a focused aspect of research in this domain. Although only as a consequence of stability, the effective utilization of porosity across wide pH range remains a challenge for this class of materials. Thus to tap the resistance of IPM-MOF-201 across wide pH range and the accessible porosity to foreign species, we sought to investigate the ability of the compound to capture small organic dye molecules. Capture and degradation of dye molecules is an important research problem owing to the serious hazards caused by the release of these compounds in water streams (Wang et al., 2014b). Most of these contaminants have high absorption, which blocks the passage of sunlight to living species in water media (Allen and Koumanova, 2005). Also, on account of high consumption of dissolved oxygen, the aquatic ecosystem is imbalanced. These issues have compounded in recent years owing to the extent and growth of dye-manufacturing industries. It is reckoned that the annual production scale of commercial dyes is close to a million tons (Robinson et al., 2001, Rawat et al., 2016). The allied industries, which include textiles, are estimated to release 10% of the total dyestuffs as industrial wastewaters. Thus given the gravity of this issue, researches have trialed several techniques such as adsorption, coagulation, membrane separation, and photocatalysis (Crini, 2006, Ayati et al., 2014, Sarkka et al., 2015, Singh et al., 2015, Samanta et al., 2017b). Owing to the simplicity, ease of handling, sensitivity, and relative lower cost of operation, adsorption-based methods have commanded greater attention. Thus the development of newer sorbents that can overcome the bottlenecks of existing/conventional adsorbents such as poor selectivity, slow kinetics, and stability are desired. Notably, majority of the studies using i-MOFs as sorbent of dyes have been performed in organic solvents or water at neutral pH (Nickerl et al., 2013, Zhao et al., 2013, Zhao et al., 2017, Chen et al., 2015, Song et al., 2015, Gao et al., 2016, Yu et al., 2016, Jia et al., 2016, Li et al., 2017b, Li et al., 2017c, Nath et al., 2017). Dyes released as industrial wastewaters can have varying pH, and hence the stability and investigation of MOF-based adsorption at varying pH is more pertinent for seeking real-time applicability.

The cationic nature of IPM-MOF-201 and the shape of the pores actuated us to test its trapping ability for anionic dye with linear shape methyl orange (MO). When crystals of IPM-MOF-201 were dipped in an aqueous MO solution (1 mM), the supernatant underwent decoloration over 48 hr, whereas the color of the crystals changed to orange during the same period (Figure S39). PXRD patterns and the SEM images of this ion-exchanged phase suggested retention of structural integrity (Figures S40 and S42). Ultraviolet-visible (UV-vis) spectra were recorded at increasing time intervals to corroborate with the naked-eye observation. As anticipated, the UV-vis spectra of the supernatant showed gradual decrease in intensity with passage of time (Figures S41 and S56). This behavior was retained across different pH of 4.01, 10.01, and 12.45 as well (Figure S44). The PXRD patterns corresponding to these phases demonstrated that the ion exchange at different pH did not affect the structure (Figure S60). To substantiate that the ion-exchange process was charge selective, a cationic dye of similar size, viz., methylene blue (MB), was chosen. The color of the compound did not change upon addition to an aqueous solution of MB and the color of the supernatant did not undergo any change in intensity (Figure S43), as evidenced using UV-vis experiments.

This hypothesis of charge selectivity was further supported when the compound was added to an equimolar mixture of MO and MB. The color of the compound turned orange, and the UV-vis spectra of the supernatant confirmed the selective uptake of only MO (Figures S45–S47). A prototype column experiment was executed wherein this charge-selective separation of dyes could be monitored in a short time (Figure S64). In addition, MB did not entrap even at pH = 10.01, confirming the effect of charge selectivity of the compound (Figure S48). The mixtures of cationic and anionic dyes could be separated over wide pH ranges as well (Figure S65). In addition to charge selectivity, we observed that the ion exchange was size selective. A bulky dye, viz., bromothymol blue, was chosen for this study, and no noticeable change was observed in the time-dependent UV-vis spectra (Figure S53). For real-time applicability, recycling of the adsorbent is highly desired. The cycling efficiency of IPM-MOF-201 for MO dye was checked over three cycles, and the performance was found to be retained (Figure S63).

**Figure 3:**
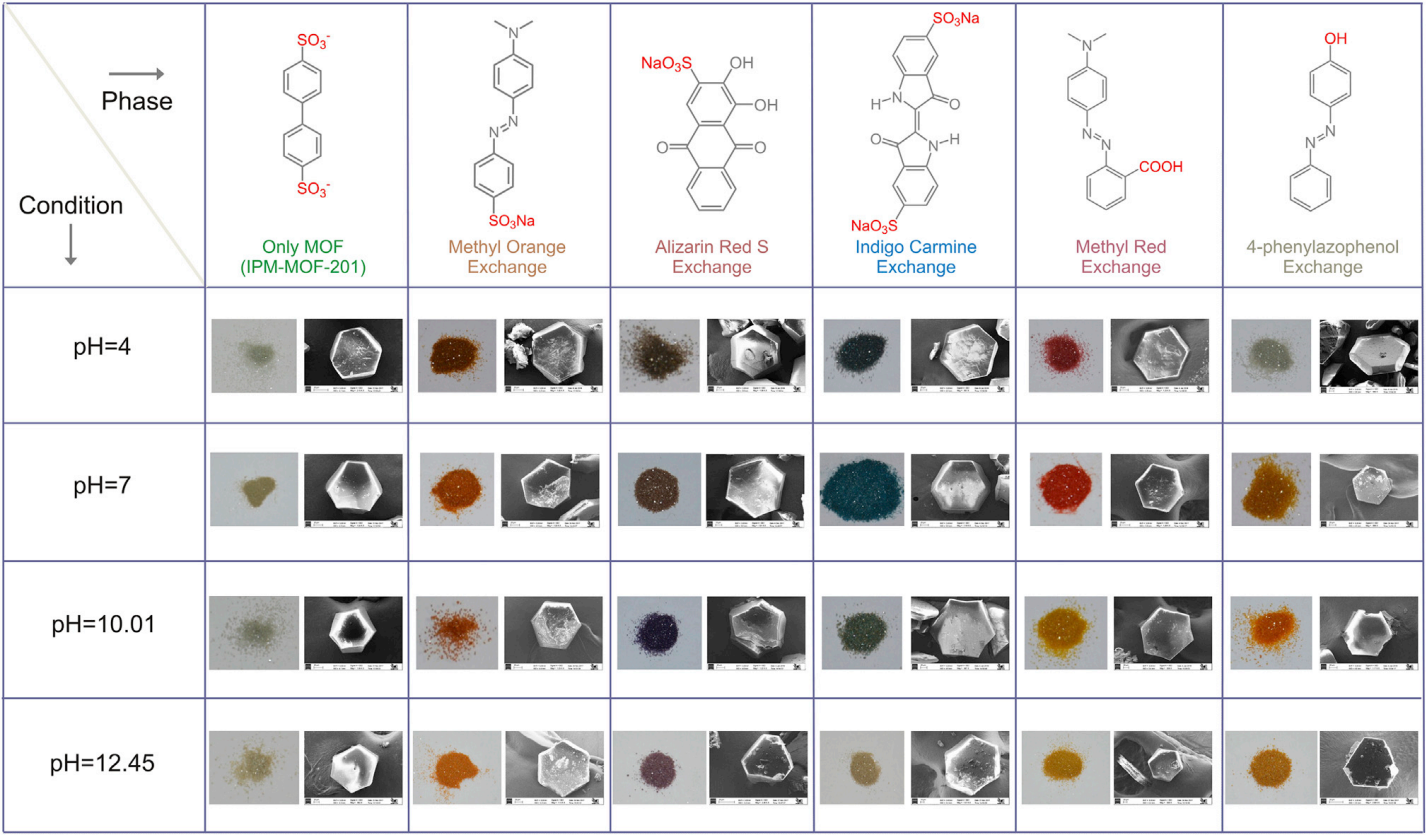
Dye Capture Studies by IPM-MOF-201 Summary showing photographs of all the phases of compound IPM-MOF-201 and the dye-exchange phases under different conditions. The corresponding SEM images of each phase are shown alongside. See also Figure S39–S65.

To extend this behavior even further, we checked the efficacy of IPM-MOF-201 to entrap anionic dyes of similar sizes, viz., indigo carmine (IC) and alizarin red S (ARS). It has been observed in the literature that the dye molecules with dimensions between the minimum and maximum pore windows in porous frameworks can undergo ion exchange (Nickerl et al., 2013, Zhao et al., 2013, Zhao et al., 2017, Chen et al., 2015, Song et al., 2015, Gao et al., 2016, Yu et al., 2016, Jia et al., 2016, Li et al., 2017b, Li et al., 2017c, Nath et al., 2017). Thus linear ions having suitable dimensions can be expected to undergo uptake. In both the cases the color of the compound changed drastically upon addition of the MOF to aqueous solution of the dyes (Figure S49). PXRD patterns for these phases suggested retention of the structural integrity without change to the overall framework (Figure S50). Like the previous case, we recorded time-dependent UV-Vis spectra at different pH, and a similar pattern was observed for both IC and ARS (Figures S51 and S54). Crystals of IPM-MOF-201 were added to an equimolar mixture of two blue dyes (IC and MB). As anticipated, only the peak corresponding to IC underwent decrement without any noticeable change to the peak for MB (Figure S55). As a control experiment, time-dependent UV-vis spectra of blank dye solutions were recorded to validate the ion-exchange process (Figure S52). The anionic dye capture tendency was retained even for dye molecules with carboxylic acid (methyl red [MR]) and phenolic functionalities (4-phenylazophenol [PAP]) (Figures S57 and S59). As observed previously, the structural integrity was not perturbed during the exchange at different pH (Figures S58 and S60). For the dyes studied in this work, the ion-exchange process was also observed under high basic conditions at pH = 14 (Figures S61 and S62). Figure 3 summarizes the naked-eye dye capture process at different pH along with the corresponding morphology of the compound. These results suggest that the feature of differential color of the dyes at different pH is carried unabatedly into the MOF-encapsulated phases as well, making this MOF a potentially useful marker for capture of dyes at specific pH.

## Discussion

In summary, we have developed a hydrolytically stable cationic MOF that exhibits remarkably high resistance across wide pH range. To the best of our knowledge this is an extremely rare example of a porous MOF exhibiting base resistance and an even rarer case of a porous cationic MOF retaining structural integrity even under high basic pH. Control experiments were executed to validate the choice of the building blocks and the design strategy. The stability and the accessible porosity were harnessed for the function of trapping small organic dyes over wide pH range. Notably, hitherto systematic investigation of dye capture over wide pH range has not been investigated systematically in the literature of MOFs. We believe that the results obtained from this work will contribute significantly to the development of design principles for i-MOFs that offer stability over varying chemical environments.

## Methods

All methods can be found in the accompanying Transparent Methods supplemental file.

## Data and Software Availability

The accession number for the IPM-MOF-201 reported in this paper is [CCDC]: [1561299].

## Supplemental Information

Document S1. Transparent Methods, Figures S1–S65, Tables S1–S3, and Schemes S1–S3

Data S1. Crystallographic Data for IPM-MOF-201
