# Supplementary material for: Base-Resistant Ionic Metal-Organic Framework as a Porous Ion-Exchange Sorbent
Source: iScience. 2018 Apr 12;3:21–30. doi: 10.1016/j.isci.2018.04.004 (PMC6137287; doi:10.1016/j.isci.2018.04.004)
Supplement: Document S1. Transparent Methods, Figures S1–S65, Tables S1–S3, and Schemes S1–S3 [file mmc1.pdf]

ISCI, Volume *3*

Supplemental Information

Base-Resistant Ionic Metal-Organic

Framework as a Porous Ion-Exchange Sorbent

Aamod V. Desai, Arkendu Roy, Partha Samanta, Biplab Manna, and Sujit K. Ghosh

FIGURES

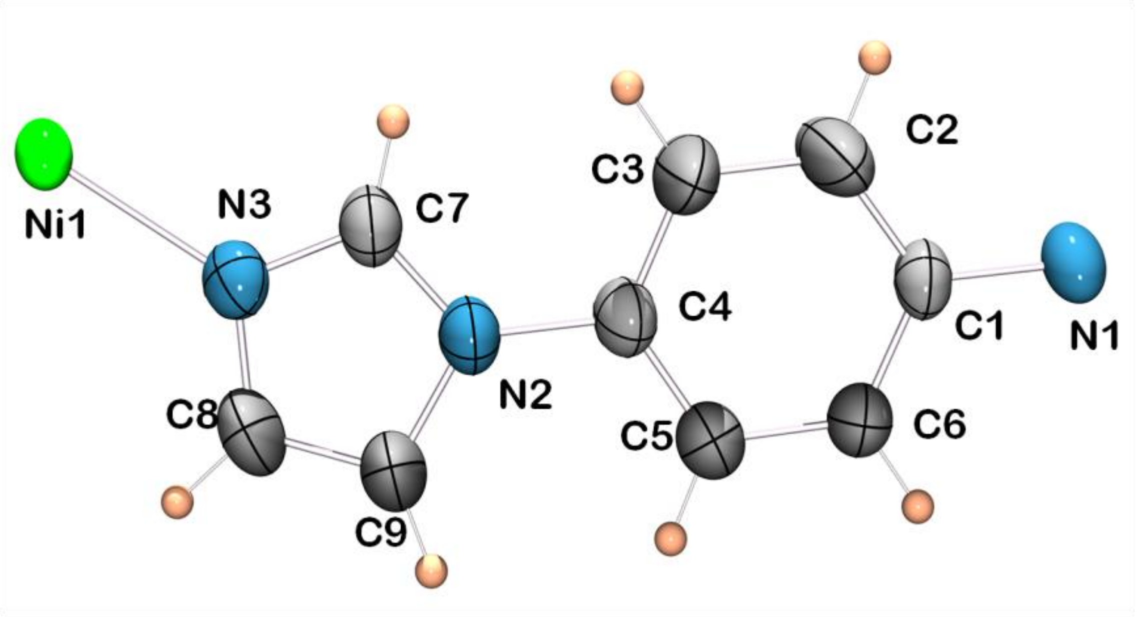

Figure S1: Structural Features of IPM-MOF-201, related to Figure 1.

ORTEP diagram of asymmetric unit of **IPM-MOF-201** in thermal ellipsoids.

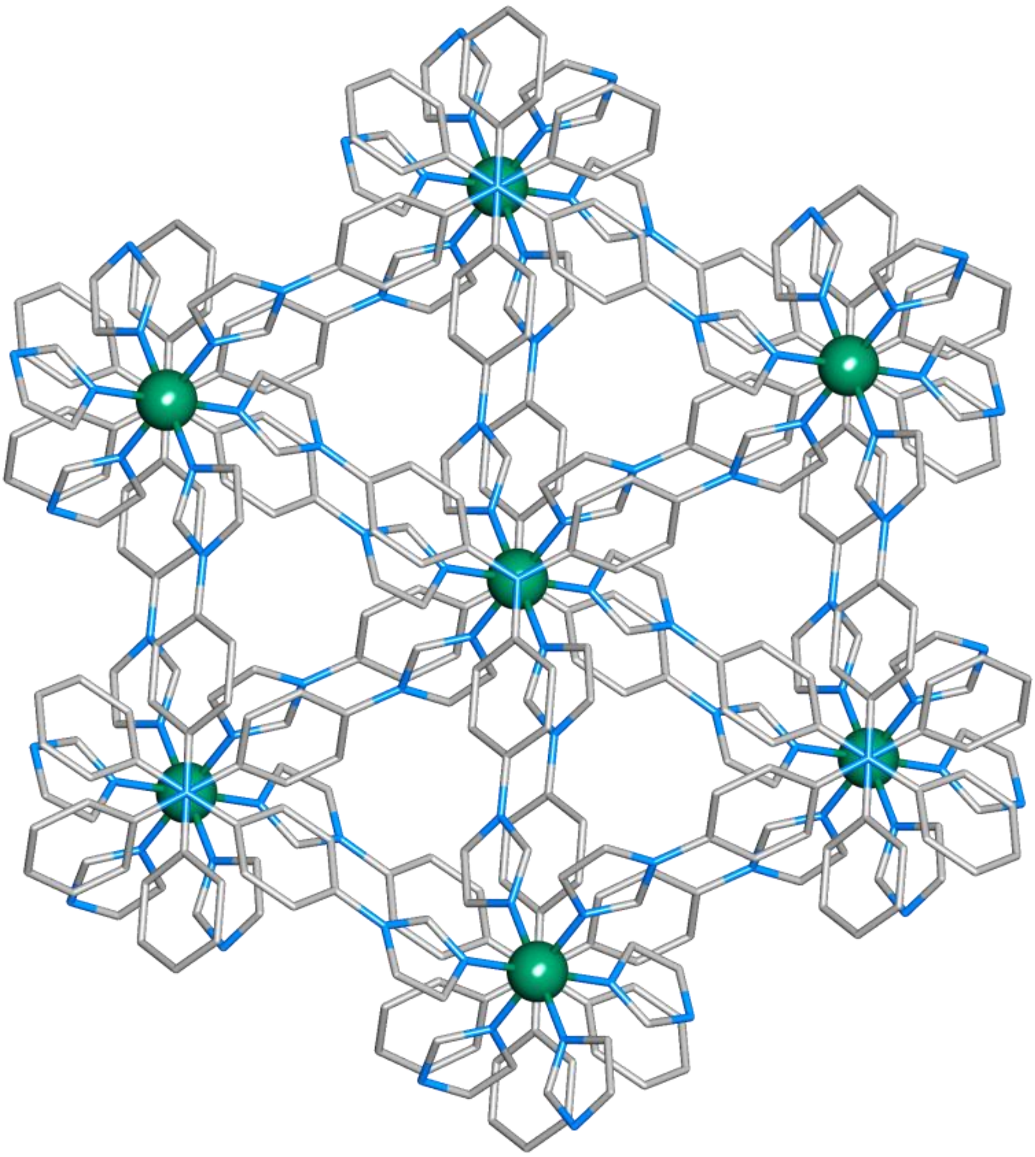

Figure S2: Structural Features of IPM-MOF-201, related to Figure 1.

Figure showing packing of **IPM-MOF-201** along crystallographic *c*-axis. (Hydrogen atoms and disordered anions have been omitted for clarity, colour: Gray - C, Blue - N, Green - Ni).

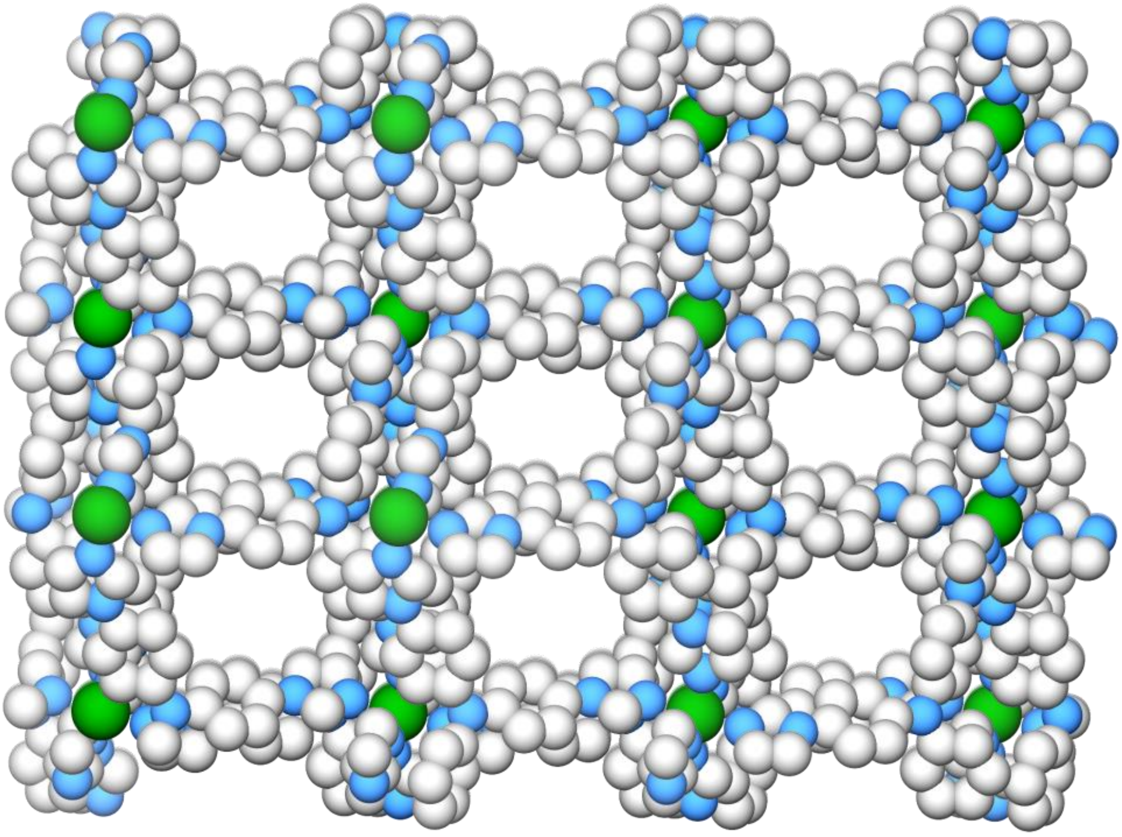

Figure S3: Structural Features of IPM-MOF-201, related to Figure 1.

Packing diagram of **IPM-MOF-201** showing porous channel. (Hydrogen atoms and disordered anions have been omitted for clarity, colour: Gray - C, Blue - N, Green - Ni).

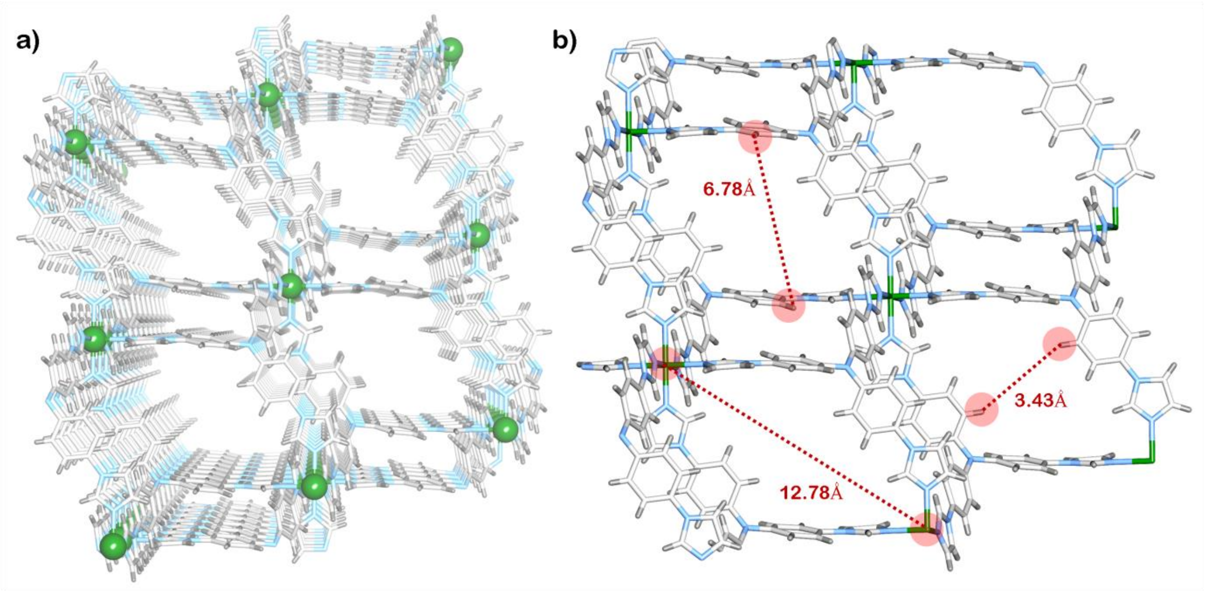

Figure S4: Structural Features of IPM-MOF-201, related to Figure 1.

a) Packing diagram of **IPM-MOF-201** showing porous channels, b) View of a single net adopted from the packing diagram showing the dimensions of the pore. The dimensions have included the Van der Waal radii of the atoms chosen for measurement. The measurements suggest that the d_{min} and d_{max} for the compound is 3.43Å and 12.78Å respectively. (Disordered anions have been omitted for clarity, colour: Light Gray - C, Dark Gray - H, Blue - N, Green - Ni).

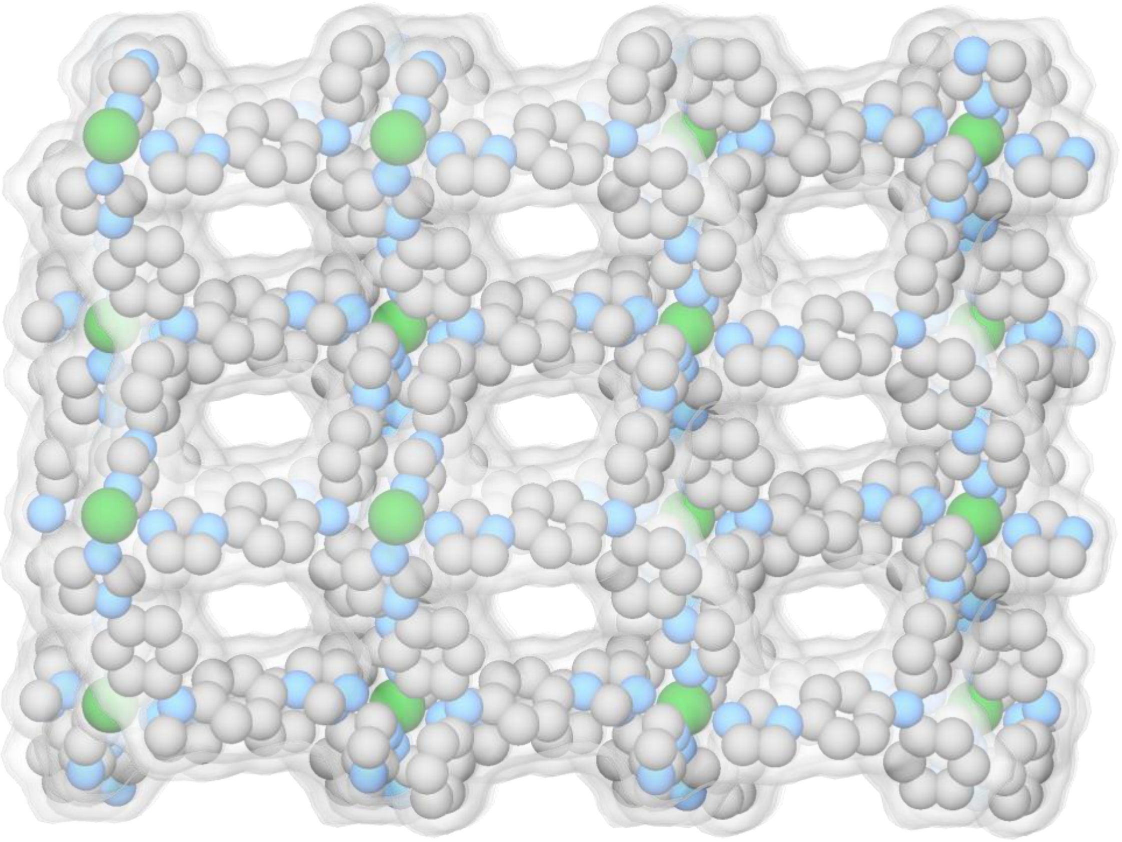

Figure S5: Structural Features of IPM-MOF-201, related to Figure 1.

Packing diagram of **IPM-MOF-201**. (Hydrogen atoms and disordered anions have been omitted for clarity, colour: Gray - C, Blue - N, Green - Ni).

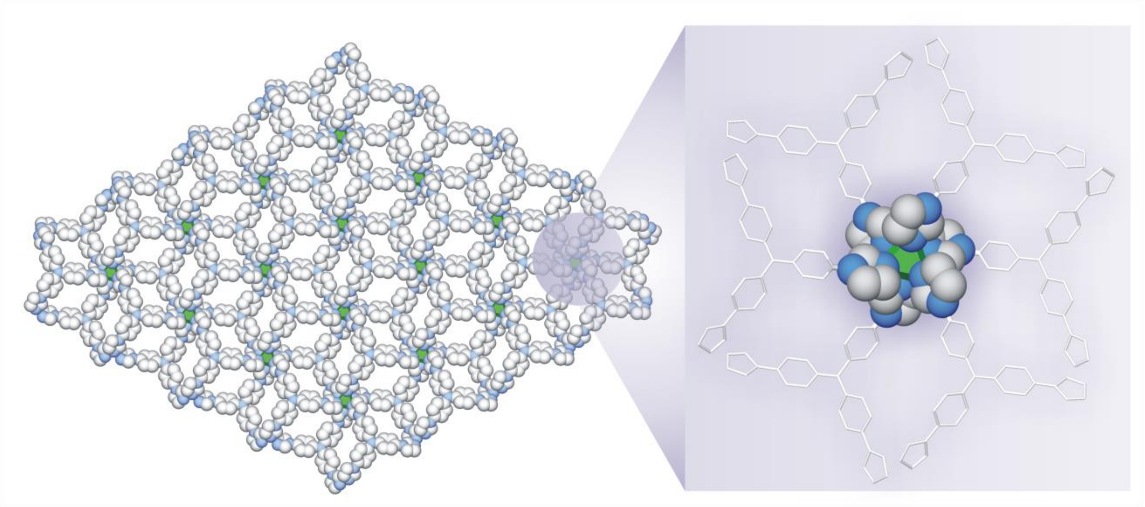

Figure S6: Structural Features of IPM-MOF-201, related to Figure 1.

Packing diagram of **IPM-MOF-201** and zoomed view of the coordination environment of the metal node. (Hydrogen atoms and disordered anions have been omitted for clarity, colour: Gray - C, Blue - N, Green - Ni).

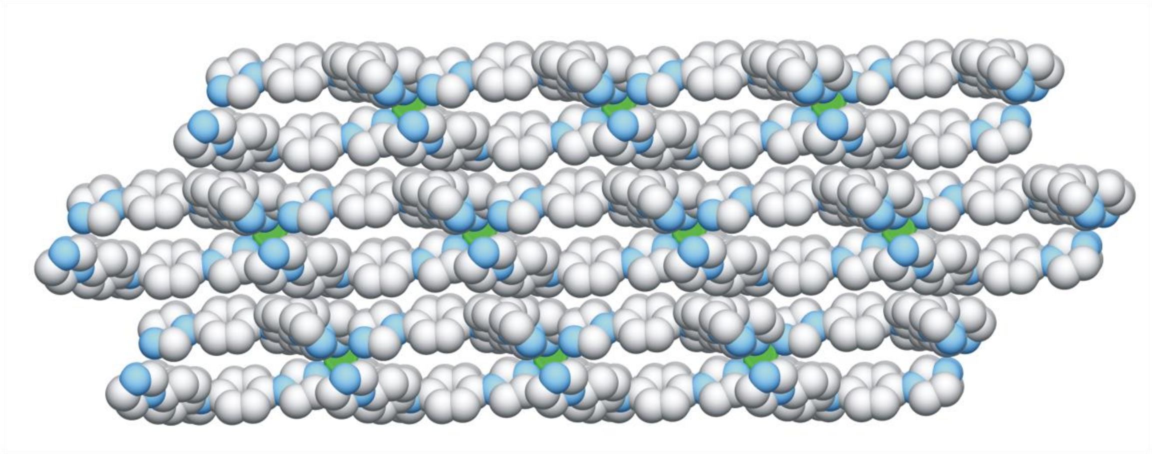

Figure S7: Structural Features of IPM-MOF-201, related to Figure 1.

Packing diagram of **IPM-MOF-201** showing depicting the metal nodes are buried in the 2D layer stacking arrangement. (Hydrogen atoms and disordered anions have been omitted for clarity, colour: Gray - C, Blue - N, Green - Ni).

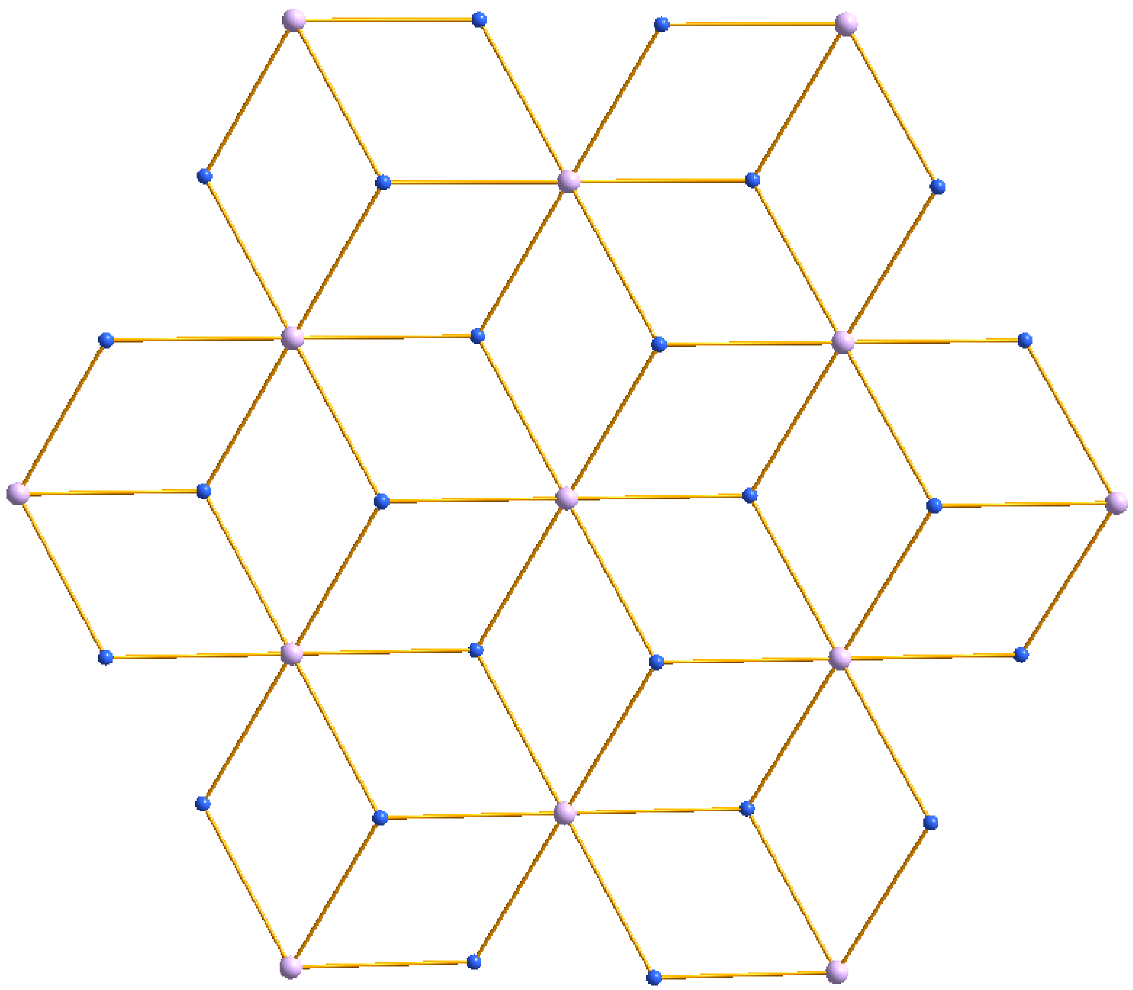

Figure S8: Structural Features of IPM-MOF-201, related to Figure 1.

Simplified topological representation of **IPM-MOF-201** having kgd topology.

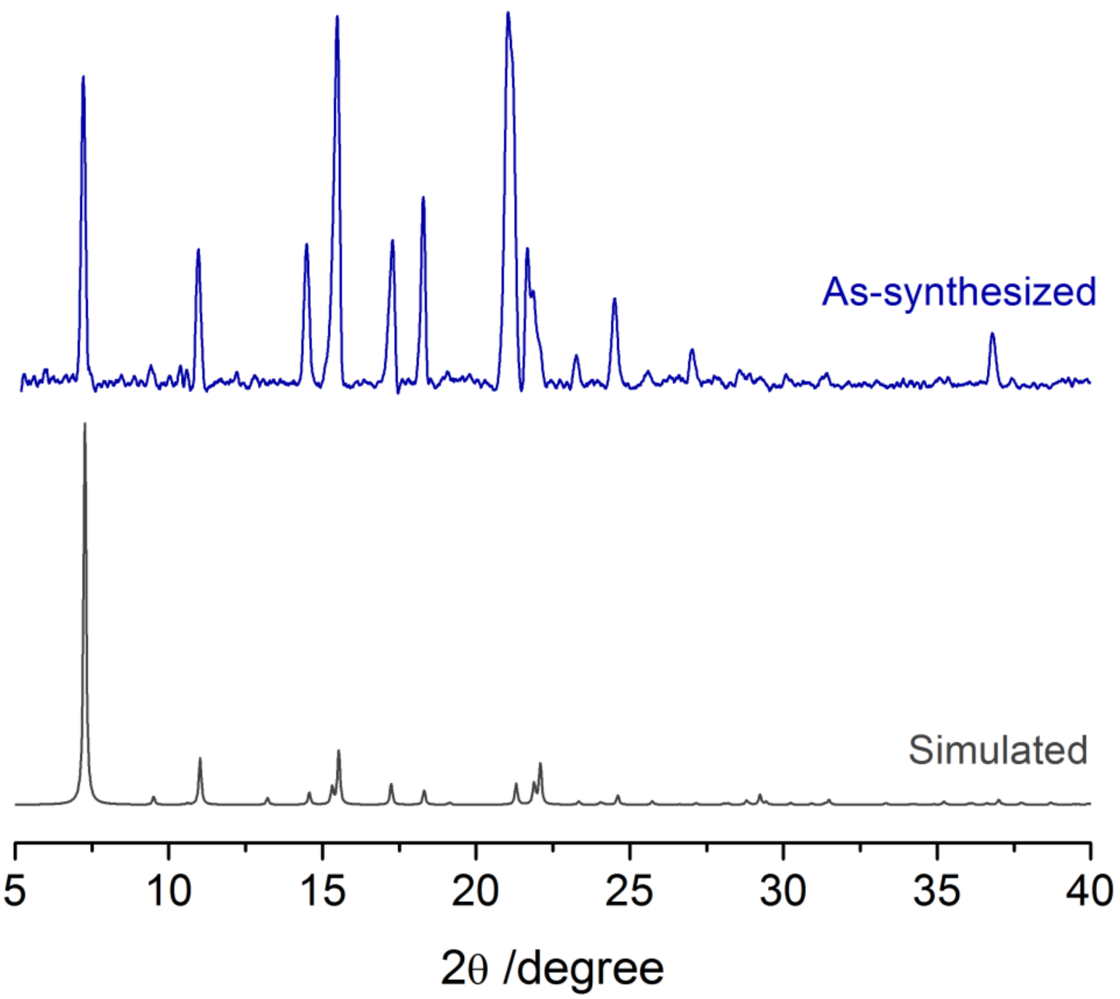

Figure S9: Characterization of IPM-MOF-201, related to Figure 1.

Powder X-ray diffraction patterns of **IPM-MOF-201** - Simulated (gray), as-synthesized (blue).

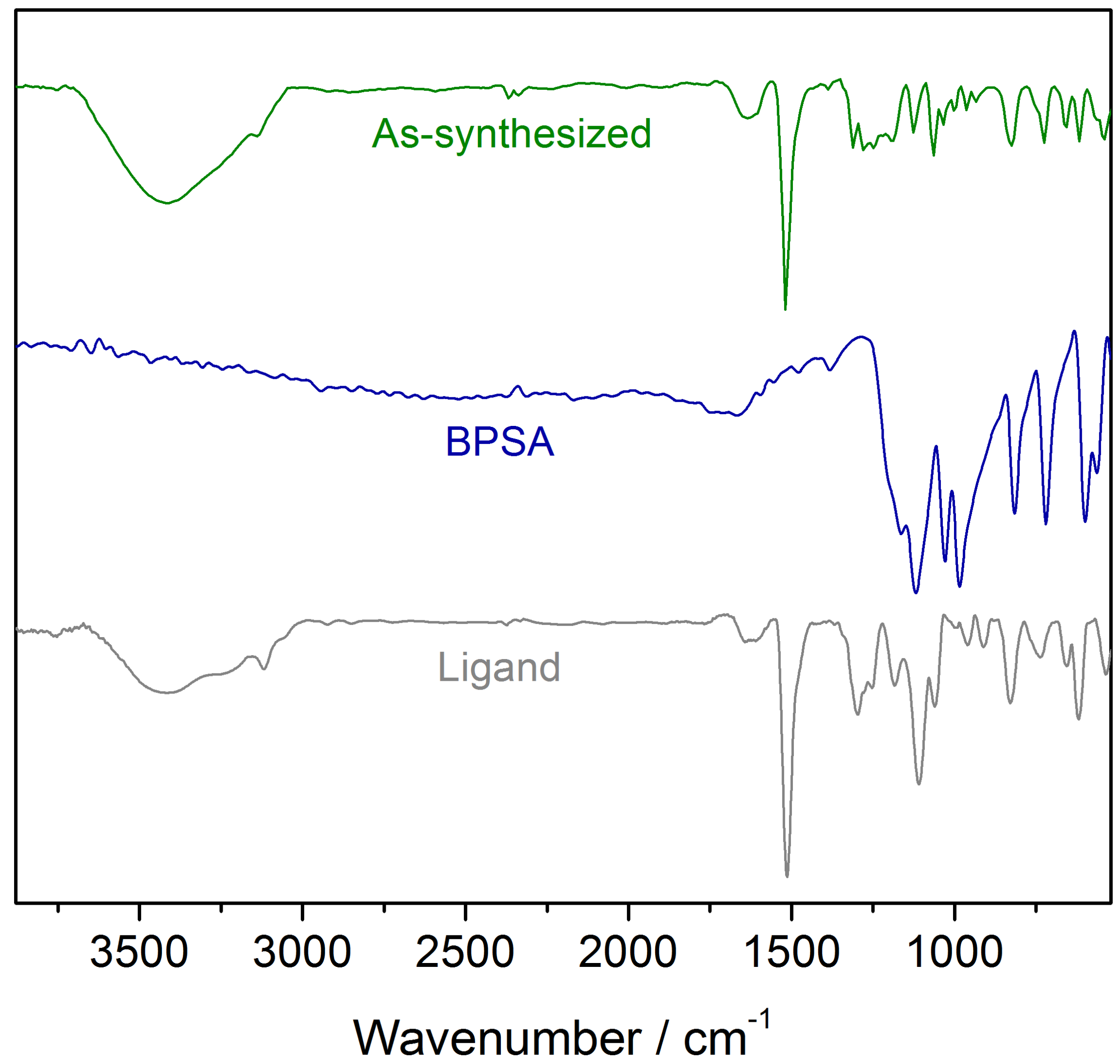

Figure S10: Characterization of IPM-MOF-201, related to Figure 1.
FT-IR spectra for compound **IPM-MOF-201** (green), BPSA (blue) and ligand (gray).

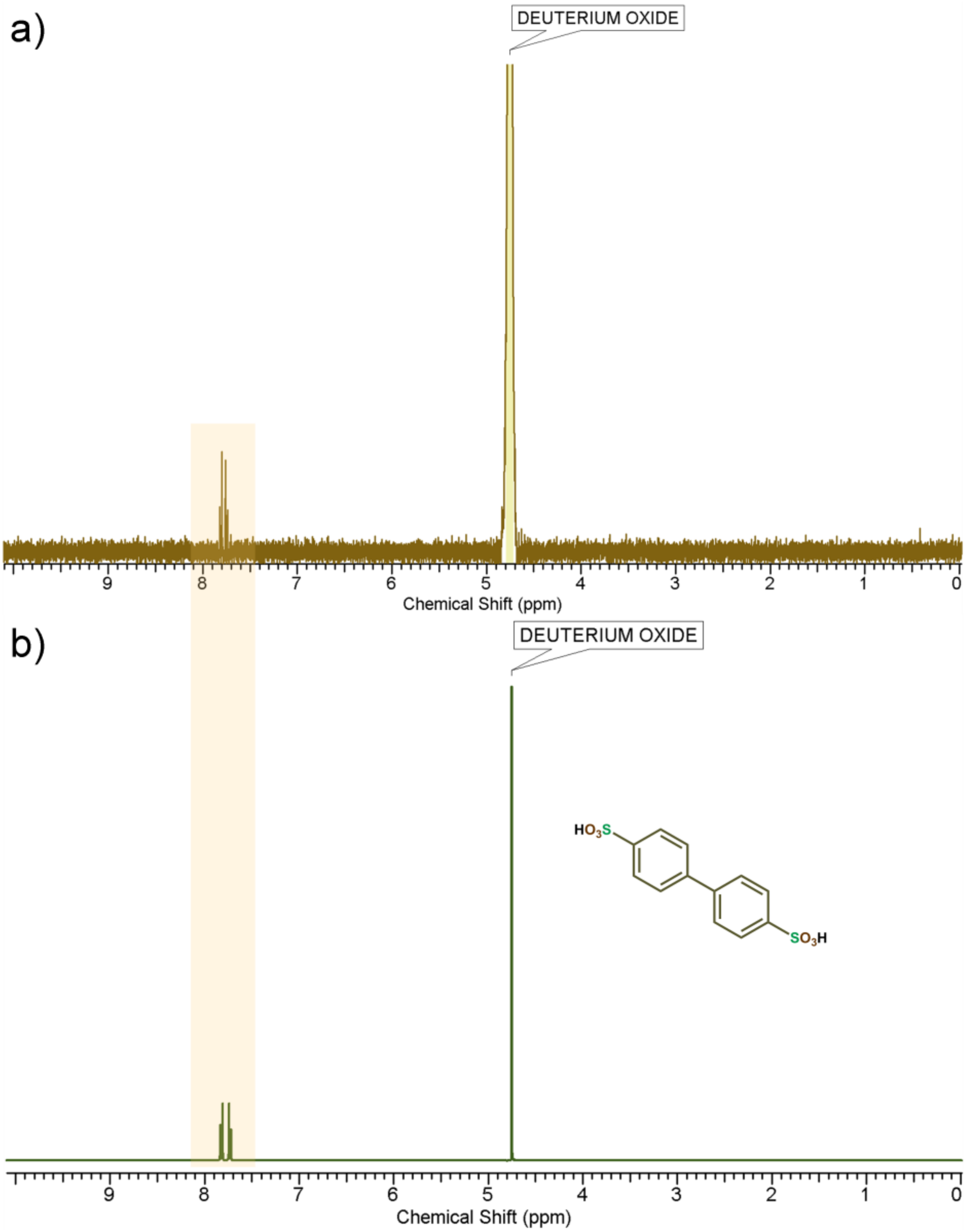

Figure S11: Characterization of IPM-MOF-201, related to Figure 1.
a) ^{1}H-NMR after digesting **IPM-MOF-201** in D_3PO_4/D_2O followed by neutralization with NaOD. b) ^{1}H-NMR of commercially available BPSA in D_2O.

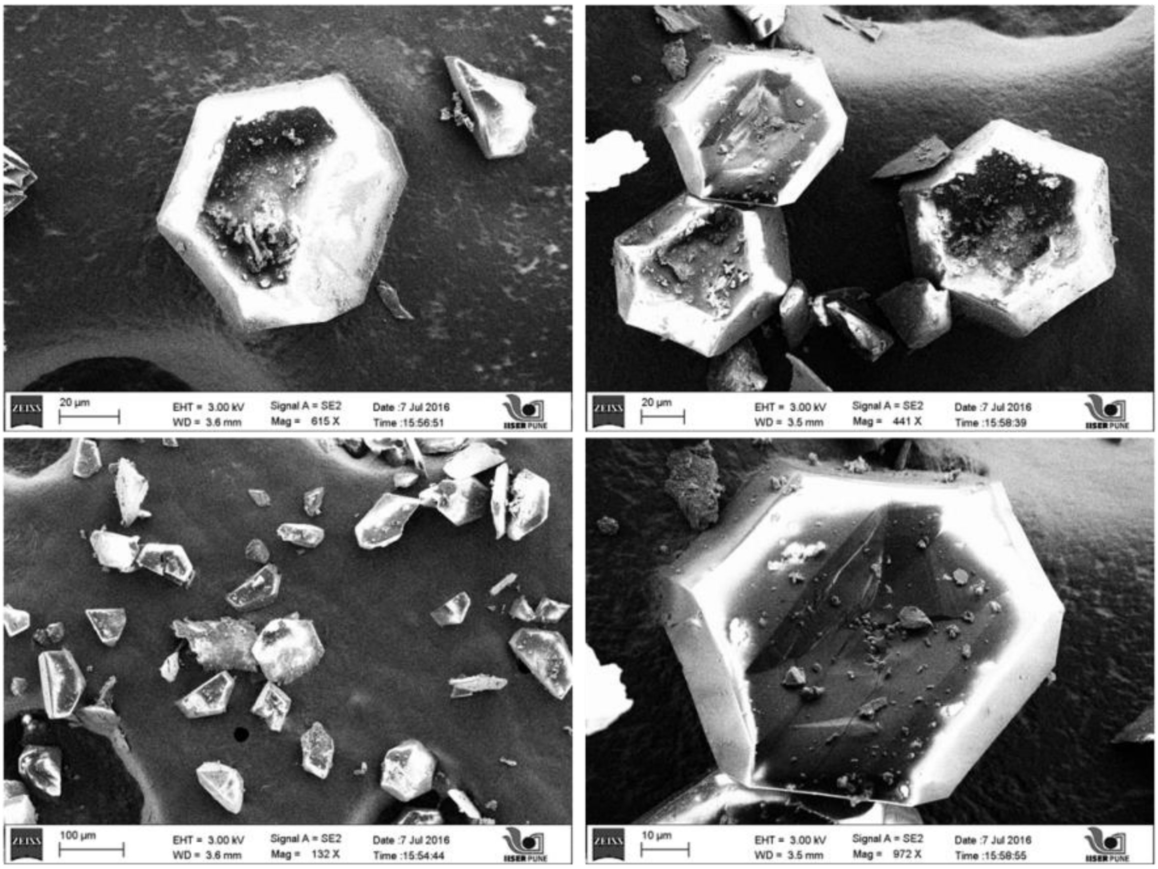

Figure S12: Characterization of IPM-MOF-201, related to Figure 1.
FESEM images for compound **IPM-MOF-201**.

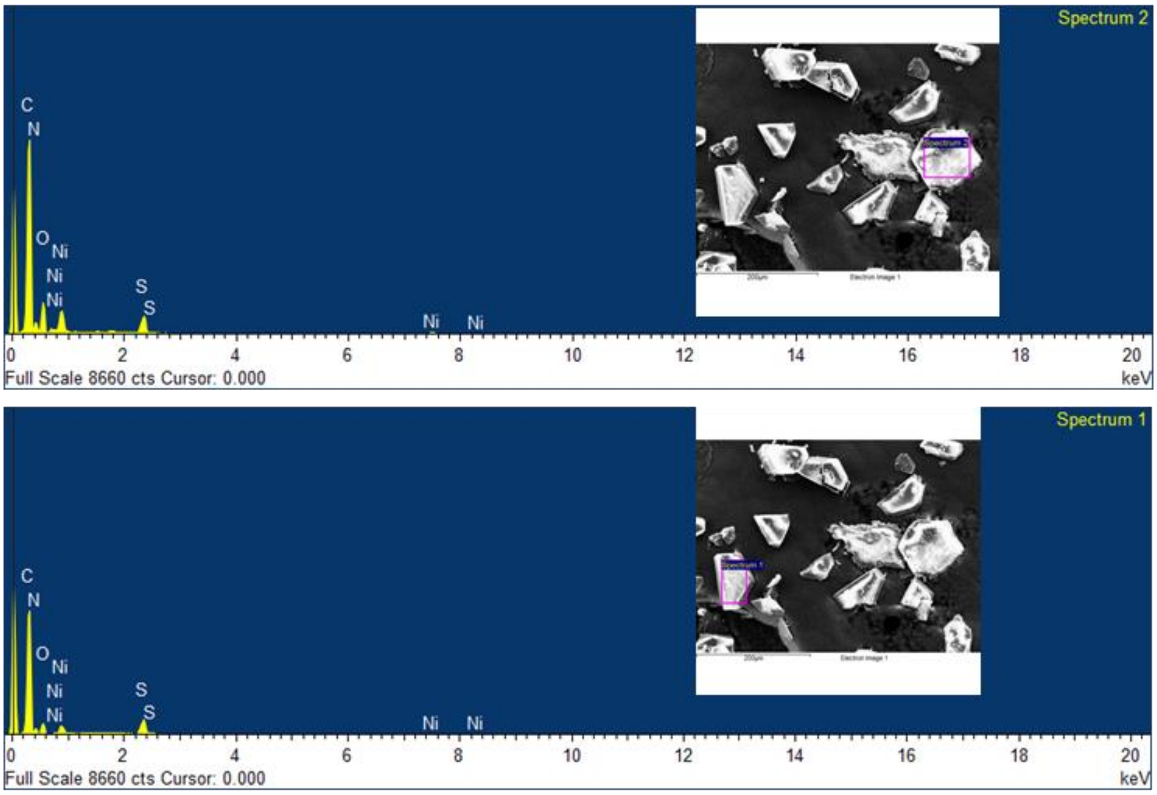

Figure S13: Characterization of IPM-MOF-201, related to Figure 1.
EDX spectra for compound **IPM-MOF-201**.

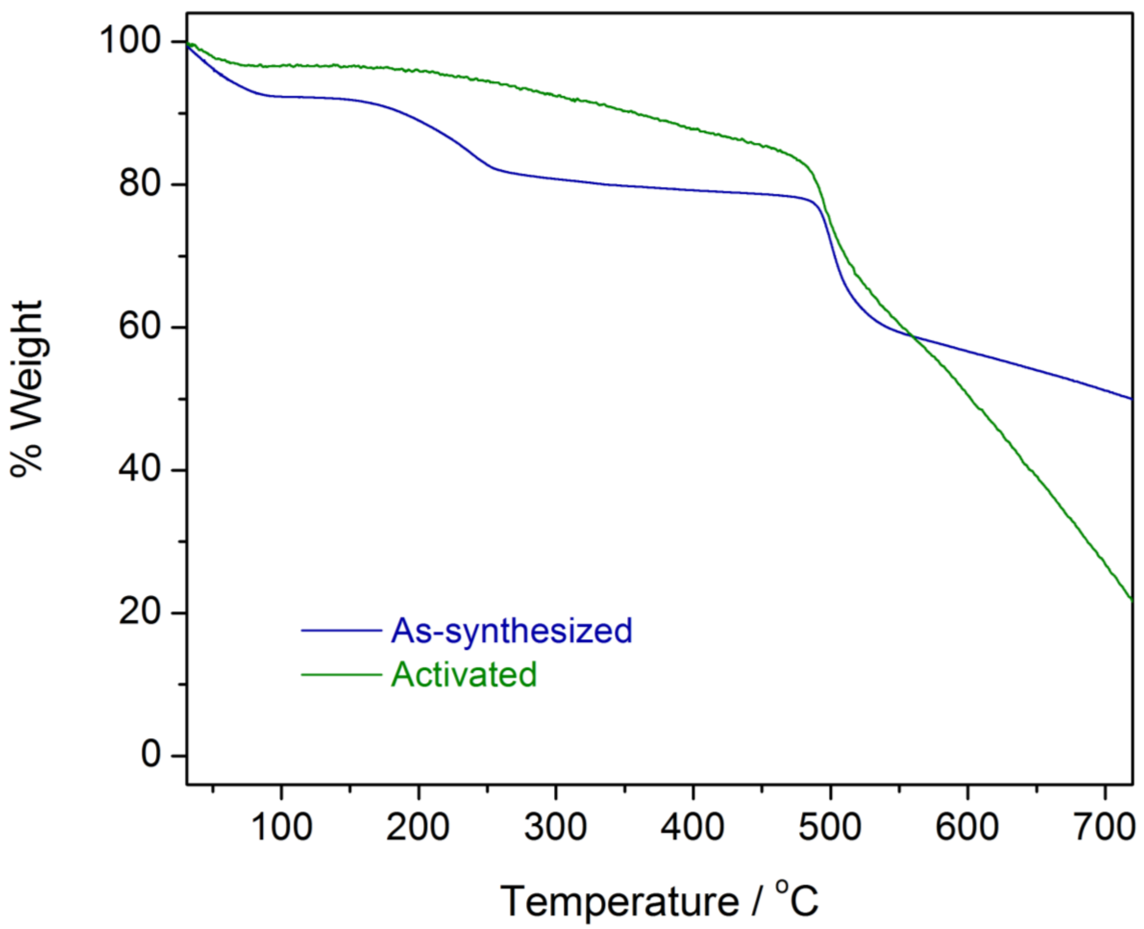

Figure S14: Characterization of IPM-MOF-201, related to Figure 1.
TGA profiles for compound **IPM-MOF-201**.

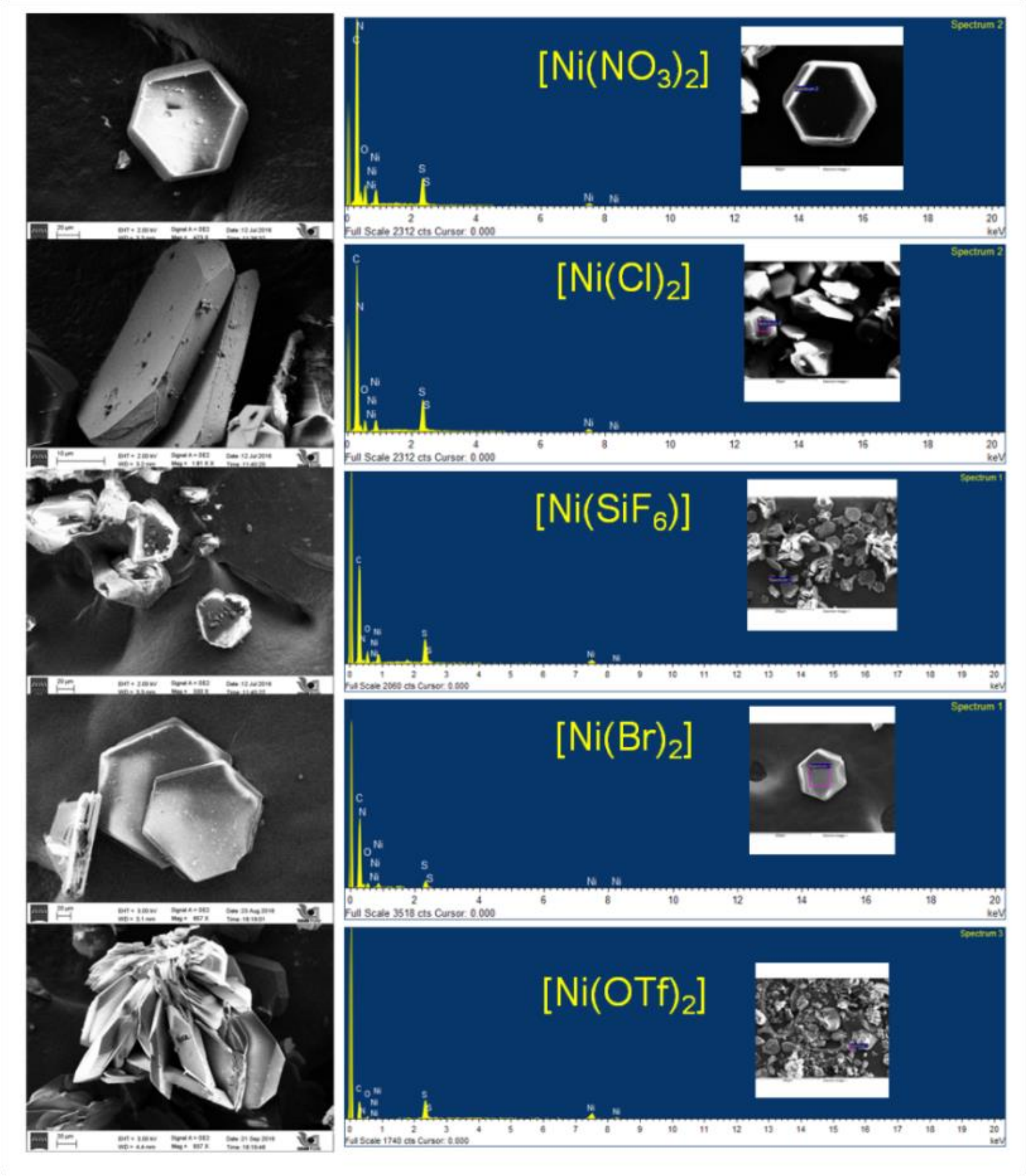

Figure S15: Characterization of IPM-MOF-201, related to Figure 1.
EDX spectra for compounds synthesized using different salts of Ni^{2+} and BPSA (excess).

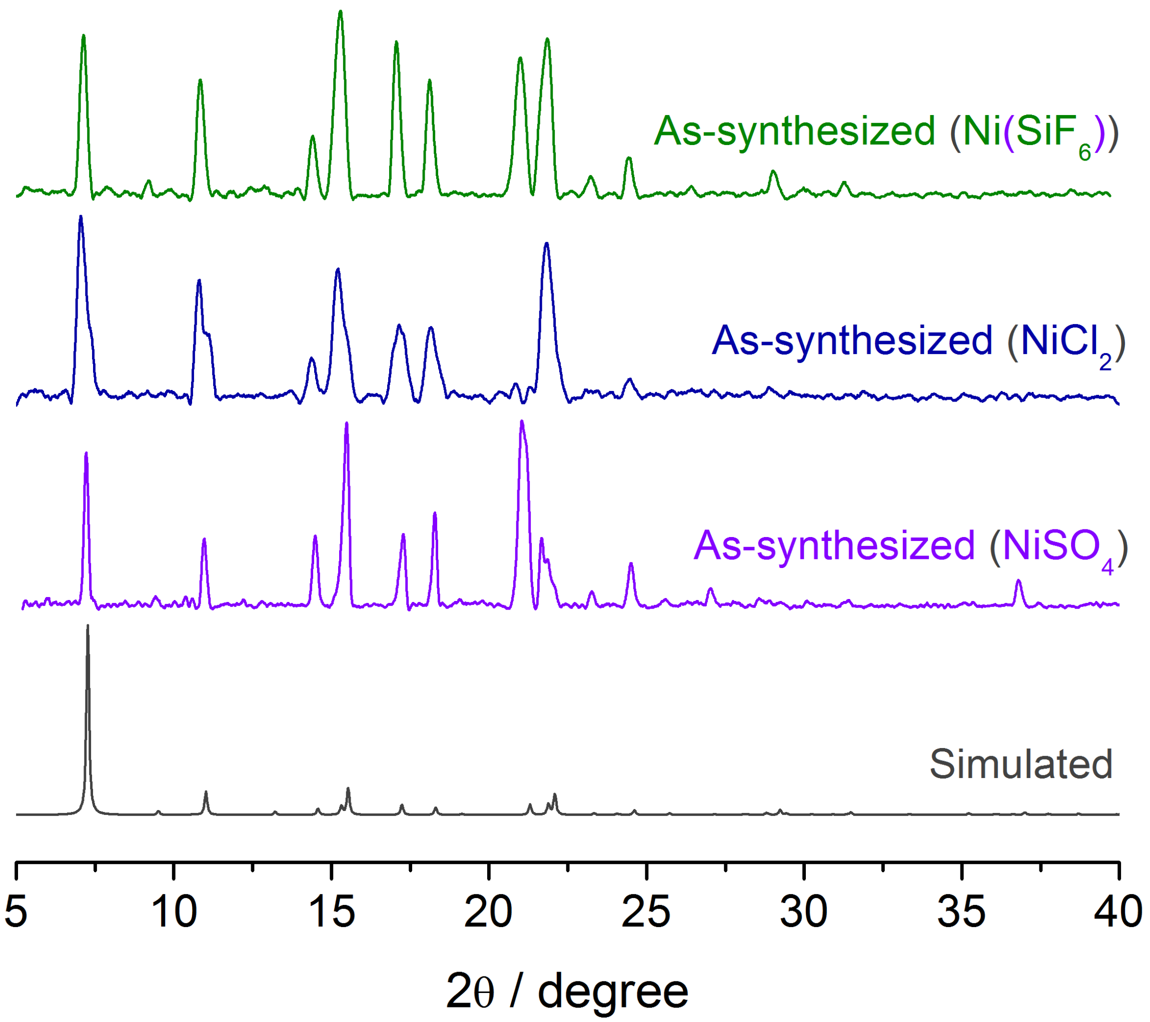

Figure S16: Characterization of IPM-MOF-201, related to Figure 1.
Powder X-ray diffraction patterns for compounds synthesized using different salts of Ni^{2+} and BPSA (excess).

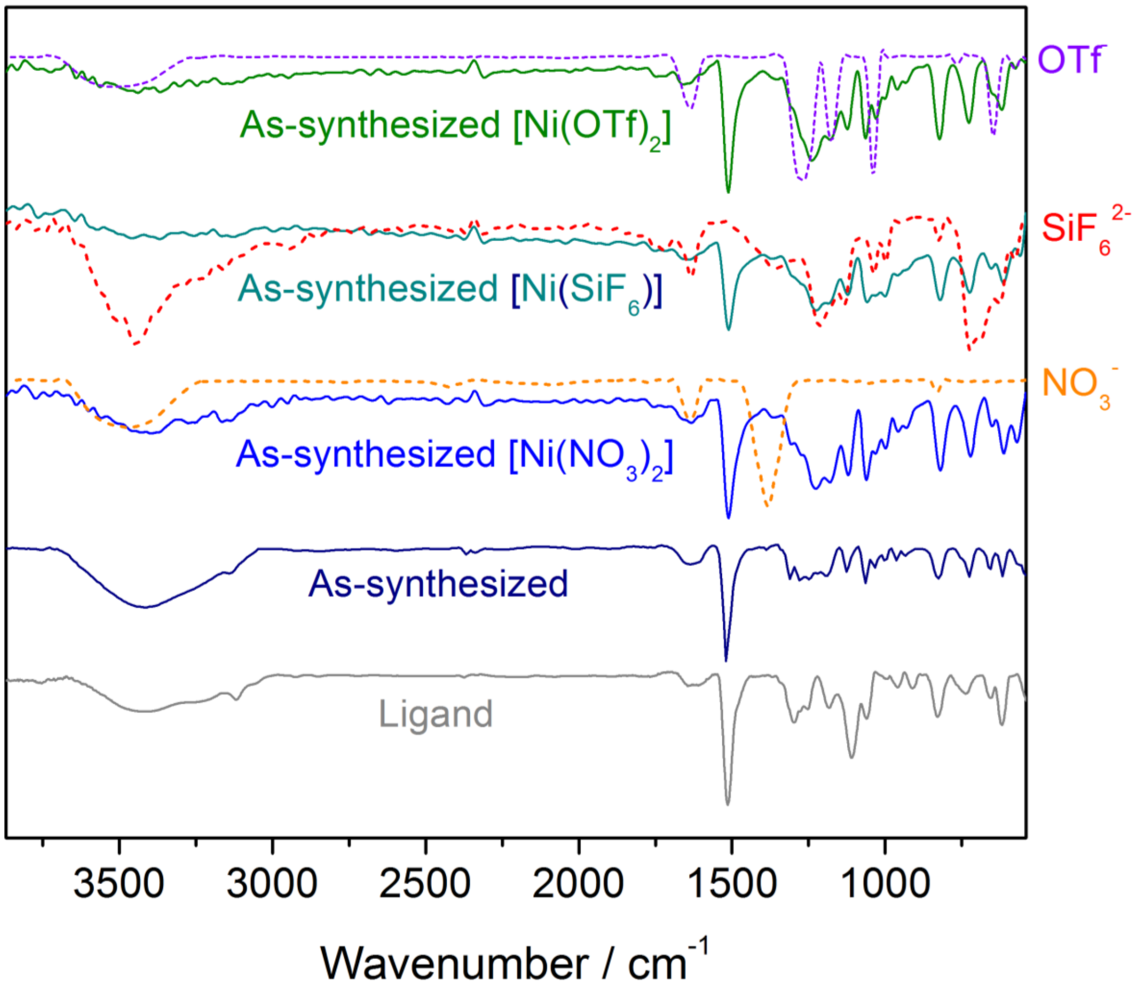

Figure S17: Characterization of IPM-MOF-201, related to Figure 1.
FT-IR spectra for compounds synthesized using different salts of Ni^{2+} and BPSA (excess). Dotted lines represent spectra for salts of respective anions.

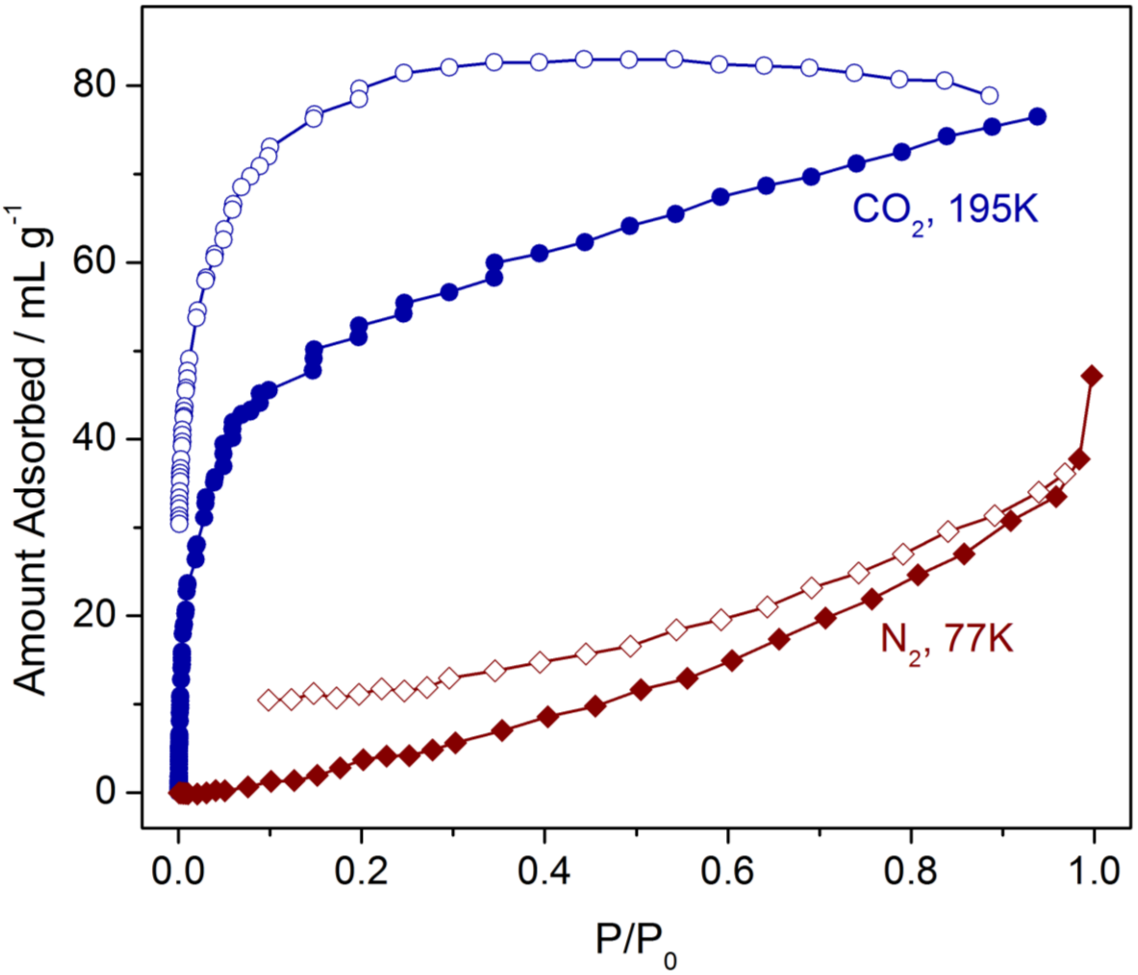

Figure S18: Gas adsorption of IPM-MOF-201, related to Figure 1.
Low temperature gas adsorption isotherms for compound **IPM-MOF-201**. Closed symbols denote adsorption while open symbols denote desorption.

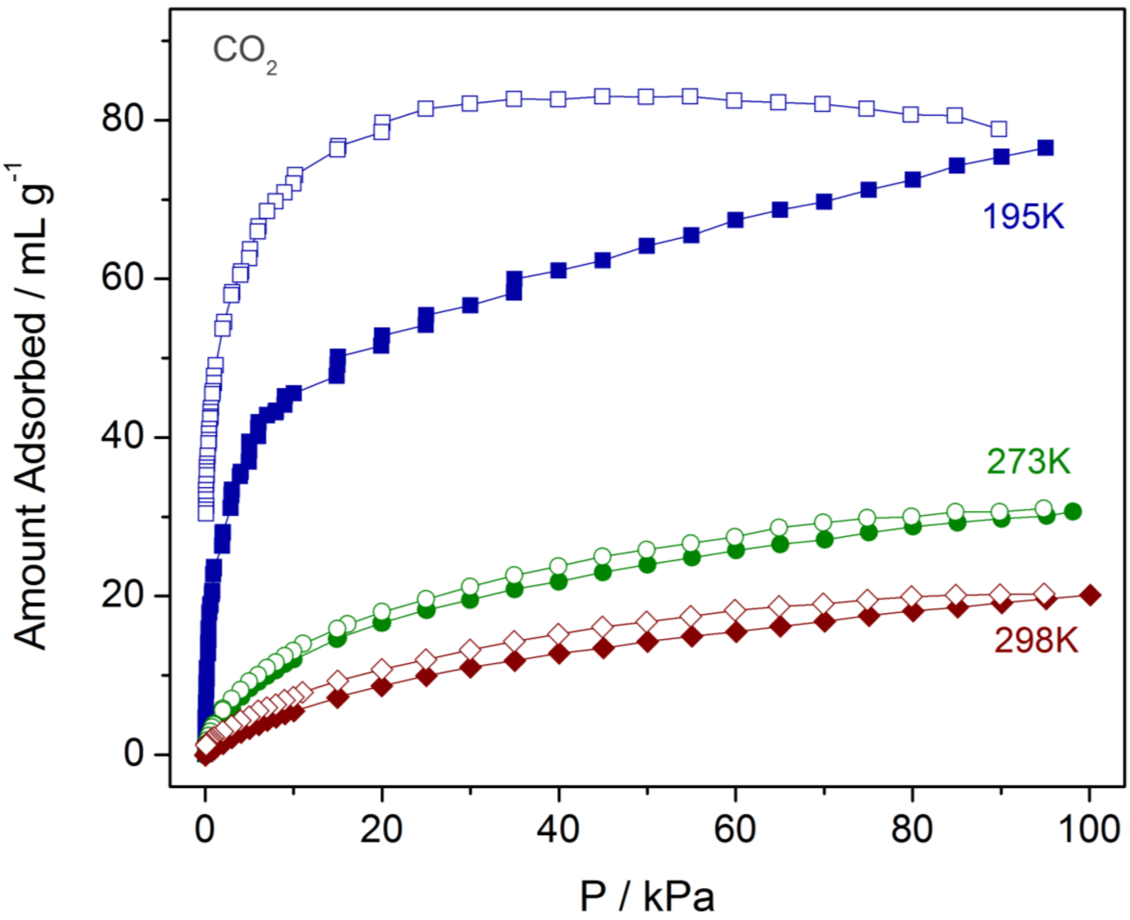

Figure S19: Gas adsorption of IPM-MOF-201, related to Figure 1.
CO_2 adsorption isotherms for compound **IPM-MOF-201** at different temperatures; 195K (blue), 273K (green), 298K (wine red). Closed symbols denote adsorption while open symbols denote desorption.

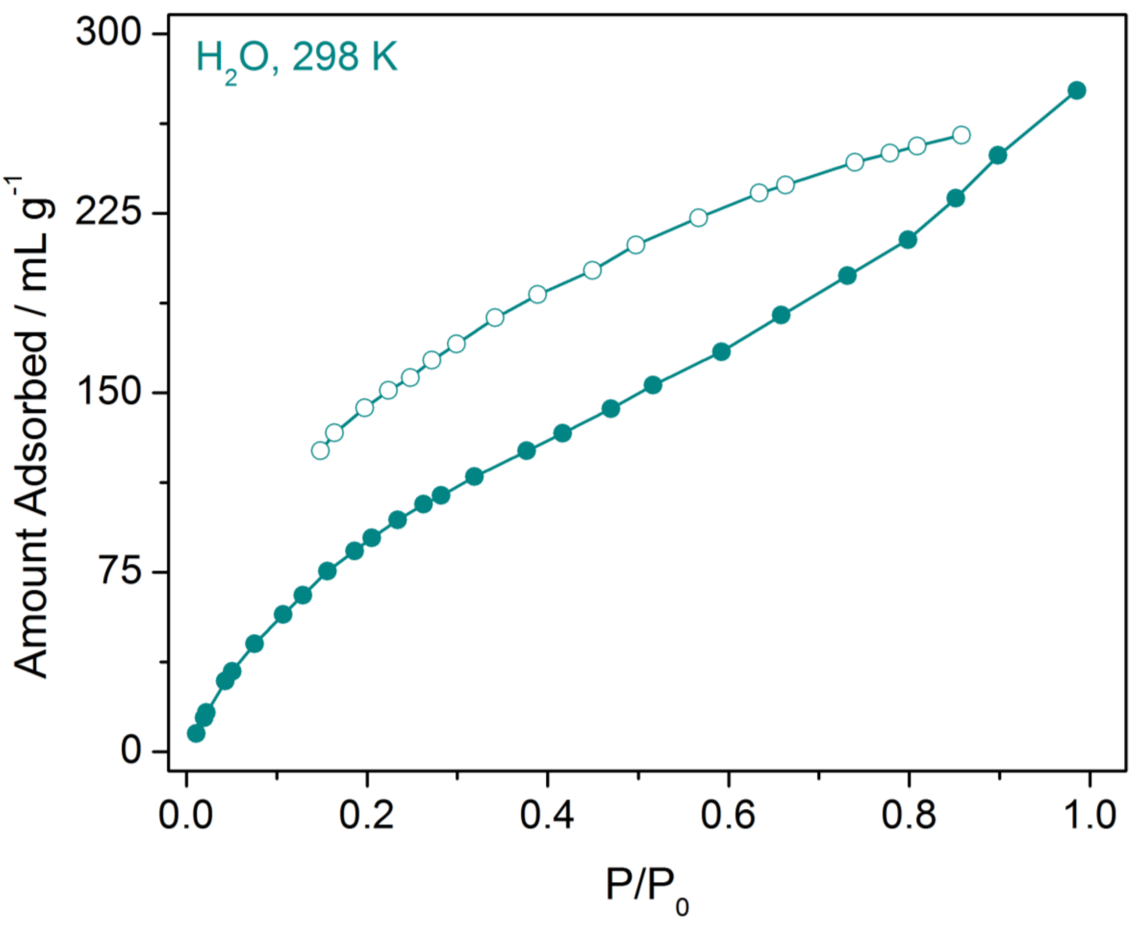

Figure S20: Vapour adsorption of IPM-MOF-201, related to Figure 2.
Water adsorption isotherm for compound **IPM-MOF-201** at 298K. Closed symbols denote adsorption while open symbols denote desorption.

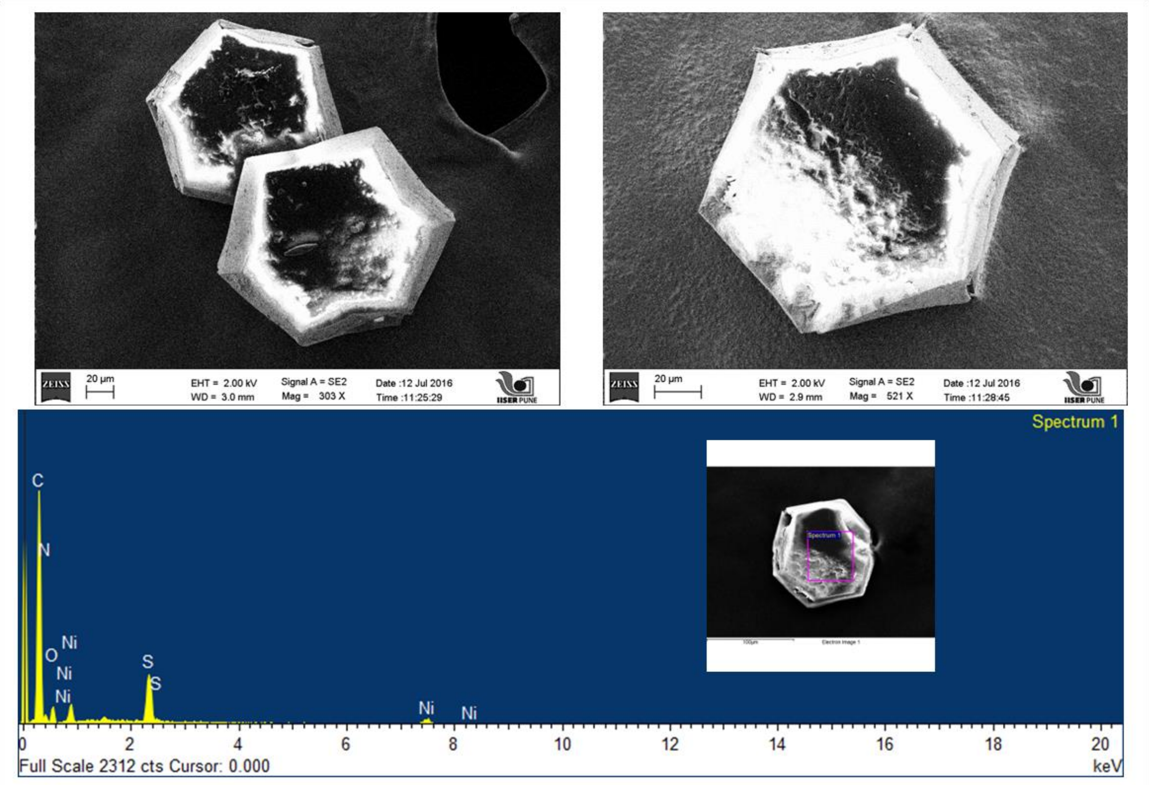

Figure S21: Stability Studies of IPM-MOF-201, related to Figure 2.

FESEM images (top) and EDX spectra (below; inset sample chosen for recording the EDX spectra) for compound **IPM-MOF-201** dipped in D.I. water for 1 month.

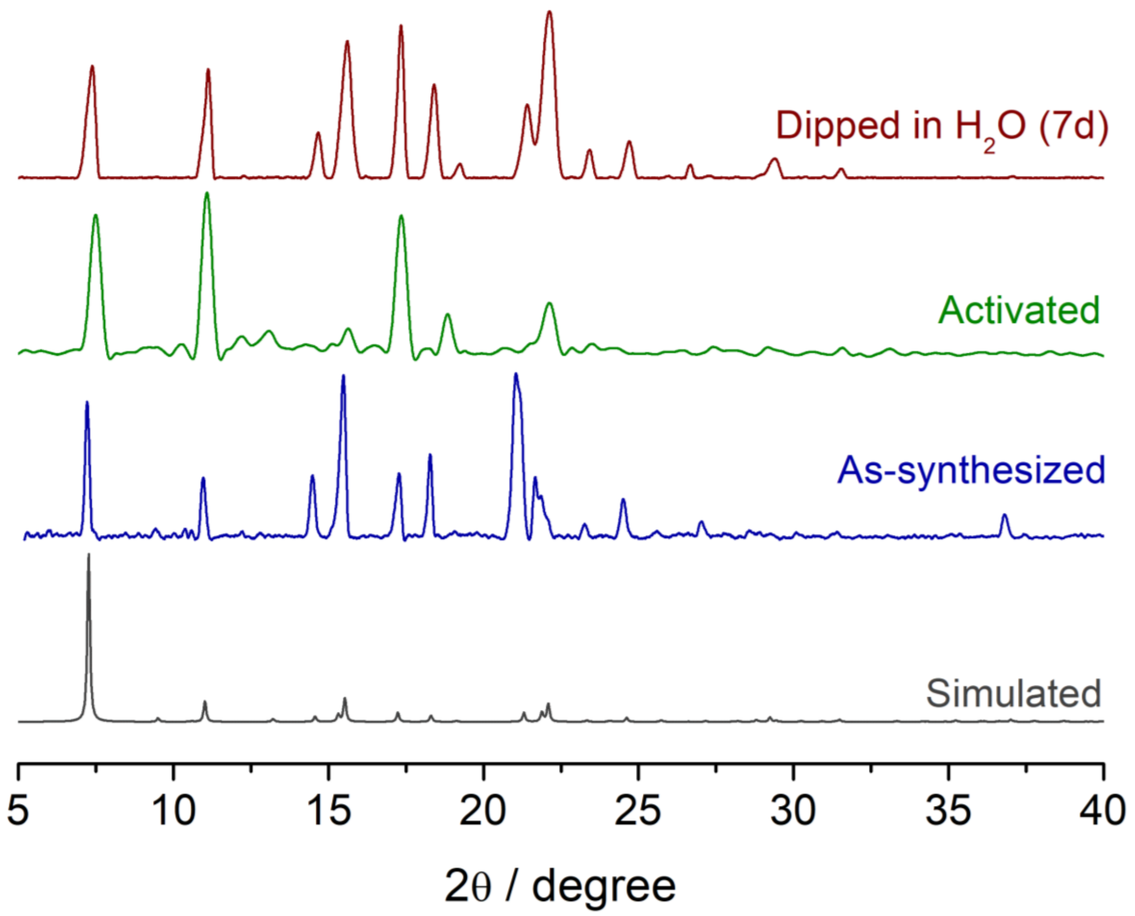

Figure S22: Stability Studies of IPM-MOF-201, related to Figure 2.
PXRD patterns for simulated (grey), as-synthesized (blue), activated (green) and water-dipped phases (wine red).

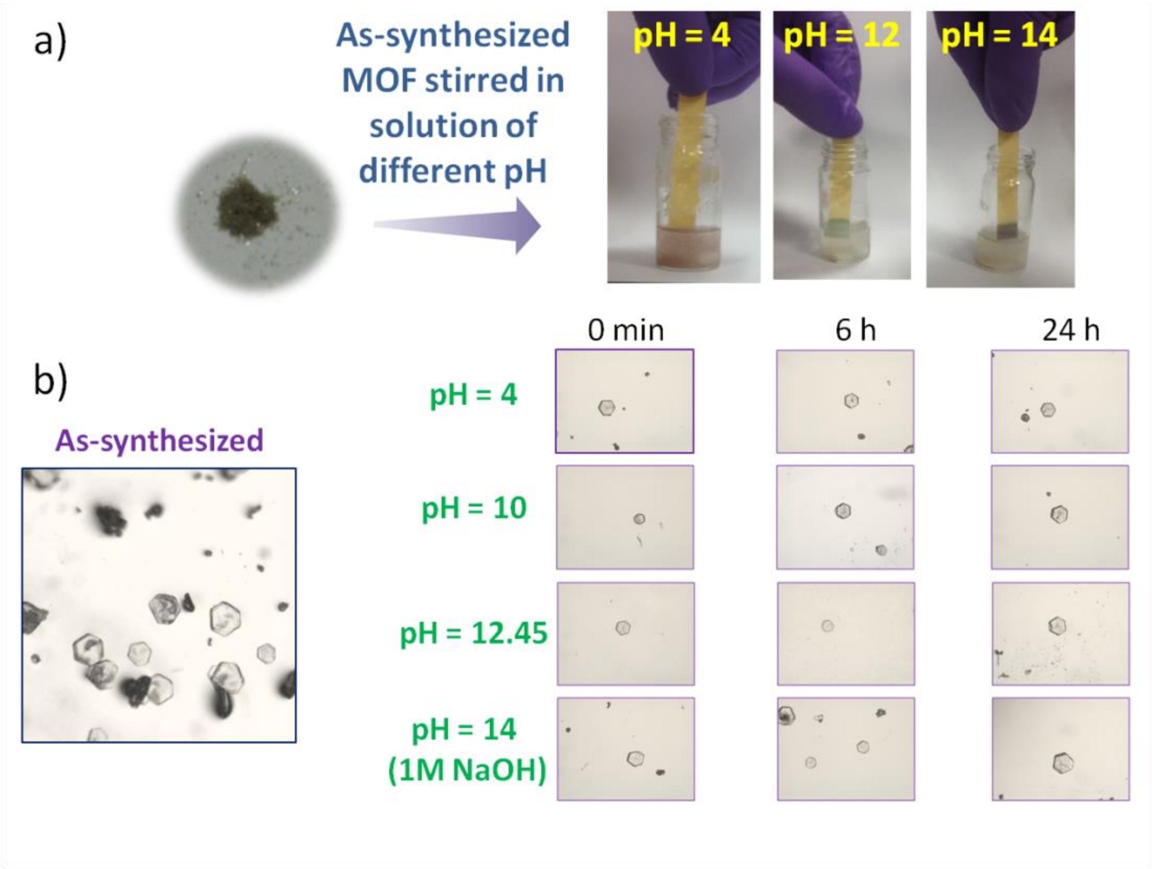

Figure S23: Stability Studies of IPM-MOF-201, related to Figure 2.
a) Photographs showing the experiment performed to check pH resistance, b) photographs taken under microscope for as-synthesized phase and the crystals upon dipping in solutions of different pH at varying time intervals.

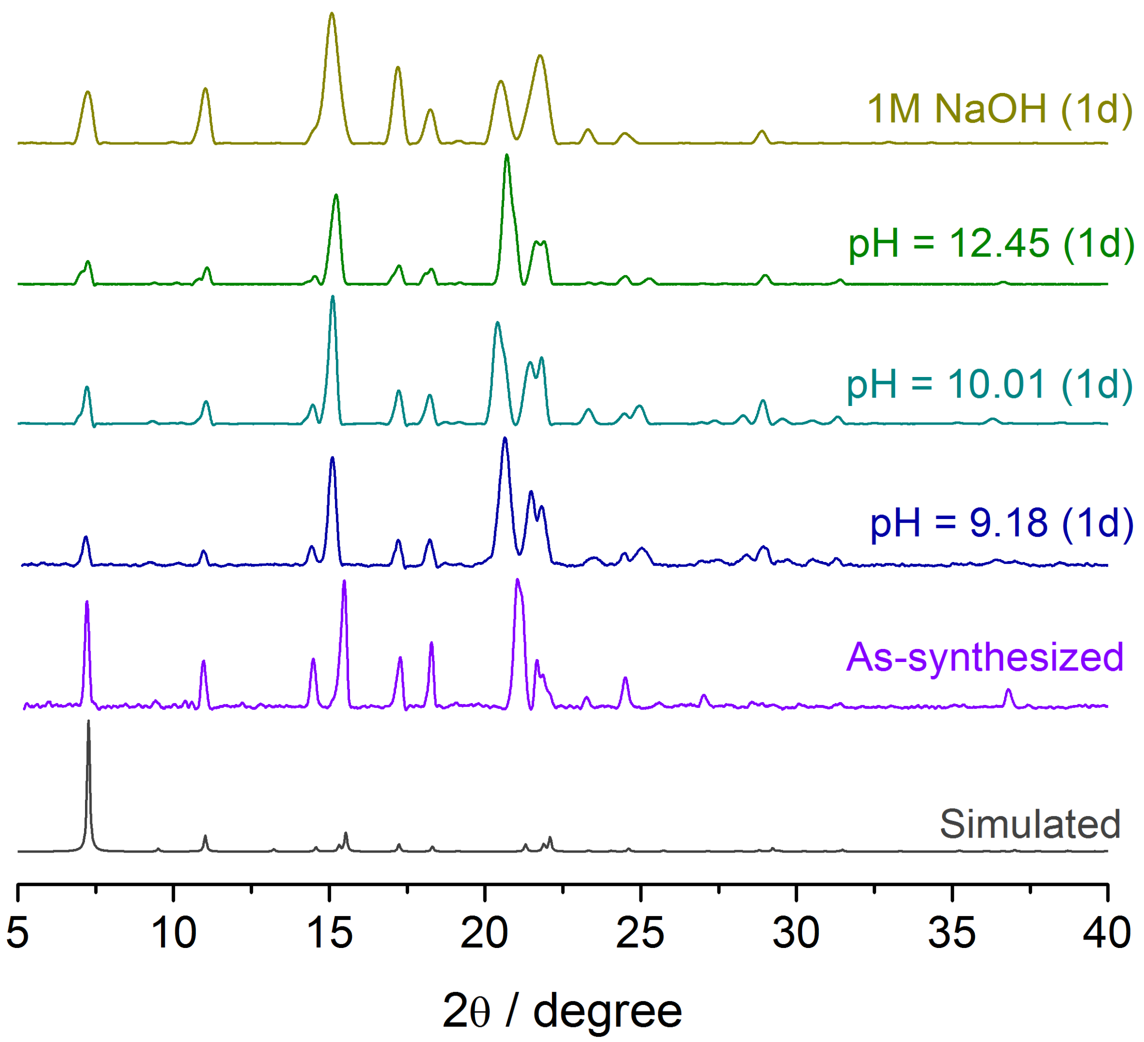

v

Figure S24: Stability Studies of IPM-MOF-201, related to Figure 2.
Powder x-ray diffractions patterns for simulated (grey), as-synthesized (purple), and compound **IPM-MOF-201** dipped in different pH conditions.

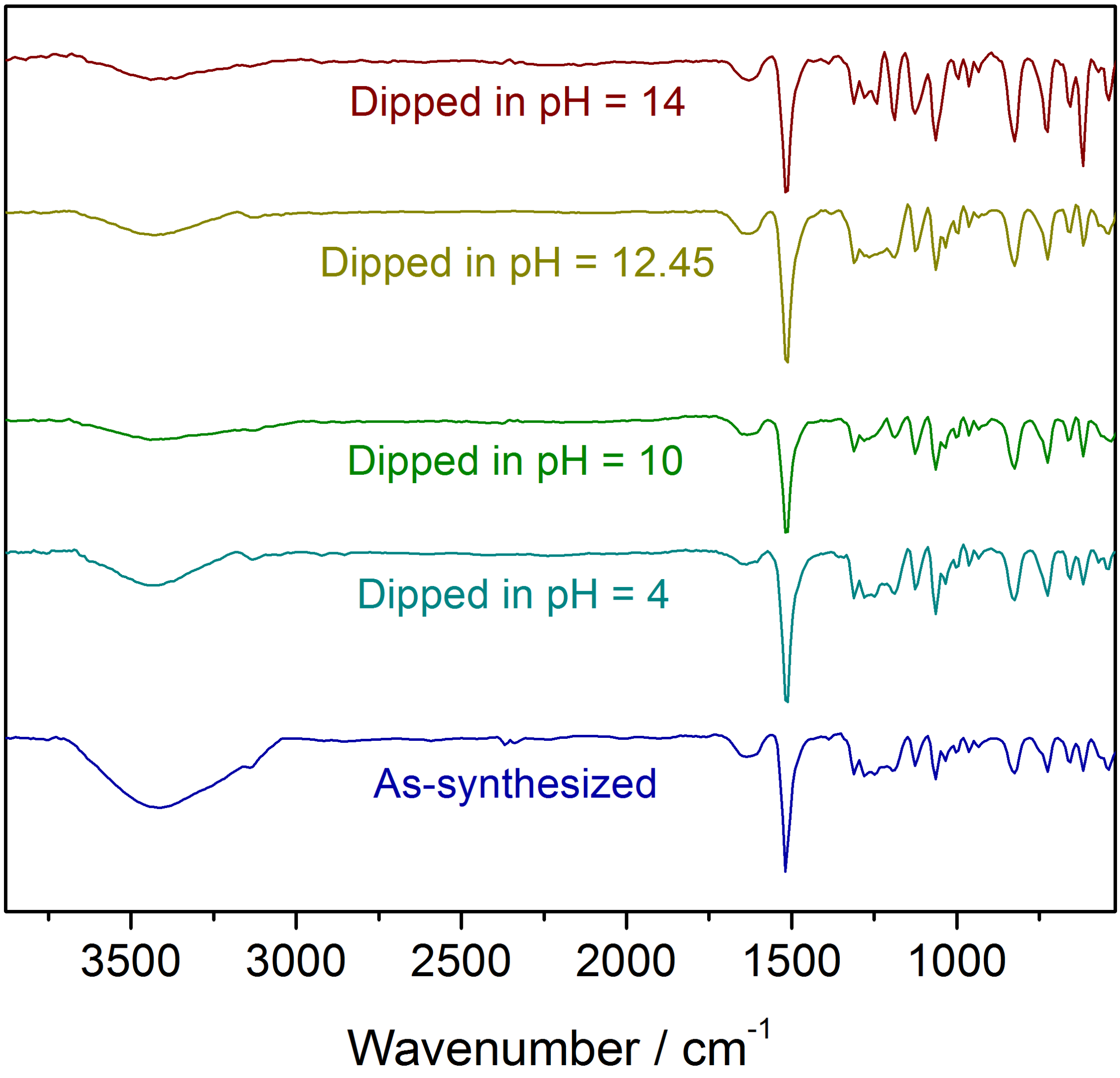

Figure S25: Stability Studies of IPM-MOF-201, related to Figure 2.
FT-IR spectra for **IPM-MOF-201** dipped in different pH solutions.

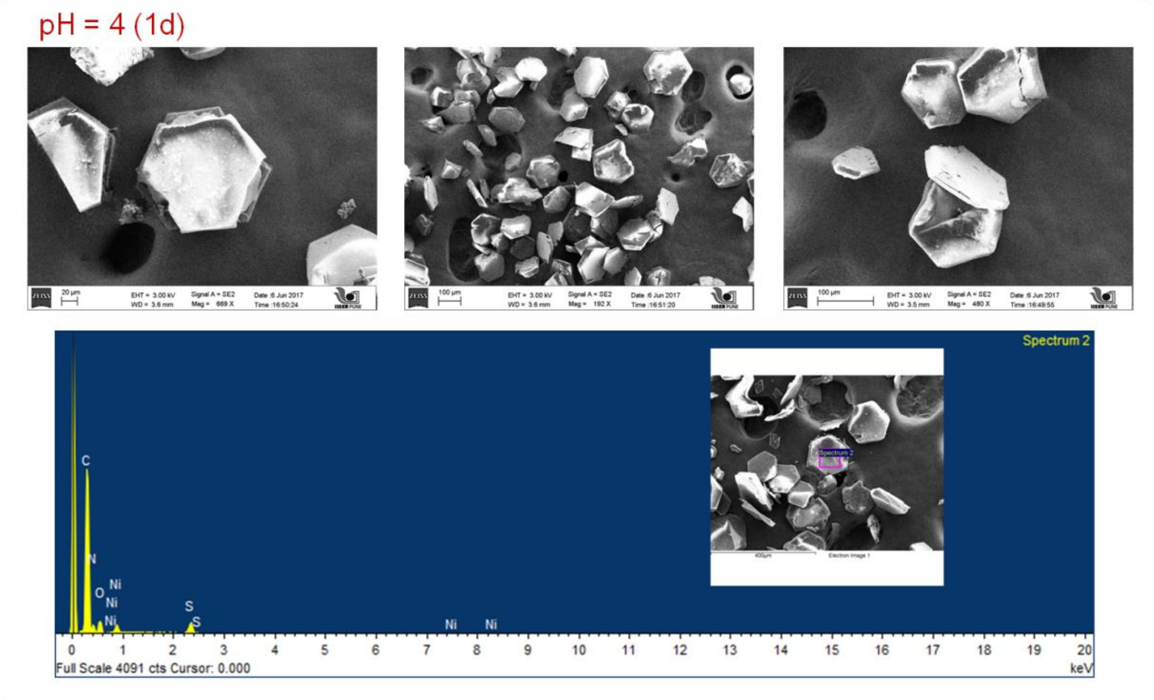

Figure S26: Stability Studies of IPM-MOF-201, related to Figure 2.
FESEM images of compound **IPM-MOF-201** dipped in solution of pH = 4 & corresponding EDX profile.

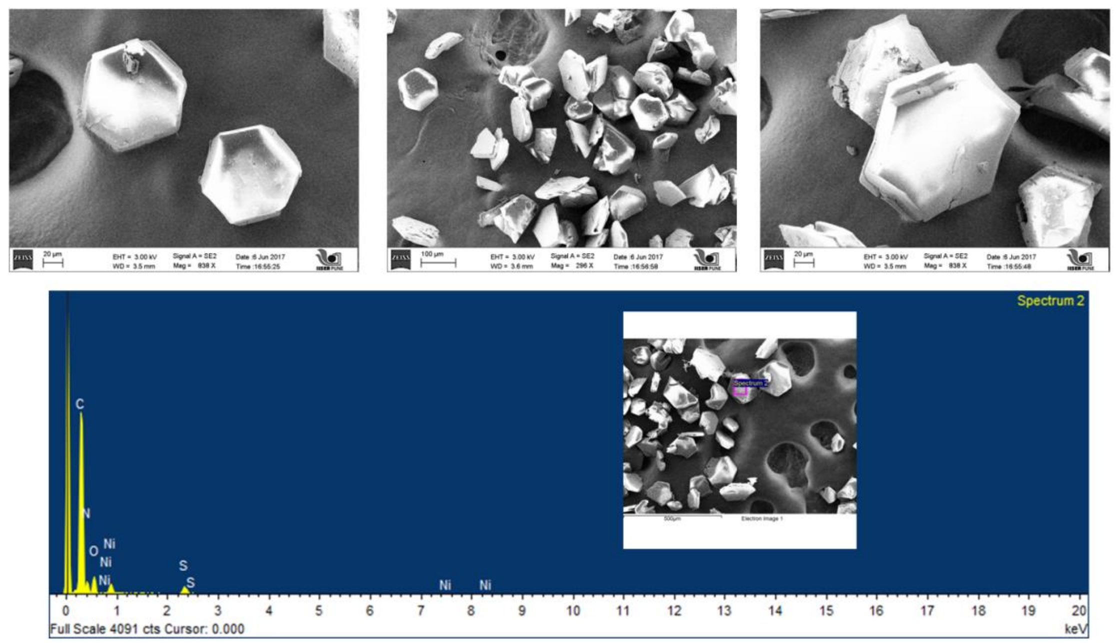

Figure S27: Stability Studies of IPM-MOF-201, related to Figure 2.

FESEM images of compound **IPM-MOF-201** dipped in solution of pH = 10.01 & corresponding EDX profile.

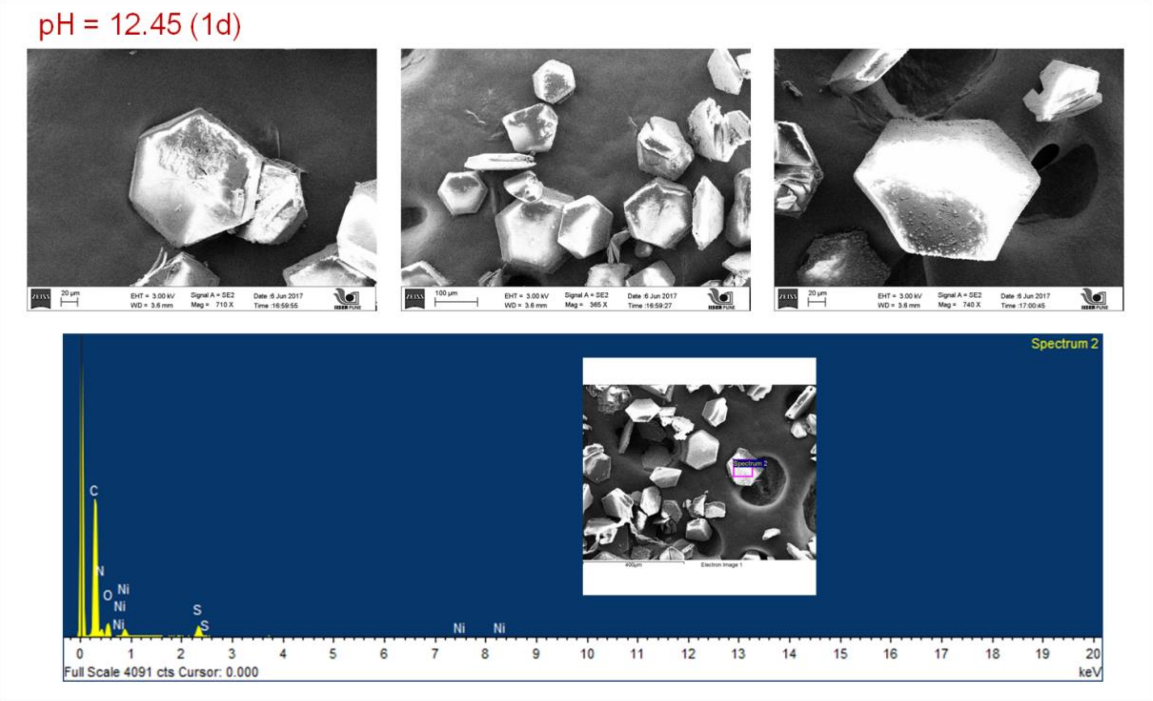

Figure S28: Stability Studies of IPM-MOF-201, related to Figure 2.
FESEM images of compound **IPM-MOF-201** dipped in solution of pH = 12.45 & corresponding EDX profile.

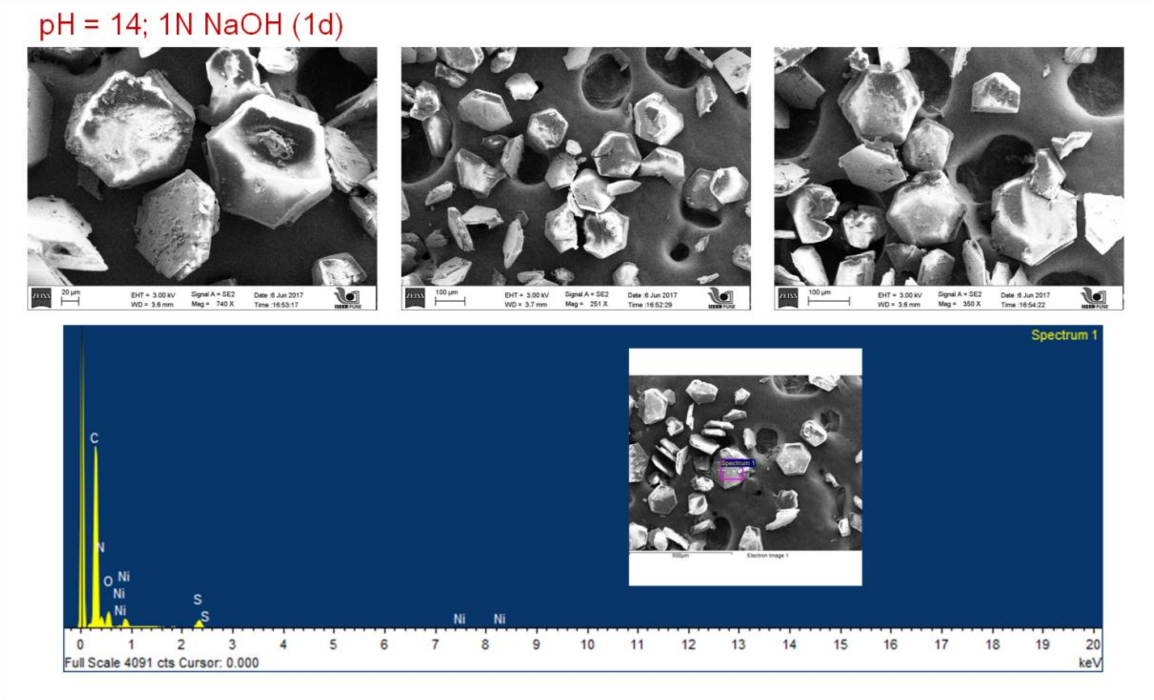

Figure S29: Stability Studies of IPM-MOF-201, related to Figure 2.
FESEM images of compound **IPM-MOF-201** dipped in solution of pH = 14 (1N NaOH) & corresponding EDX profile.

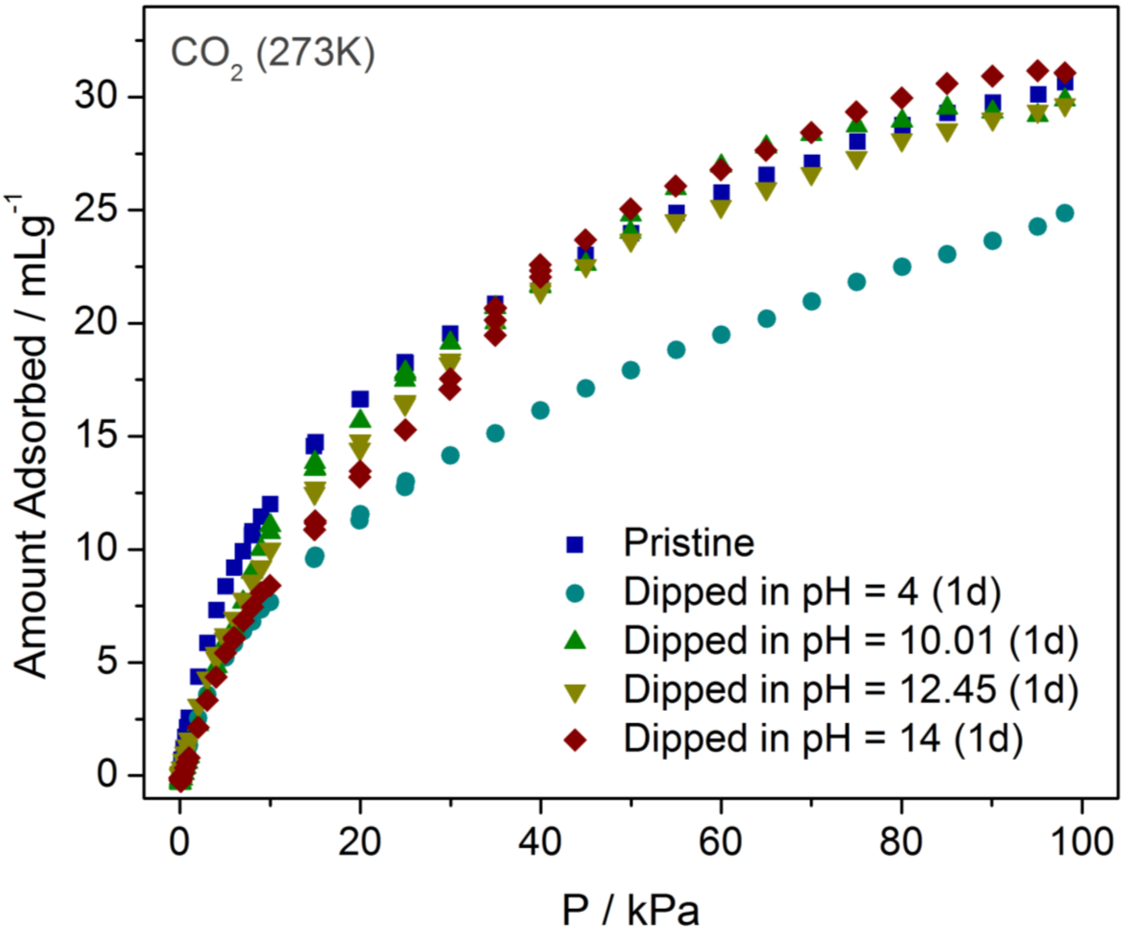

Figure S30: Stability Studies of IPM-MOF-201, related to Figure 2.
CO_2 adsorption isotherms at 273K for compound **IPM-MOF-201** dipped in different conditions.

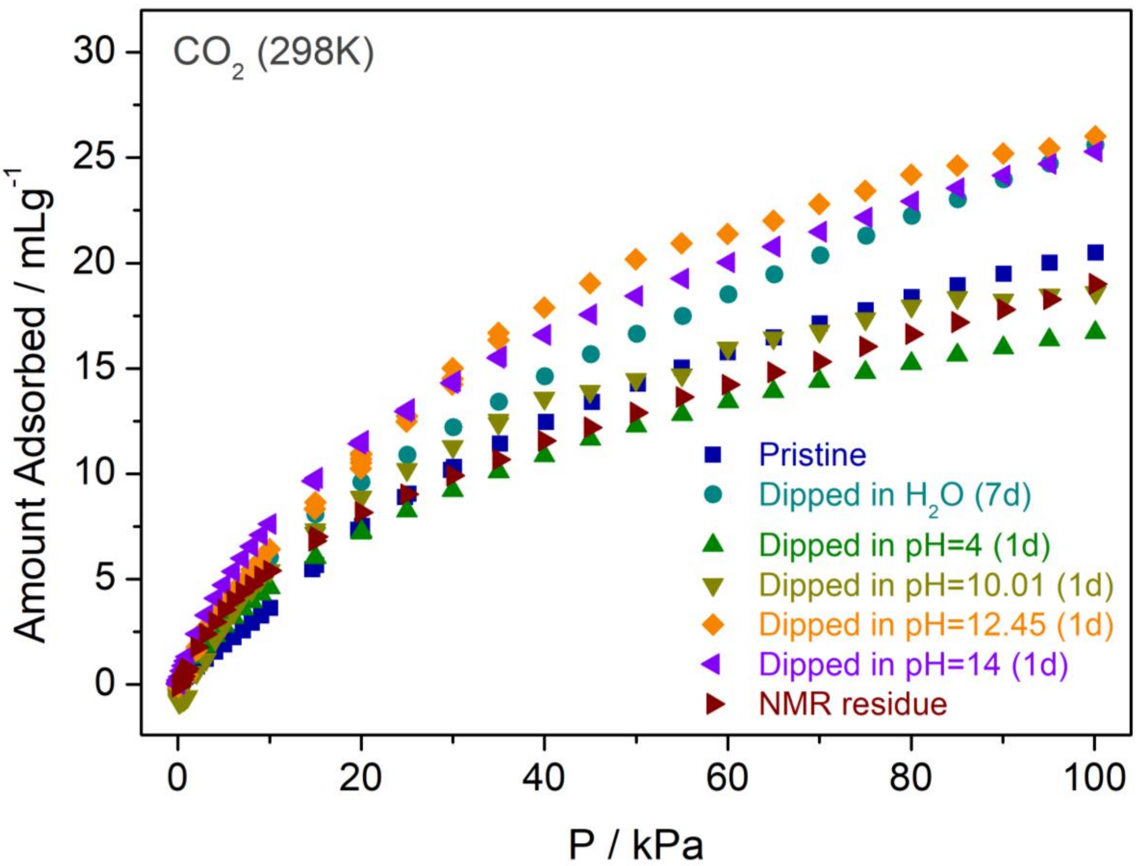

Figure S31: Stability Studies of IPM-MOF-201, related to Figure 2.
CO_2 adsorption isotherms at 298K for compound **IPM-MOF-201** dipped in different conditions.

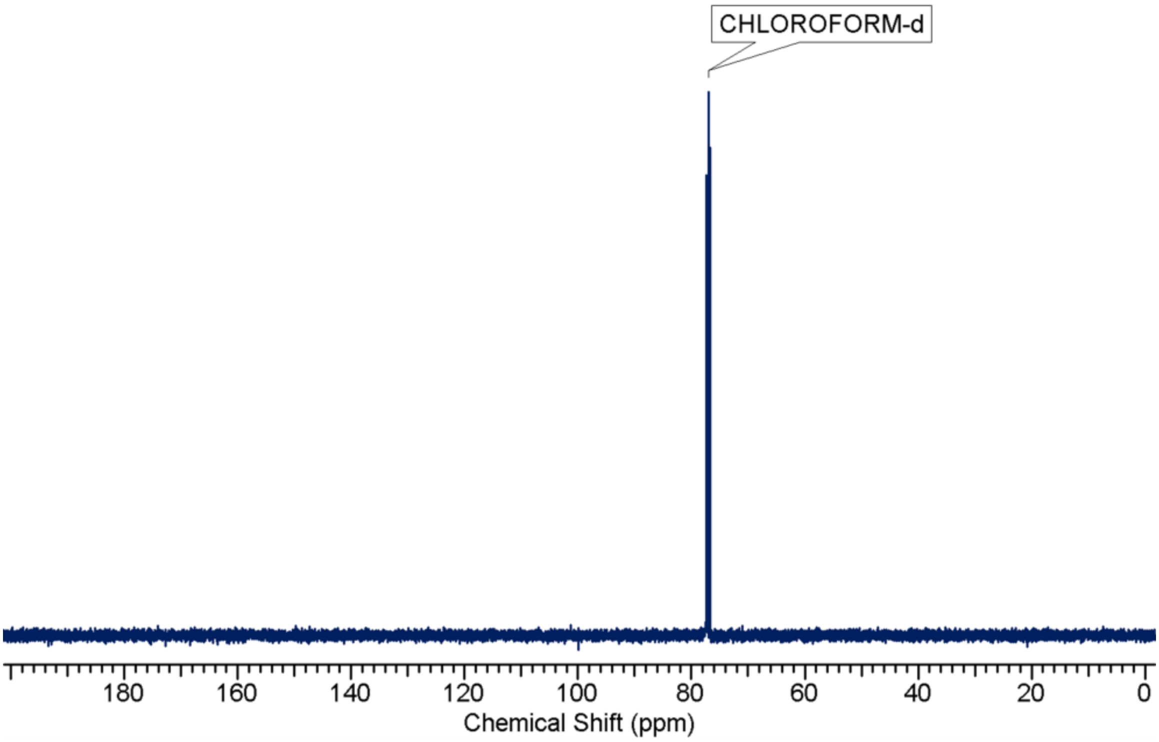

Figure S32: Stability Studies of IPM-MOF-201, related to Figure 2.
^{13}C-NMR of the supernatant obtained after treating **IPM-MOF-201** in solution of NaOD/D_2O for 1 day.

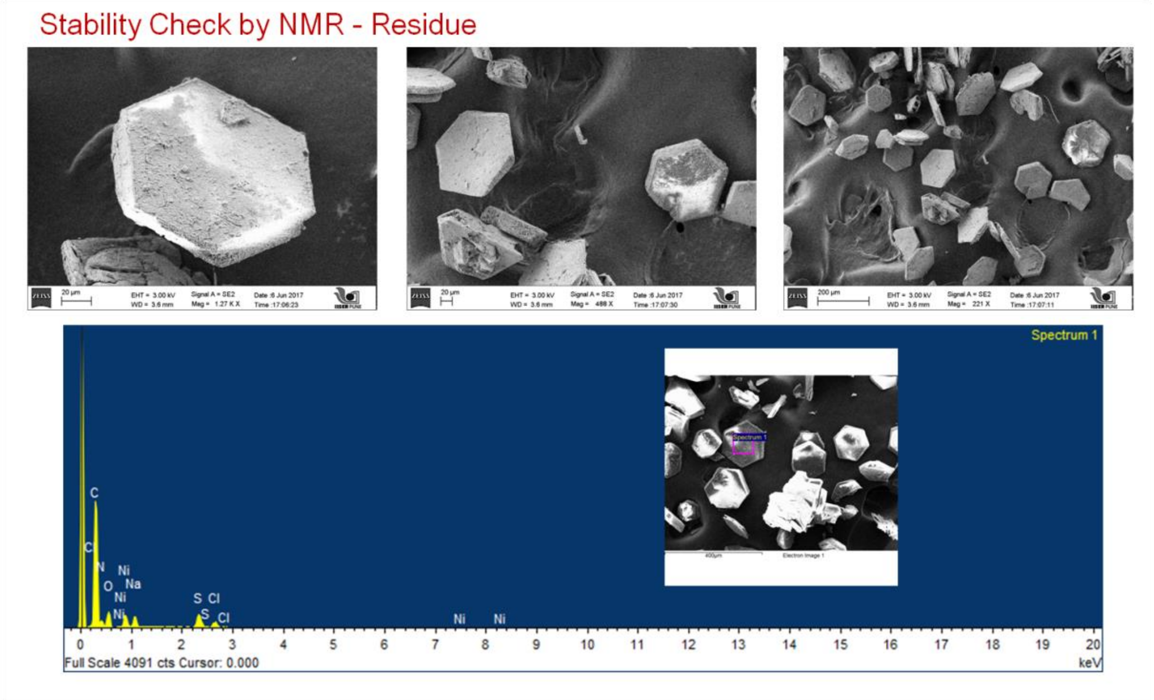

Figure S33: Stability Studies of IPM-MOF-201, related to Figure 2.
FESEM images of compound **IPM-MOF-201** & corresponding EDX profile after performing the NMR characterization experiment.

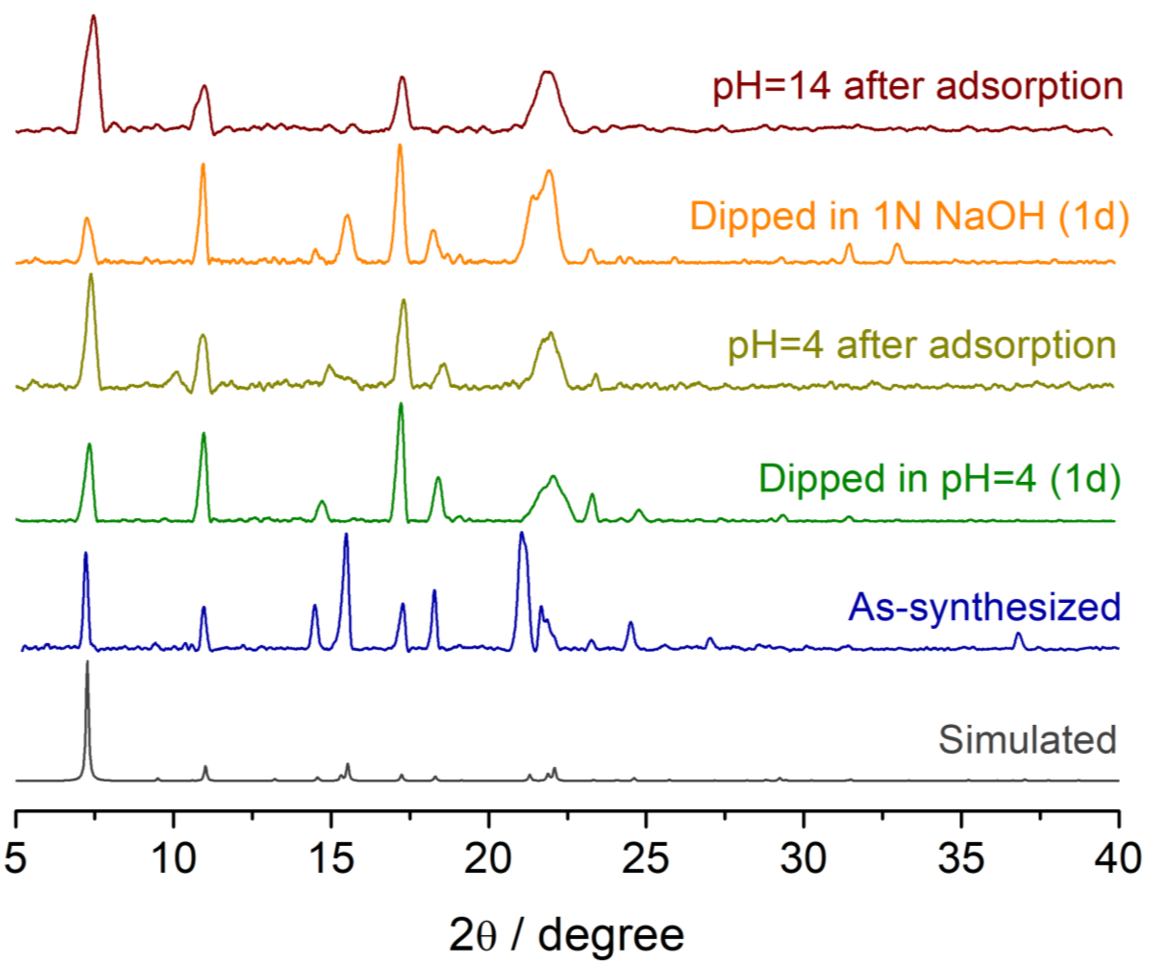

Figure S34: Stability Studies of IPM-MOF-201, related to Figure 2.
PXRD patterns for the pH dipped conditions and the post-adsorption phases.

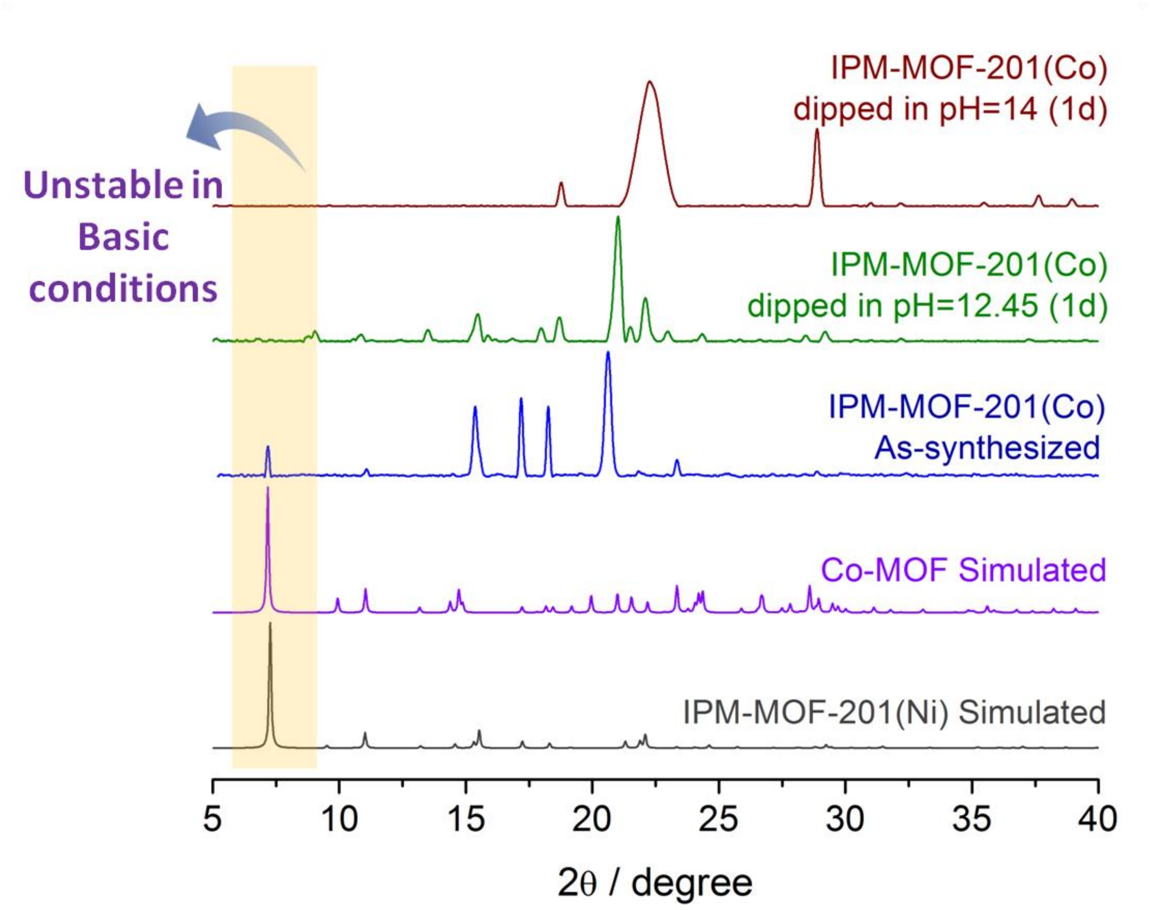

Figure S35: Stability Studies of IPM-MOF-201, related to Figure 2.
Powder diffraction patterns for compound **IPM-MOF-201(Ni)** (simulated, grey), Co-MOF (simulated, purple) [simulated pattern adapted from ref. (Yao et al., 2011)], as-synthesized **IPM-MOF-201(Co)** (blue) and phase dipped in pH=12.45 (green) and pH=14 (wine red).

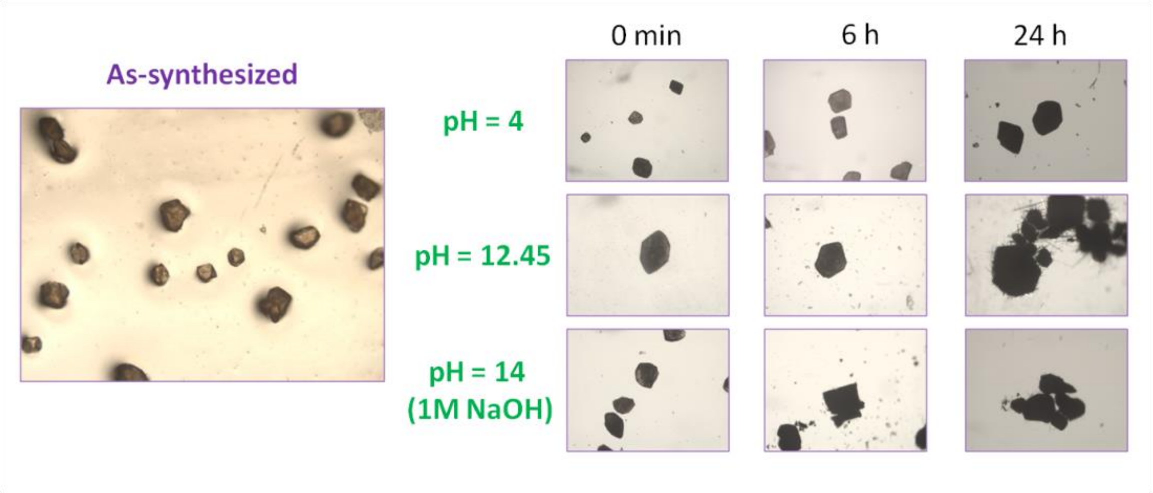

Figure S36: Stability Studies of IPM-MOF-201, related to Figure 2.
Photographs of Co-IPM-MOF when dipped in pH solutions of 4, 12.45 and 14 at different time intervals.

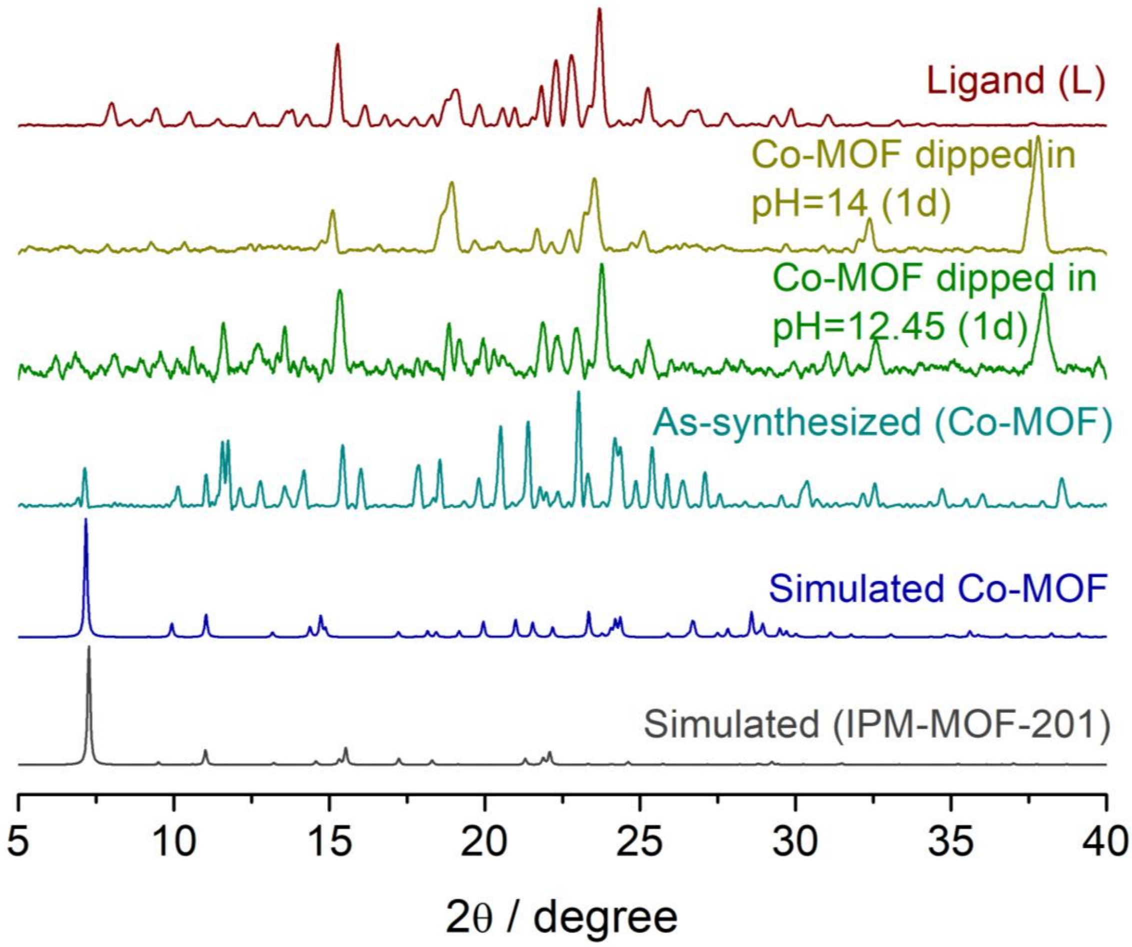

Figure S37: Stability Studies of IPM-MOF-201, related to Figure 2.
Powder diffraction patterns for compound **IPM-MOF-201(Ni)** (simulated, grey), Co-MOF (simulated, blue) [simulated pattern adapted from ref. (Yao et al., 2011)], as-synthesized Co-MOF (cyan), phase dipped in pH=12.45 (green) and pH=14 (yellow), and ligand (wine red).

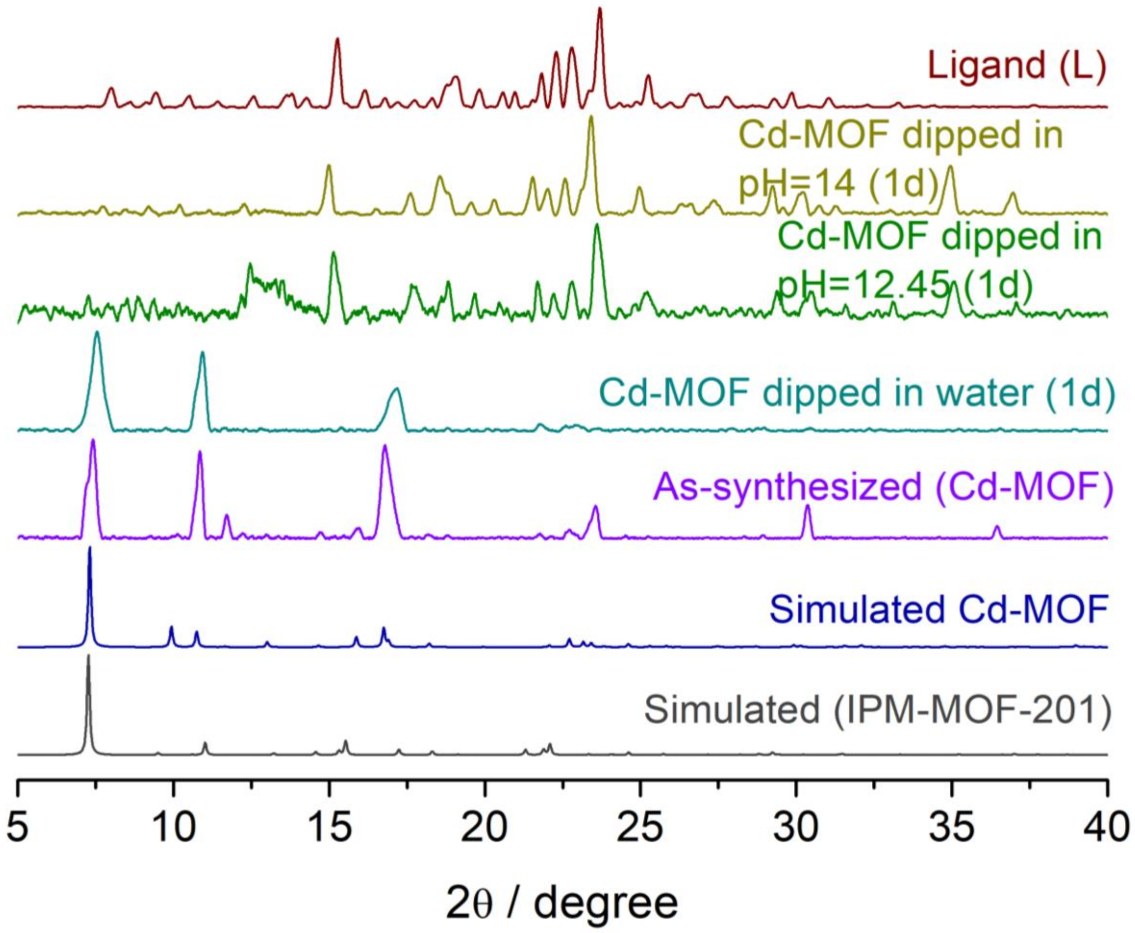

Figure S38: Stability Studies of IPM-MOF-201, related to Figure 2.
Powder diffraction patterns for compound **IPM-MOF-201(Ni)** (simulated, grey), Cd-MOF (simulated, blue) [simulated pattern adapted from ref. (Liu et al., 2014)], as-synthesized Cd-MOF (purple), dipped in water (cyan), phase dipped in pH=12.45 (green) and pH=14 (yellow), and ligand (wine red).

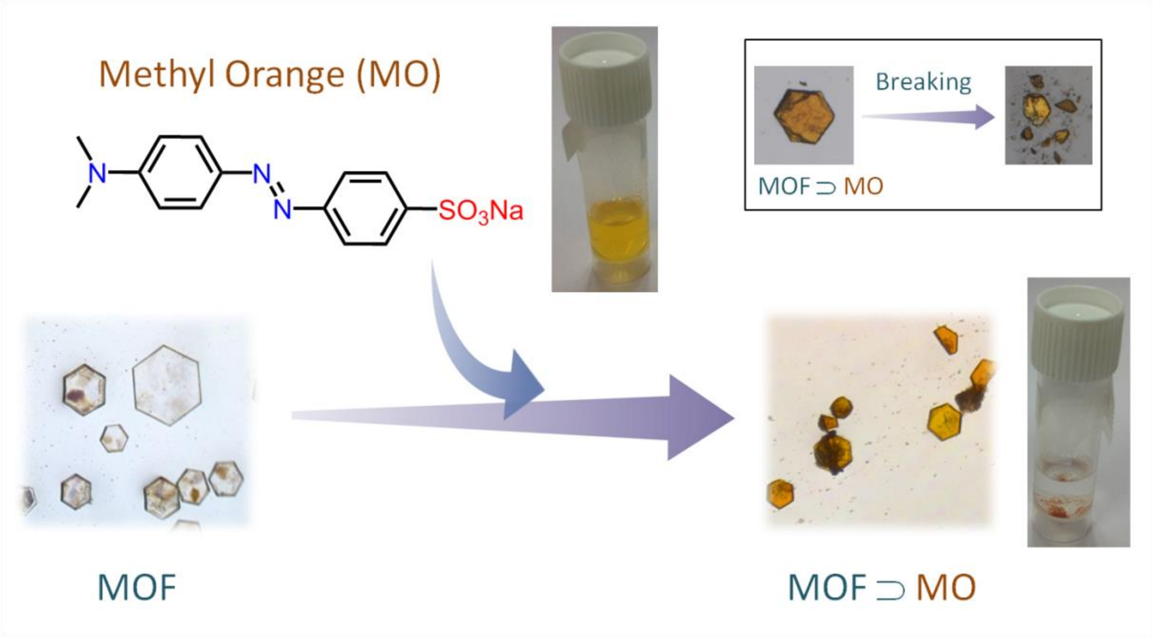

Figure S39: Dye capture studies by IPM-MOF-201, related to Figure 3.
Schematic illustration of dye inclusion (Methyl Orange - MO) in compound **IPM-MOF-201**. The corresponding photographs of the crystals under the microscope have been shown alongside. (Inset) The photograph of MO-exchanged crystals and the fragments after breaking it randomly.

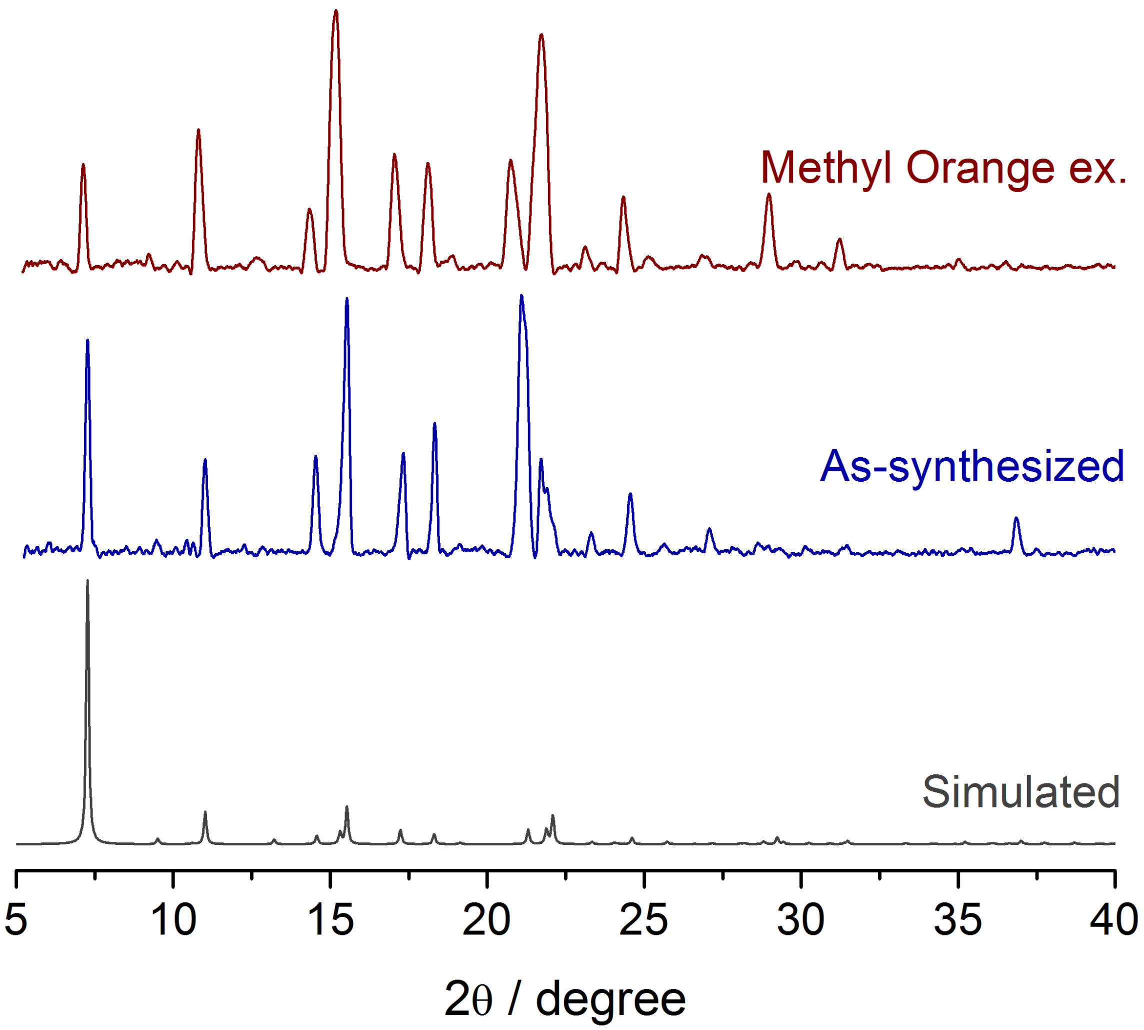

Figure S40: Dye capture studies by IPM-MOF-201, related to Figure 3.
Powder diffraction patterns for compound **IPM-MOF-201** (simulated, grey), as-synthesized (blue) and MO-exchange phase (wine red).

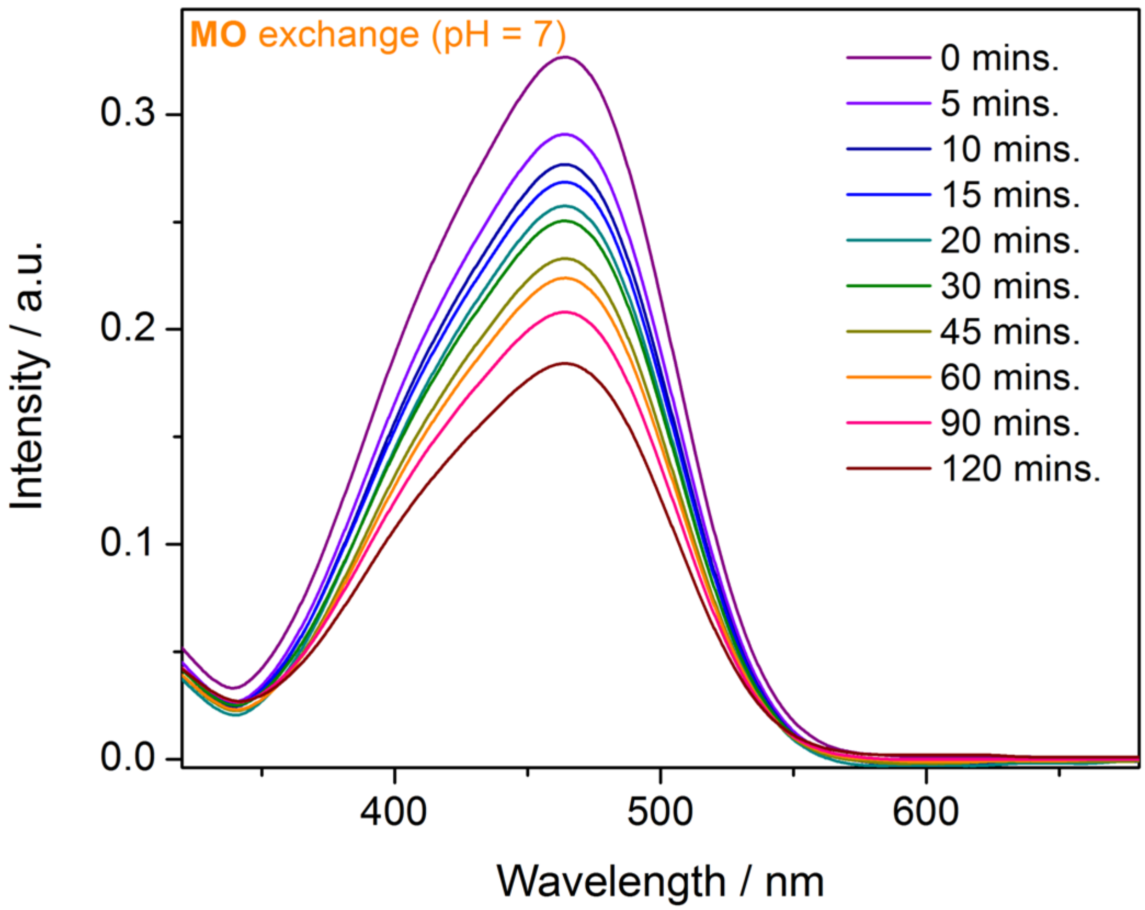

Figure S41: Dye capture studies by IPM-MOF-201, related to Figure 3.
UV-Vis spectra of the supernatant aqueous MO solution after addition of compound **IPM-MOF-201** at different time intervals.

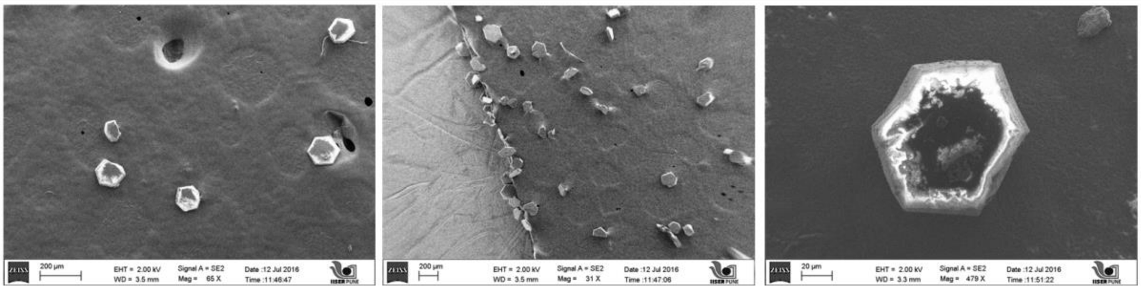

Figure S42: Dye capture studies by IPM-MOF-201, related to Figure 3.
FESEM images of compound **IPM-MOF-201** dipped in aqueous MO solution.

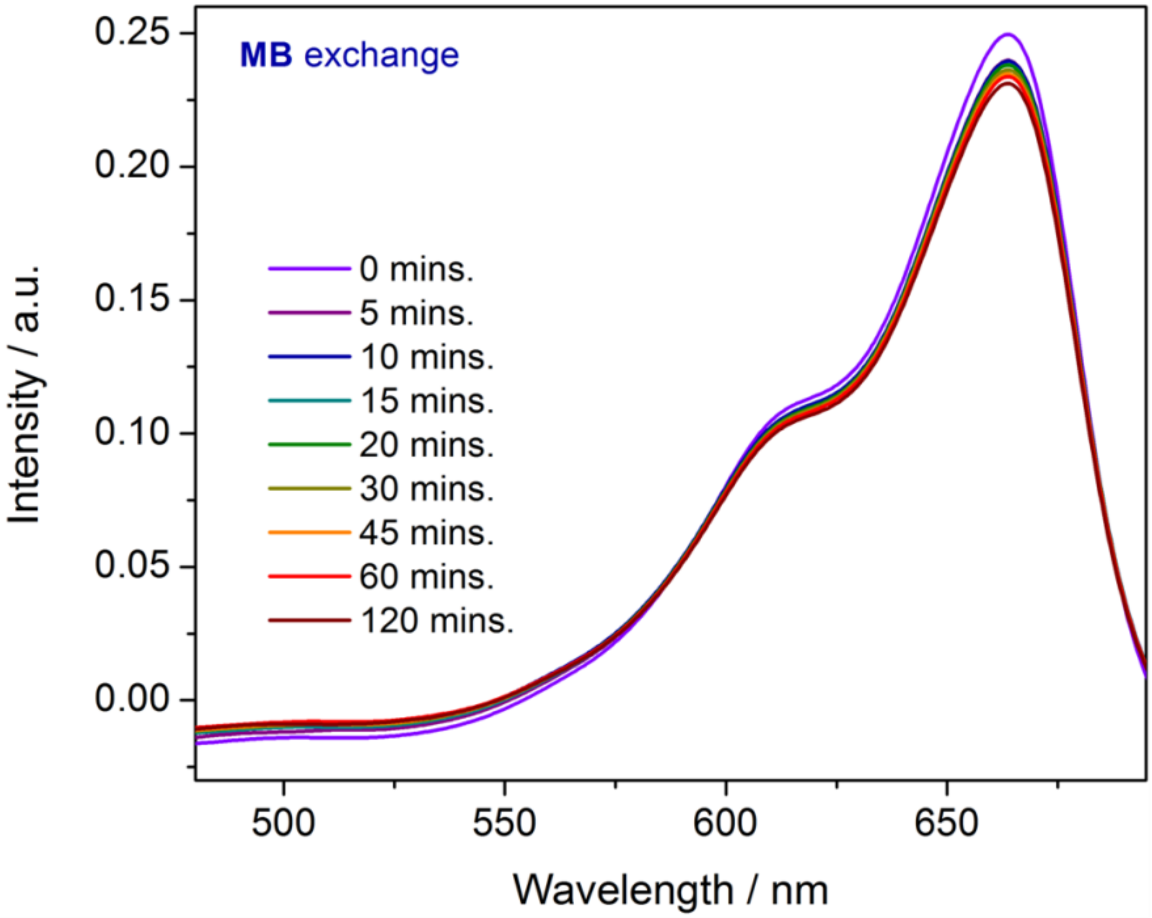

Figure S43: Dye capture studies by IPM-MOF-201, related to Figure 3.
UV-Vis spectra of the supernatant aqueous MB solution after addition of compound **IPM-MOF-201** at different time intervals.

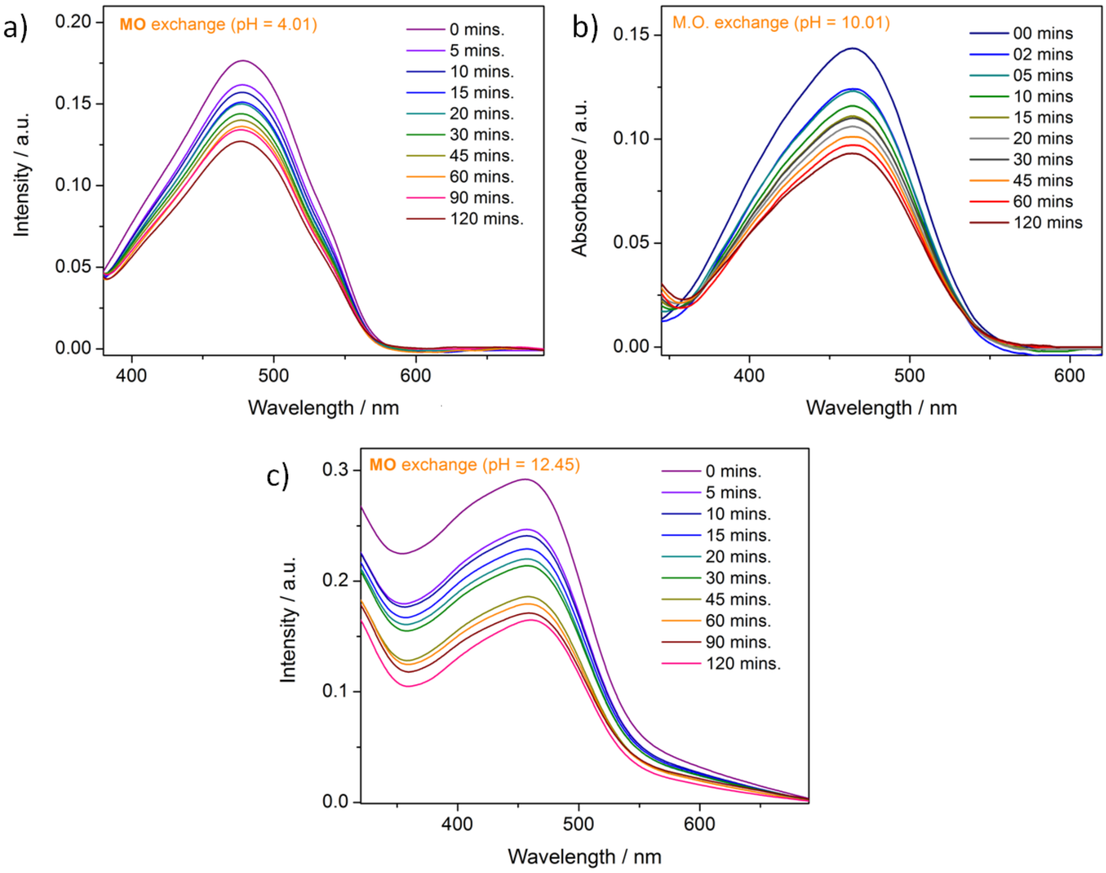

Figure S44: Dye capture studies by IPM-MOF-201, related to Figure 3.
UV-Vis spectra of the supernatant MO solution having pH a) 4.01, b) 10.01 and c) 12.45, after addition of compound **IPM-MOF-201** to respective phases at different time intervals.

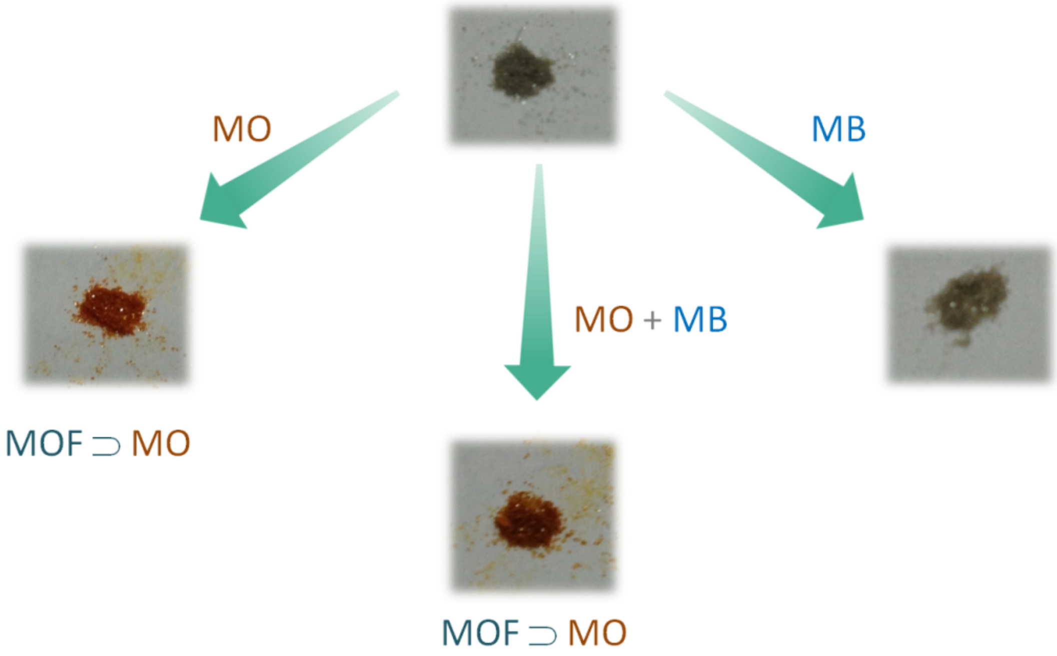

Figure S45: Dye capture studies by IPM-MOF-201, related to Figure 3.
Photographs showing preferential capture of anionic dye over cationic dye, even in presence of equimolar mixture of both dyes.

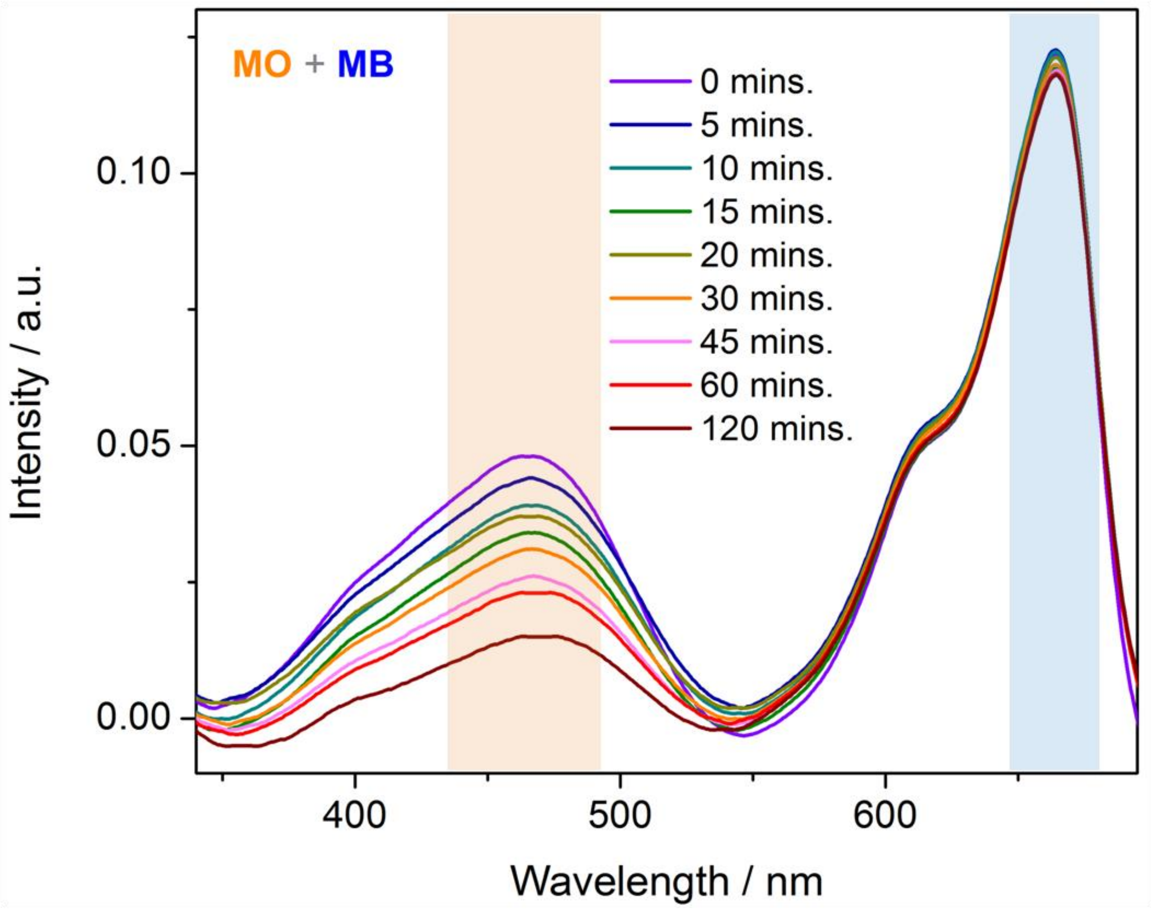

Figure S46: Dye capture studies by IPM-MOF-201, related to Figure 3.
UV-Vis spectra of the supernatant solution of equimolar mixture of MB & MO after addition of compound **IPM-MOF-201** recorded at different time intervals.

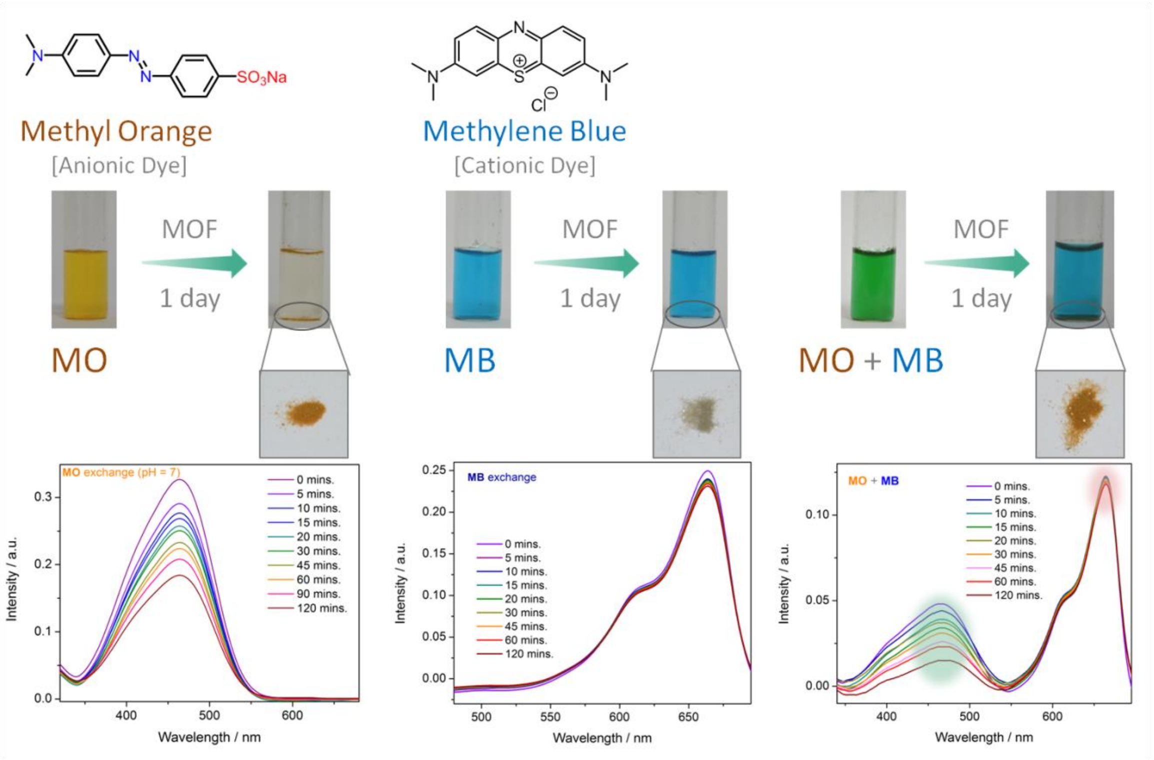

Figure S47: Dye capture studies by IPM-MOF-201, related to Figure 3.
Overall representation of the charge selective dye capture by **IPM-MOF-201**.

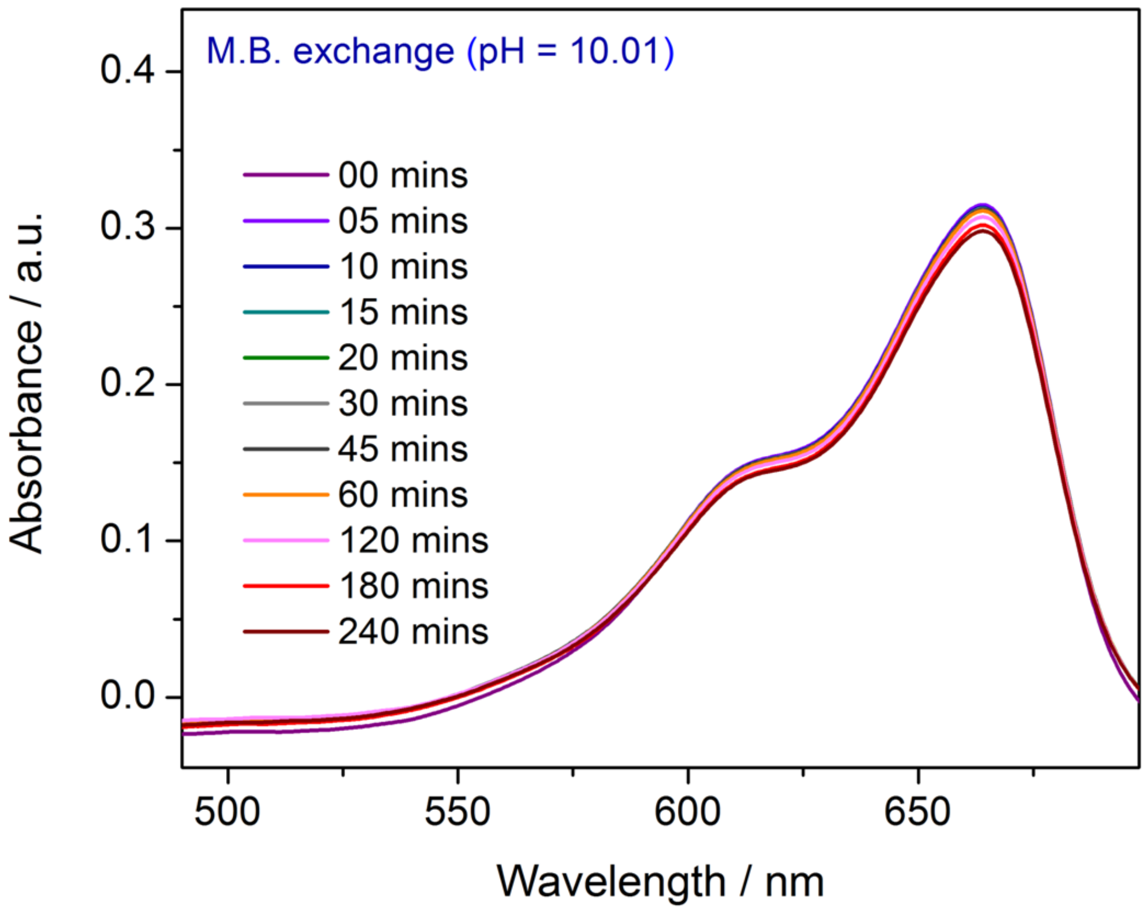

Figure S48: Dye capture studies by IPM-MOF-201, related to Figure 3.
UV-Vis spectra of the supernatant MB solution of pH = 10.01 after addition of compound **IPM-MOF-201** recorded at different time intervals.

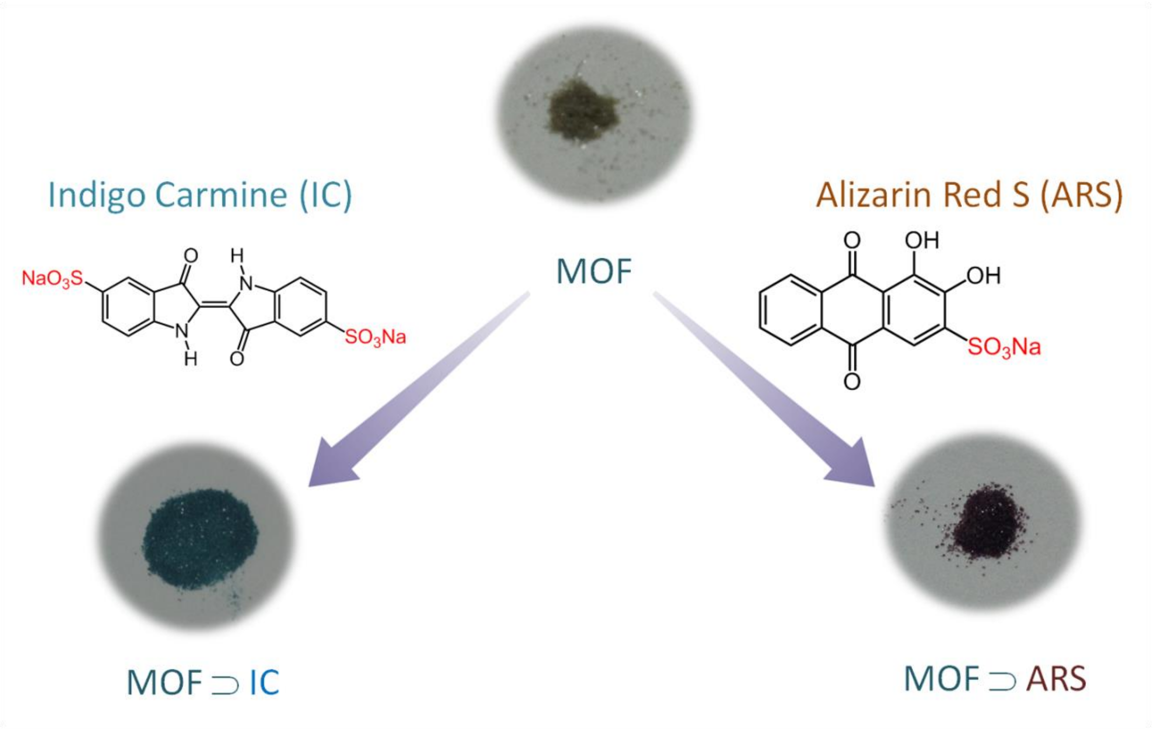

Figure S49: Dye capture studies by IPM-MOF-201, related to Figure 3.
Illustration showing capture of anionic dyes (IC & ARS) by compound **IPM-MOF-201**.

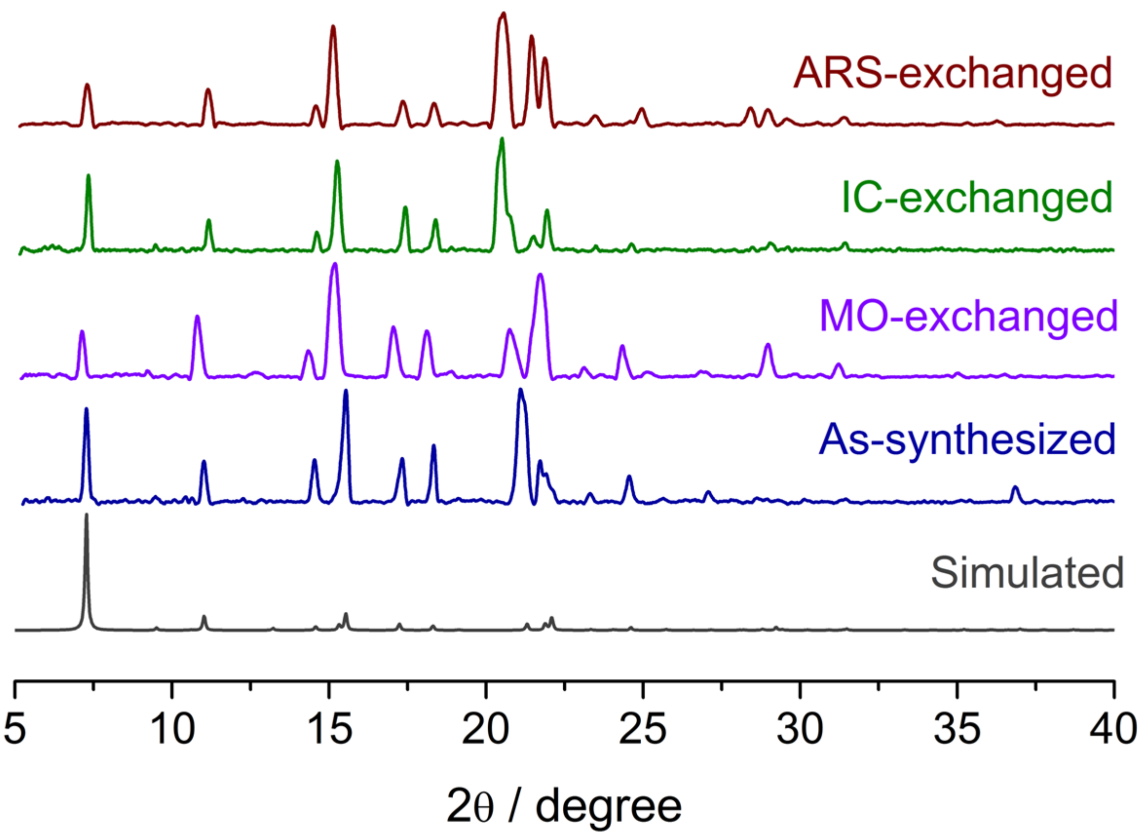

Figure S50: Dye capture studies by IPM-MOF-201, related to Figure 3.
Powder x-ray diffraction patterns of simulated (grey), as-synthesized (blue), and aqueous phase dye exchanged phases (MO - purple, IC - green, ARS - wine red).

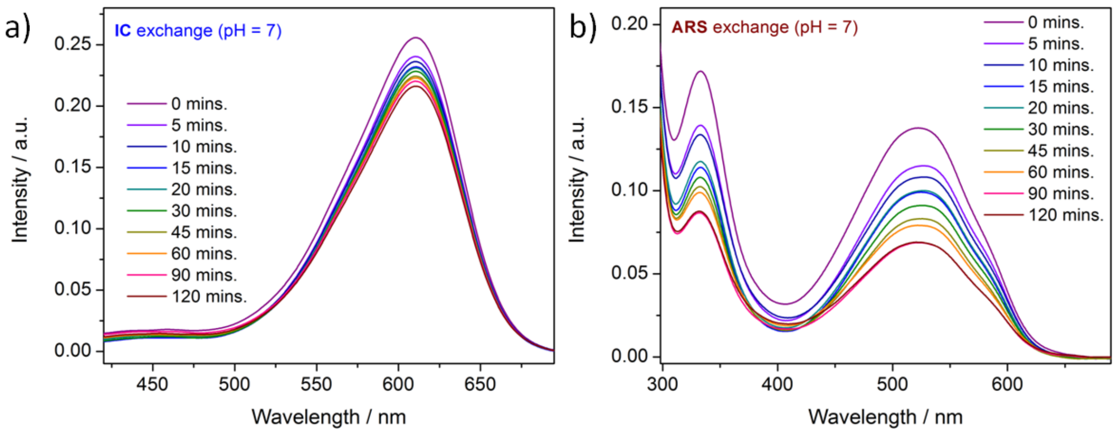

Figure S51: Dye capture studies by IPM-MOF-201, related to Figure 3.
UV-Vis spectra of the supernatant aqueous solution of a) IC and b) ARS, after addition of compound **IPM-MOF-201** at different time intervals.

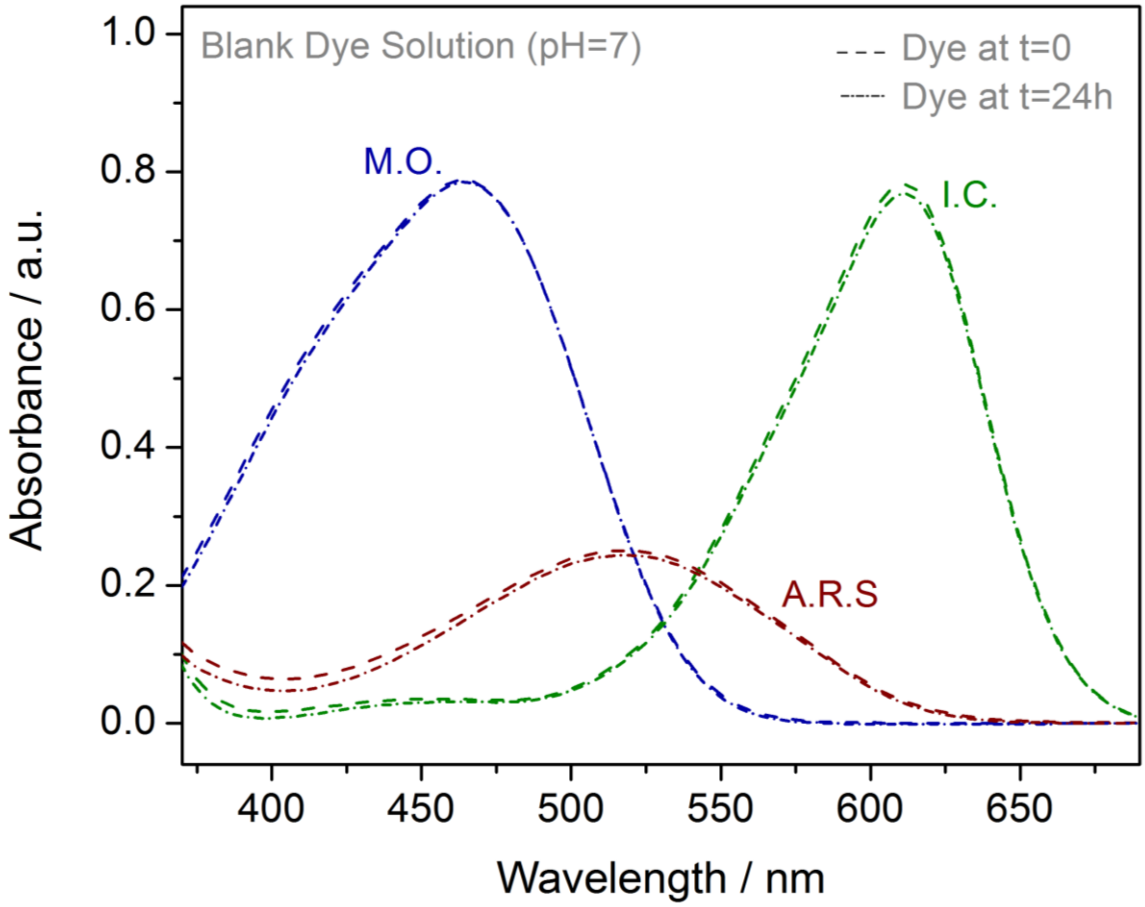

Figure S52: Dye capture studies by IPM-MOF-201, related to Figure 3.
UV-Vis spectra of blank aqueous dye solutions at different time intervals.

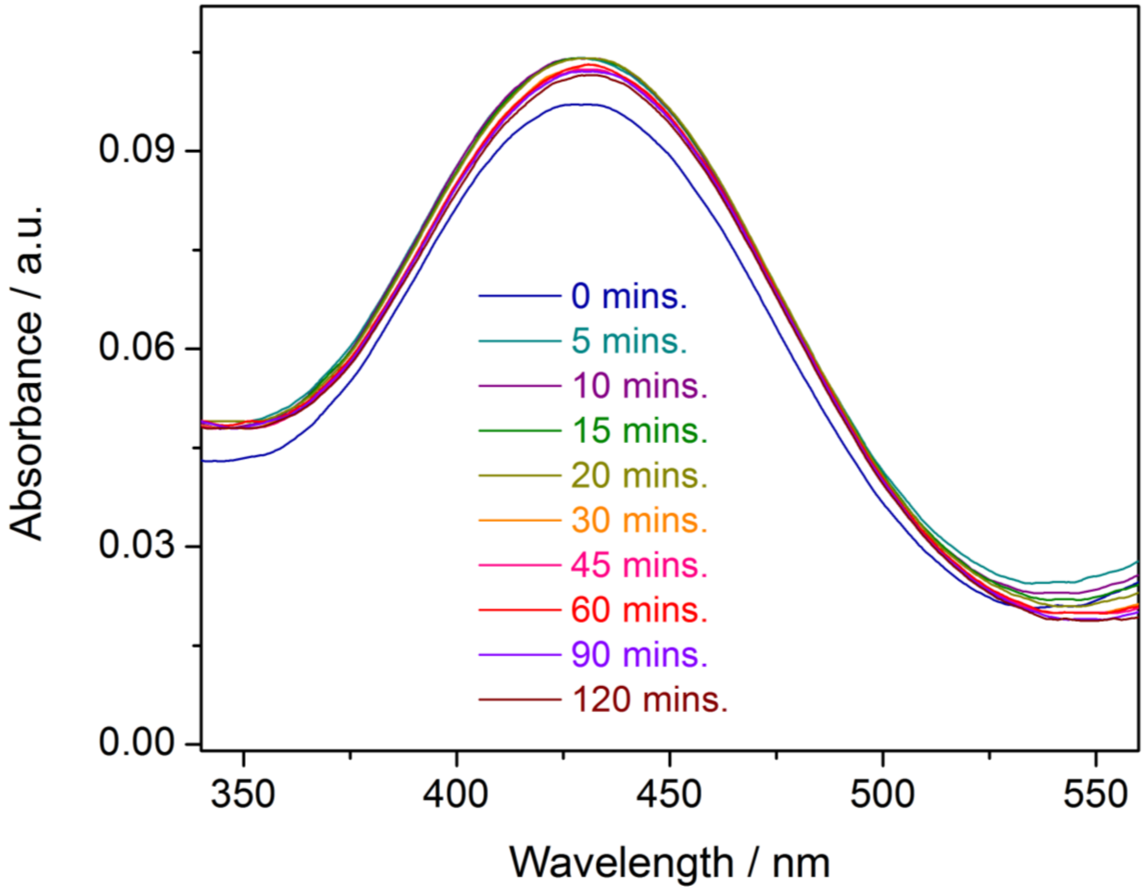

Figure S53: Dye capture studies by IPM-MOF-201, related to Figure 3.
UV-Vis spectra of the supernatant aqueous solution of bromothymol blue (BTB) at pH=7 after addition of compound **IPM-MOF-201** at different time intervals.

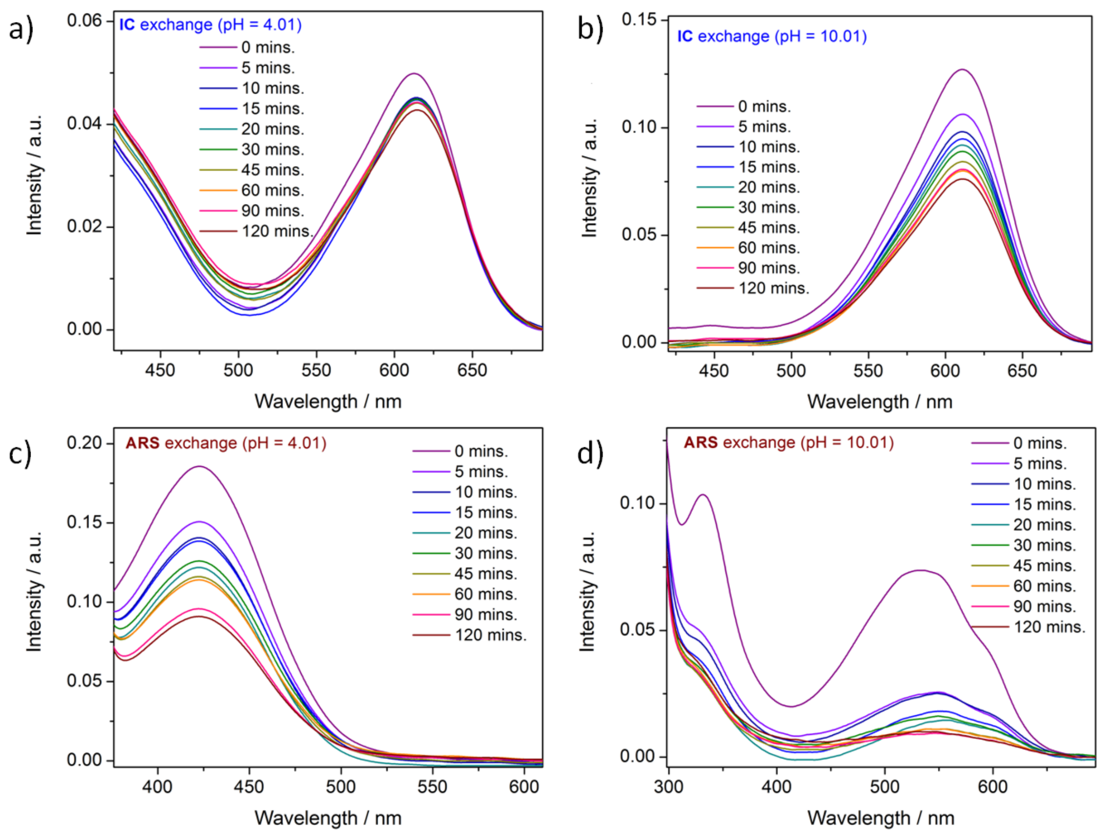

Figure S54: Dye capture studies by IPM-MOF-201, related to Figure 3.
UV-Vis spectra of the supernatant solution of a) IC (pH=4.01), b) IC (pH =10.01), c) ARS (pH=4.01) and d) ARS (pH=10.01), after addition of compound **IPM-MOF-201** to respective phases at different time intervals.

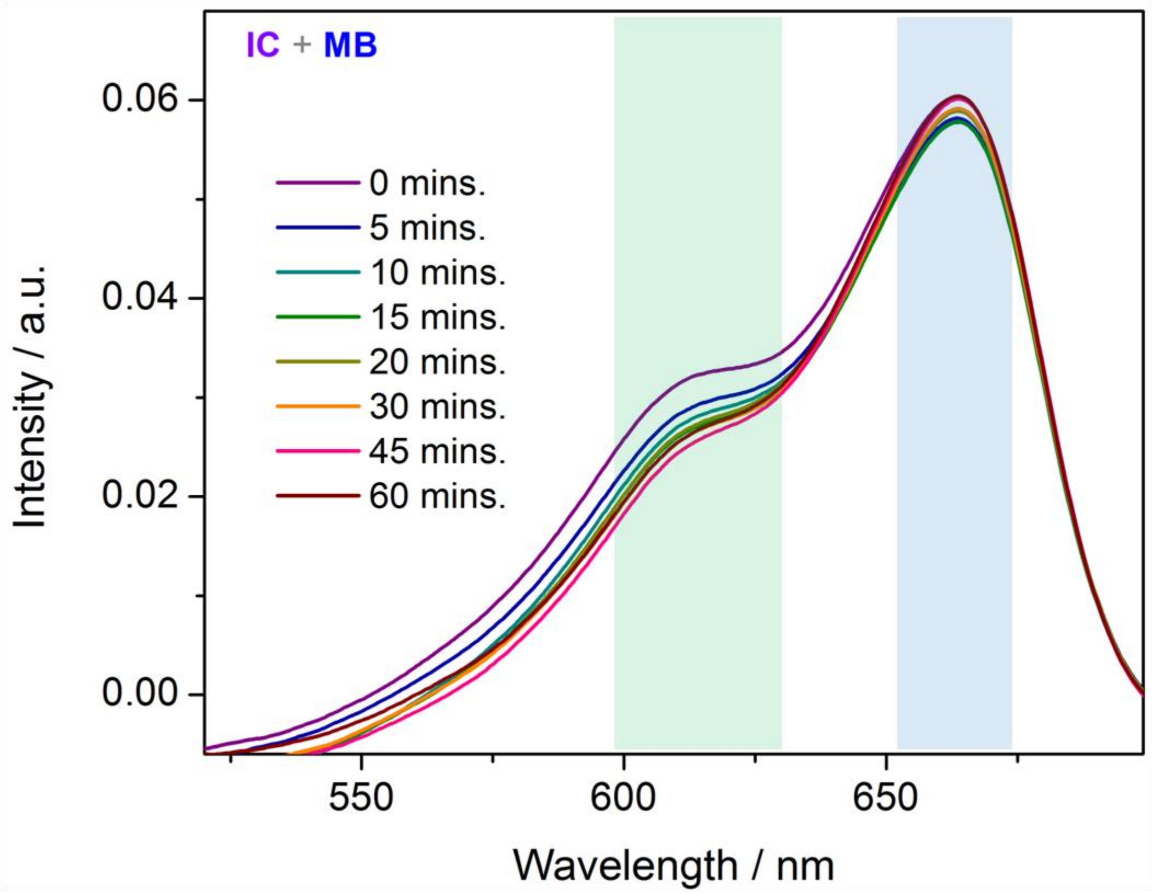

Figure S55: Dye capture studies by IPM-MOF-201, related to Figure 3.
UV-Vis spectra of the supernatant solution of equimolar mixture of MB & IC after addition of compound **IPM-MOF-201** recorded at different time intervals.

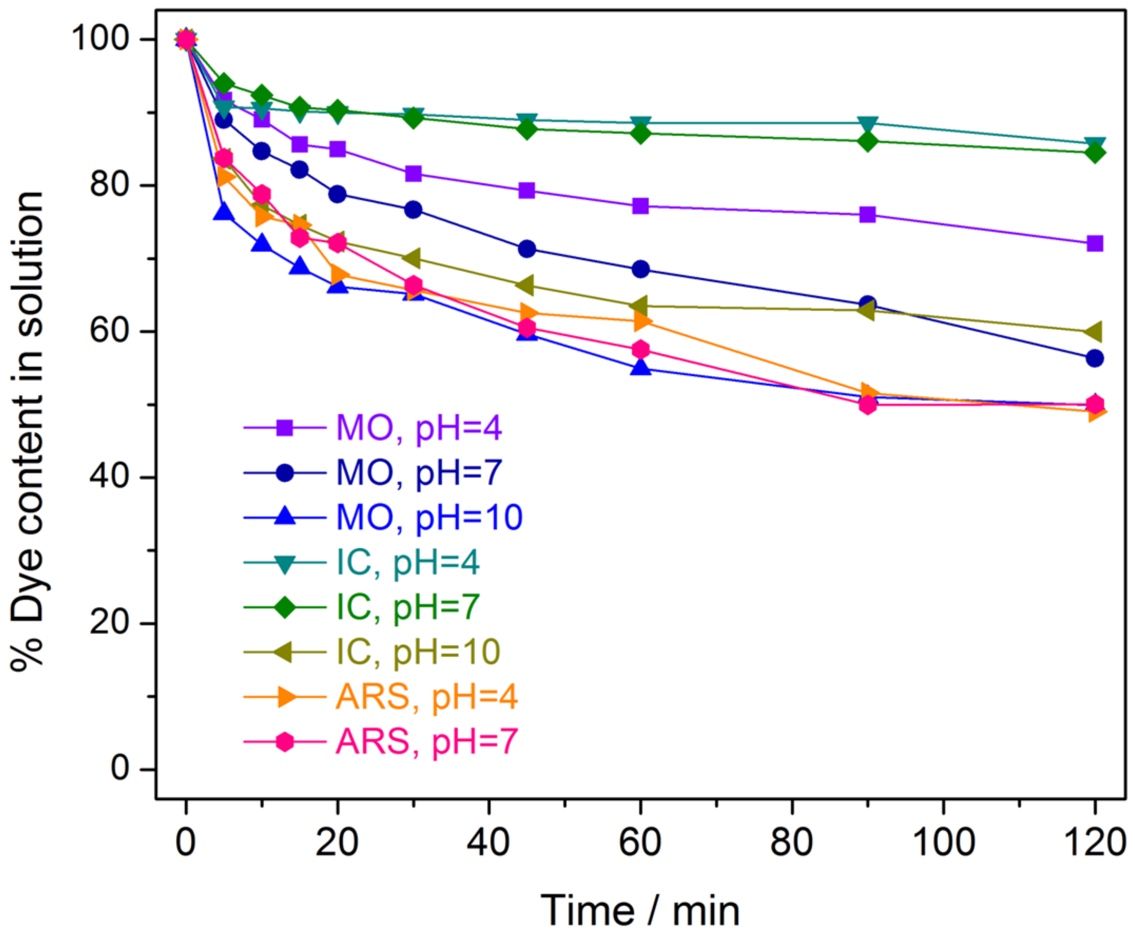

Figure S56: Dye capture studies by IPM-MOF-201, related to Figure 3.
Plot showing the rate of decrement in the concentration of the supernatant solution upon addition of compound **IPM-MOF-201**, recorded at different time intervals.

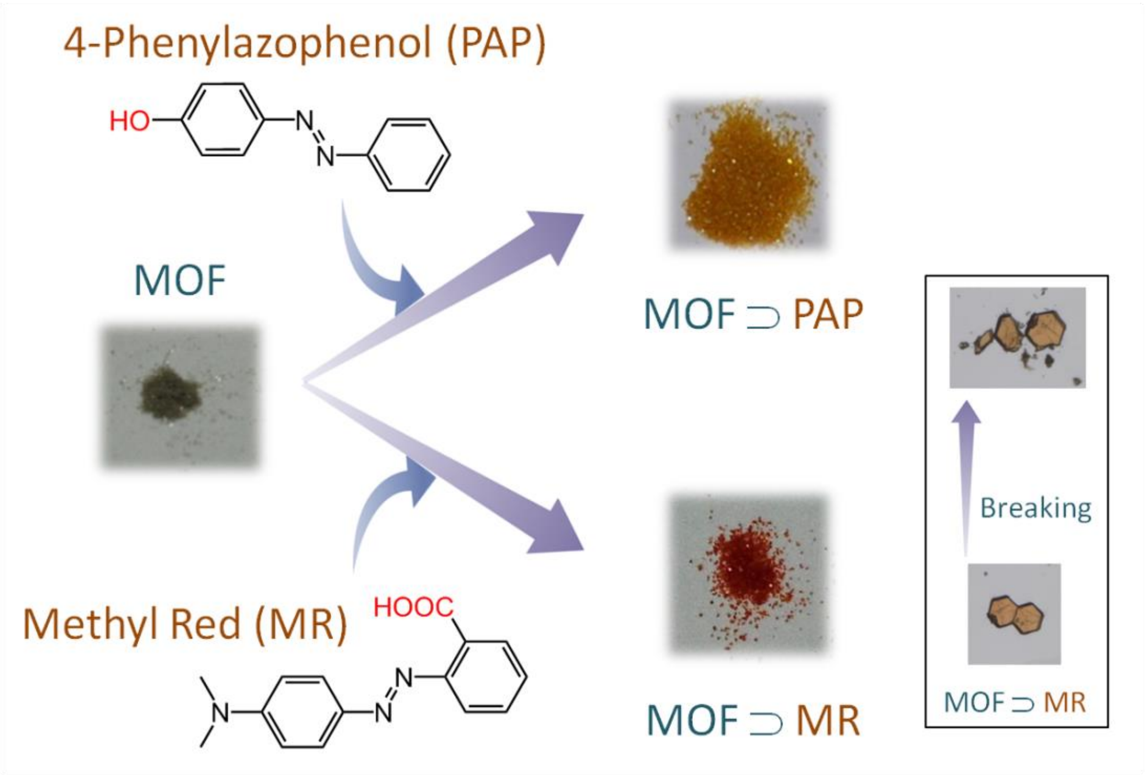

Figure S57: Dye capture studies by IPM-MOF-201, related to Figure 3.
Illustration showing capture of anionic dyes (PAP & MR) by compound **IPM-MOF-201**. (Inset): The photograph of MR-exchanged crystals and the fragments after breaking it randomly.

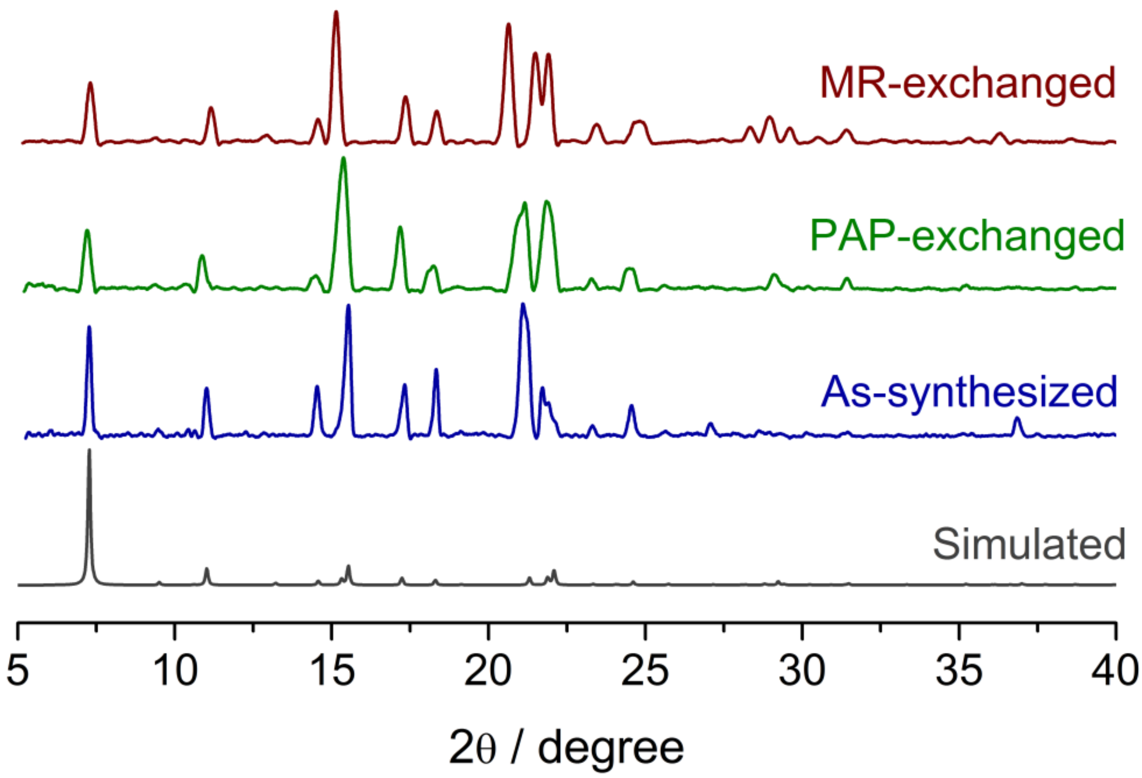

Figure S58: Dye capture studies by IPM-MOF-201, related to Figure 3.
Powder x-ray diffraction patterns of simulated (grey), as-synthesized (blue), and aqueous phase dye exchanged phases (PAP - green, MR - wine red).

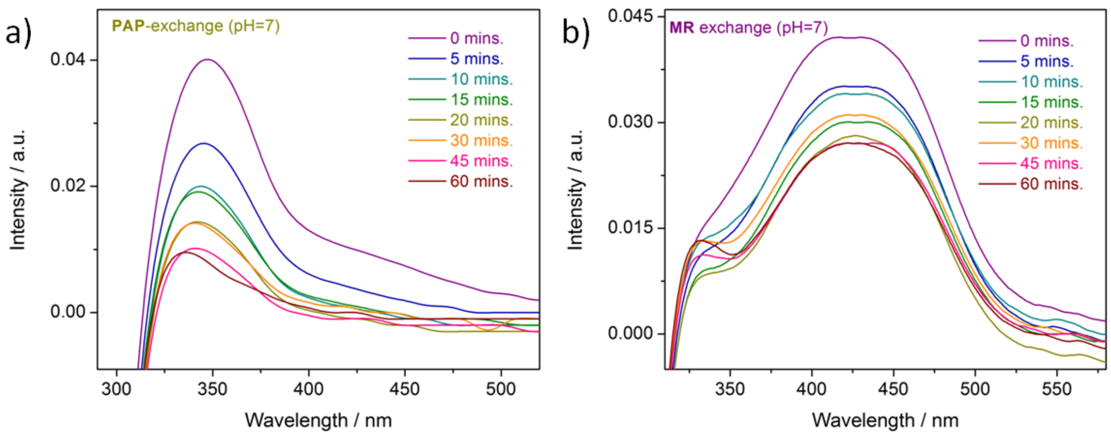

Figure S59: Dye capture studies by IPM-MOF-201, related to Figure 3.
UV-Vis spectra of the supernatant aqueous solution of a) PAP and b) MR, after addition of compound **IPM-MOF-201** at different time intervals.

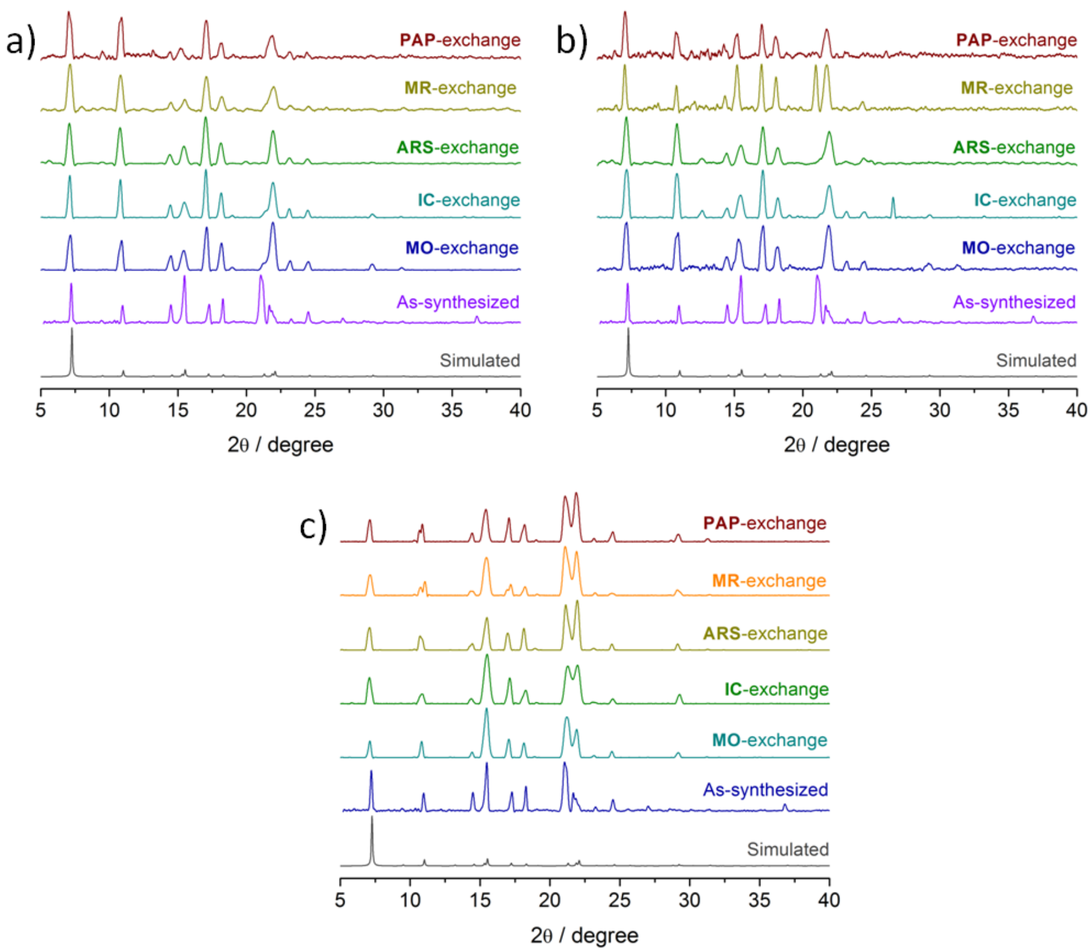

Figure S60: Dye capture studies by IPM-MOF-201, related to Figure 3.
Powder x-ray diffraction patterns of dye-exchanged phases at different pH, a) pH = 4.01, b) pH = 10.01 and c) pH = 12.45.

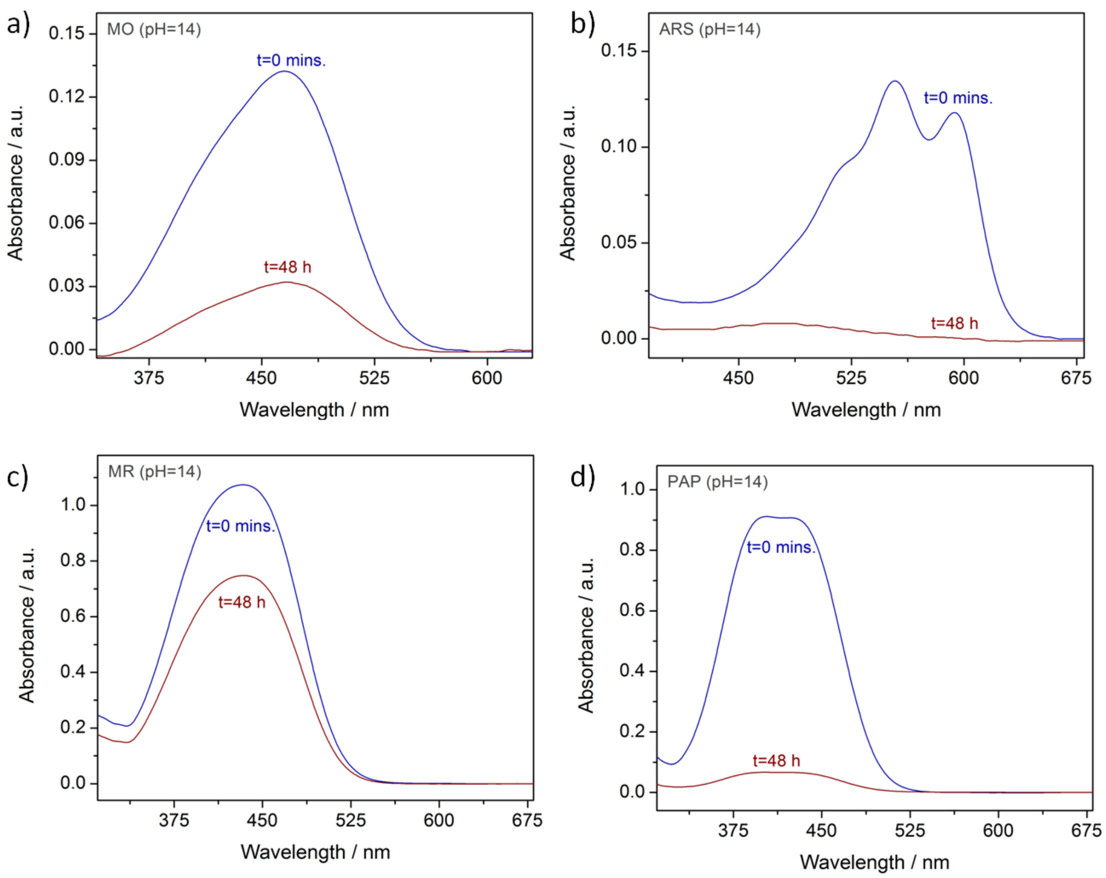

Figure S61: Dye capture studies by IPM-MOF-201, related to Figure 3.
UV-vis spectra of the supernatant at pH = 14, after addition of compound **IPM-MOF-201** at two time intervals.

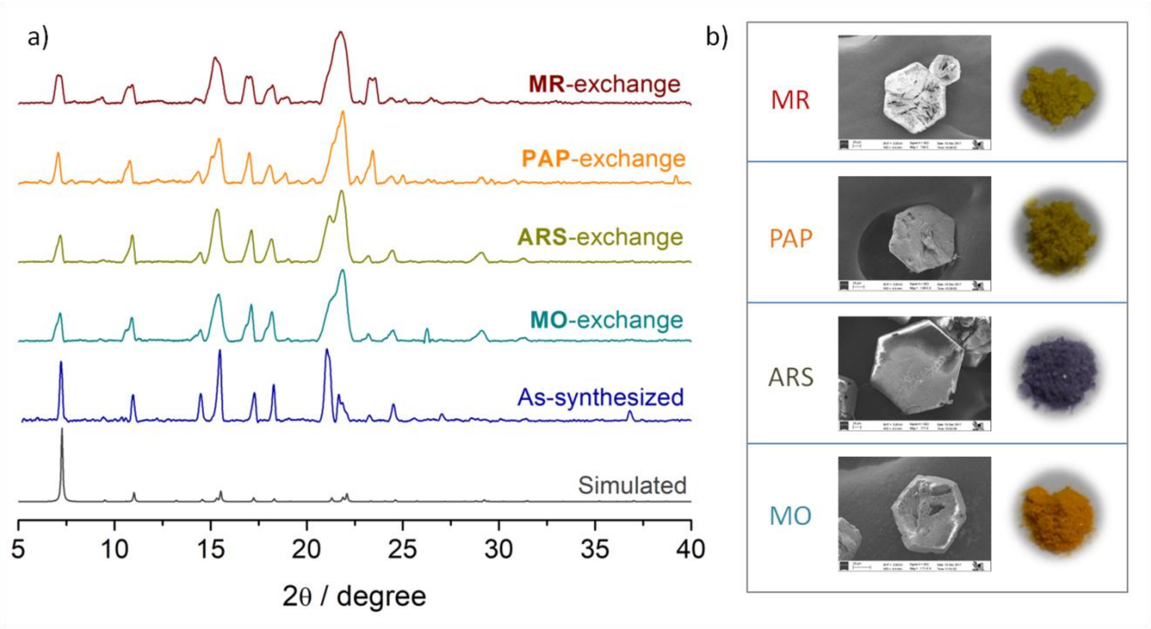

Figure S62: Dye capture studies by IPM-MOF-201, related to Figure 3.
a) Powder x-ray diffraction patterns of dye-exchanged phases at pH = 14, b) corresponding SEM images and naked-eye photographs.

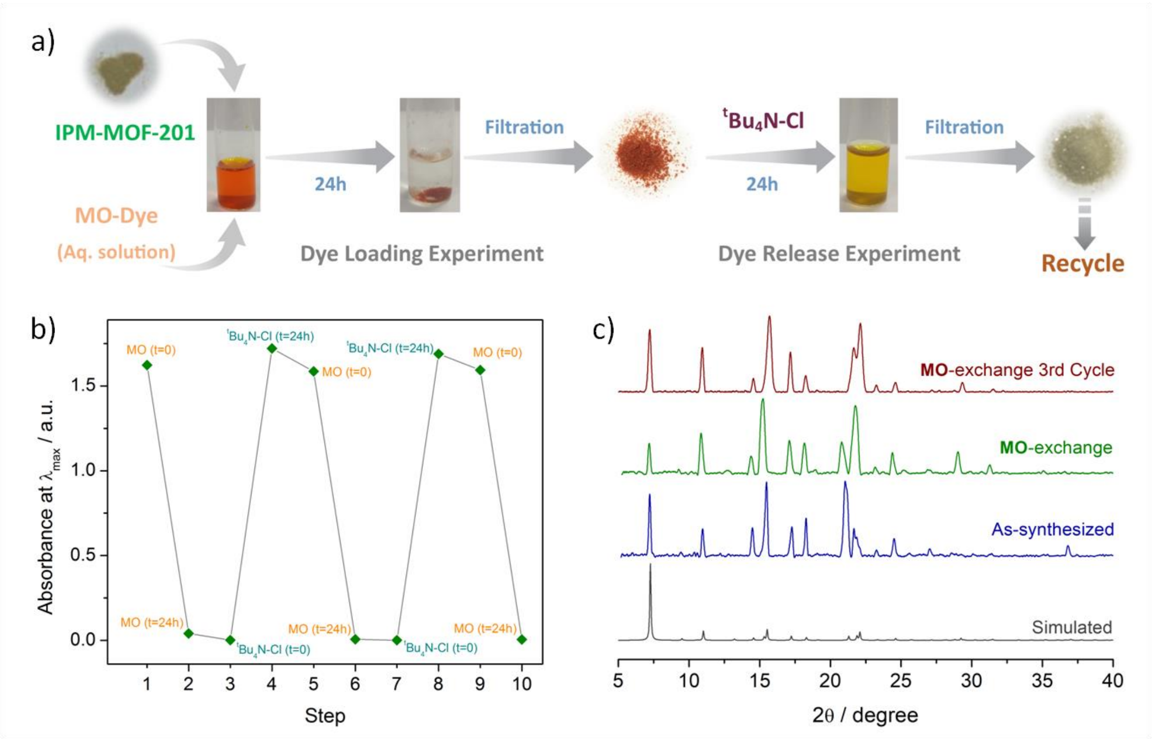

Figure S63: Dye capture studies by IPM-MOF-201, related to Figure 3.

a) Schematic depiction of the cyclic experiment for capture of MO-dye, b) absorbance of the supernatant during the adsorption-desorption cycles; c) PXRD patterns of the phase recovered after the 3rd adsorption cycle.

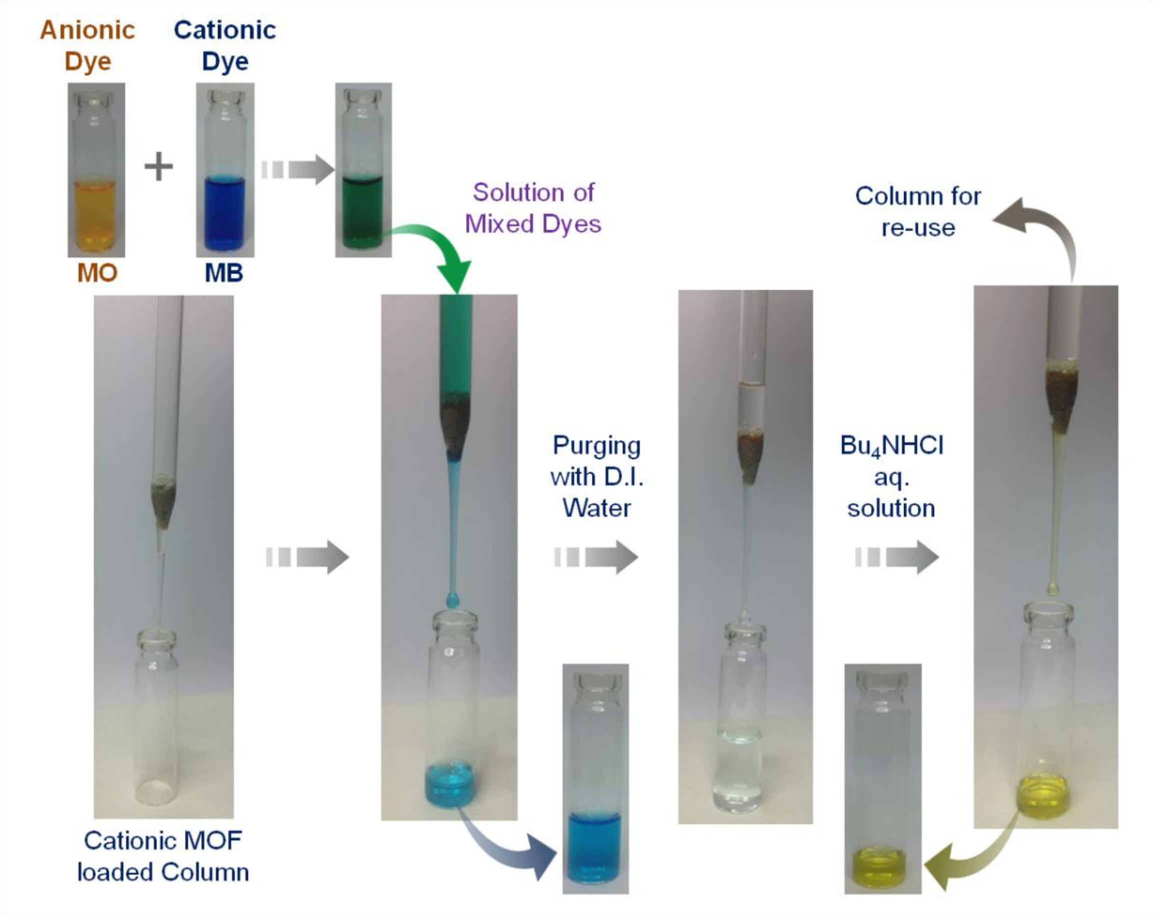

Figure S64: Dye capture studies by IPM-MOF-201, related to Figure 3.
Representation of the selective dye capture process by a MOF-loaded column.

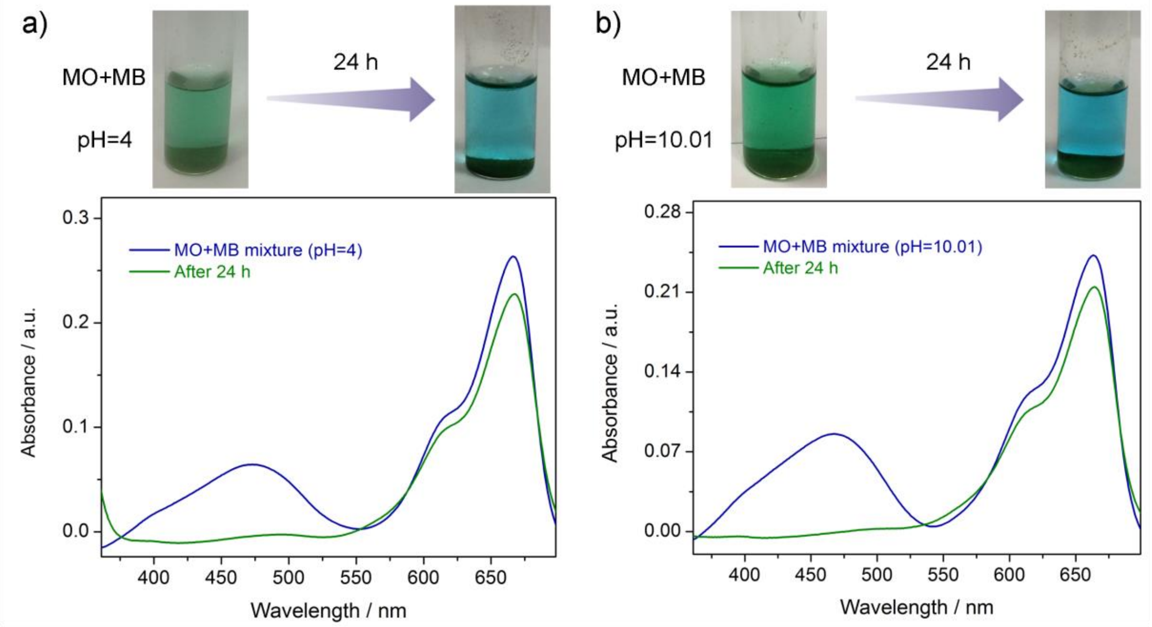

Figure S65: Dye capture studies by IPM-MOF-201, related to Figure 3.
Naked-eye changes and corresponding UV-Vis spectral profiles for mixtures of methyl orange (MO) [anionic] and methylene blue [cationic] at different time intervals for a) pH=4 and b) pH=10.01.

SCHEMES

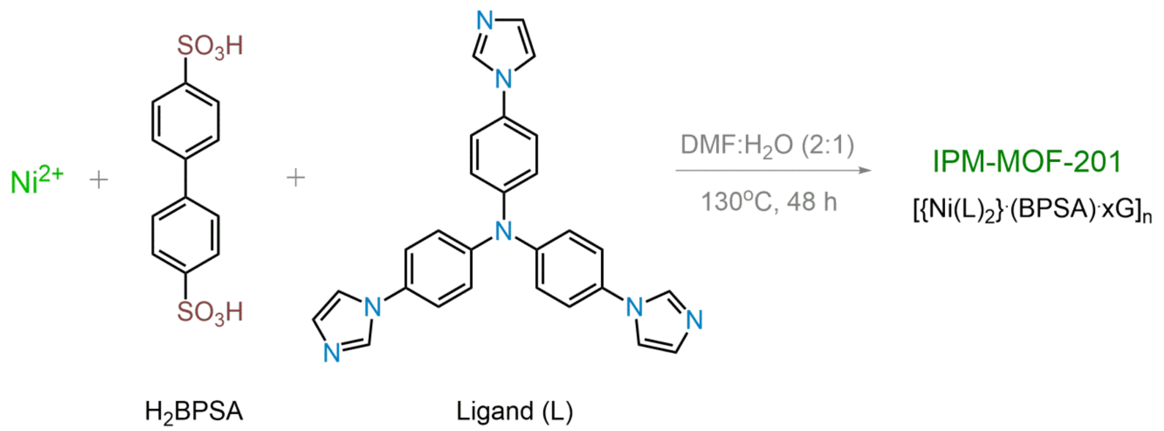

Scheme S1: Representation of the protocol employed for the synthesis of **IPM-MOF-201**, related to Figure 1.

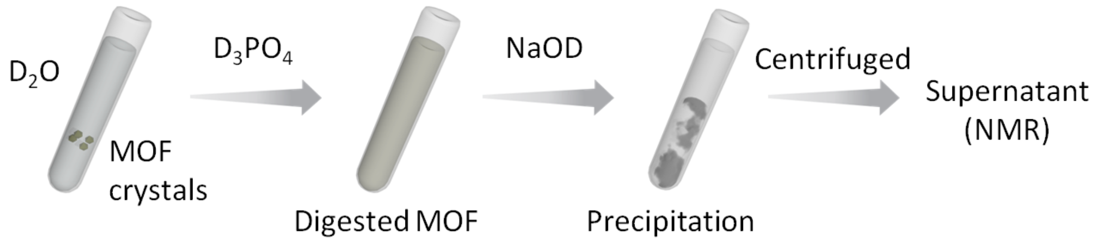

Scheme S2: Schematic illustration of the protocol employed for digesting **IPM-MOF-201**, related to Figure 1.

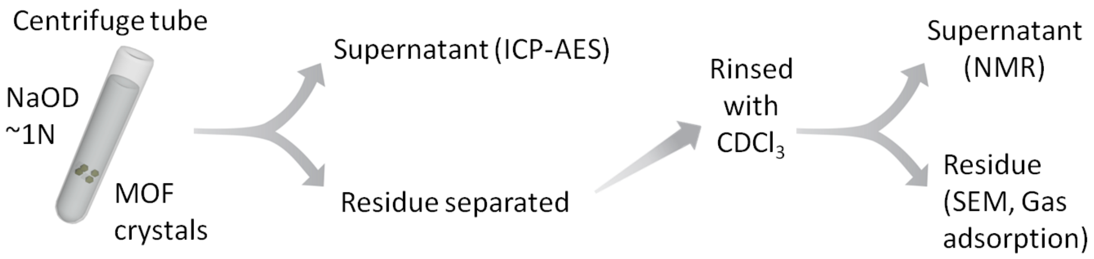

Scheme S3: Schematic illustration of the protocol employed for the ascertaining the base stability of **IPM-MOF-201**, related to Figure 2.

TABLES

Table S1: Stability studies of IPM-MOF-201, related to Figure 2.
Content of Ni (in ppm) in the supernatant solution recorded using ICP-AES.

**Phase**	**Ni(ppm)**
pH = 4 (2 days)	1.85
1N NaOH (1day)	0.485
NMR Supernatant (1 day)	0.033

Table S2. **Crystal data and structure refinement for IPM-MOF-201, related to Figure 1**.

Identification code	IPM-MOF-201	
Empirical formula	C54 H42 N14 Ni	
Formula weight	945.72	
Temperature	100(2) K	
Wavelength	0.71073 Å	
Crystal system	Trigonal	
Space group	R-3:H	
Unit cell dimensions	a = 16.0448 (10) Å	α= 90°.
	b = 16.0448 (10) Å	β= 90°.
	c = 25.0057 (18) Å	γ = 120°.
Volume	5574.9 (8) $Å^3$	
Z	3	
Density (calculated)	0.845 Mg/m^3	
Absorption coefficient	0.30 mm^{-1}	
F(000)	1476	
Crystal size	0.17 x 0.12 x 0.10 mm^3	
Theta range for data collection	2.2 to 28.4°.	
Index ranges	-19<=h<=21, -21<=k<=21, -33<=l<=33	
Reflections collected	46123	
Independent reflections	3119 [R(int) = 0.131]	
Completeness to theta = 28.42°	0.999	
Absorption correction	Semi-empirical from equivalents	
Refinement method	Full-matrix least-squares on F^2	
Data / restraints / parameters	3119 / 0 / 105	
Goodness-of-fit on F^2	1.164	
Final R indices [I>2sigma(I)]	R1 = 0.090, wR2 = 0.2959	
R indices (all data)	R1 = 0.1542, wR2 = 0.334	
Largest diff. peak and hole	0.43 and -0.37 e.$Å^{-3}$	

Table S3: Stability studies of IPM-MOF-201, related to Figure 2.
Comparison of unit cell parameters of the compound treated under different conditions.

**Cell Parameters**	**As-synthesized**	**Dipped in pH=4**	**Dipped in 1N NaOH**
a	16.0448(10)	15.95±0.05	15.96±0.07
b	16.0448(10)	15.95±0.05	15.96±0.07
c	25.0057(18)	25.12±0.09	25.02±0.012
α=β	90	90	90
γ	120	120	120
Volume (Å^3)	5574.9(8)	5540±30	5520±50

TRANSPARENT METHODS

Materials: All solvents and reagents were commercially available and used without further purification. Standard pH buffers (pH = 4.01, 10.01, 12.45) were procured from Eutech Instruments. The other pH conditions were prepared using concentrated HCl and NaOH flakes.

Synthesis of Ligand {Tris(4-(1H-imidazol-1-yl)phenyl)amine}: The ligand was synthesized according to the reported protocol (Desai et al., 2016). The mixture of tris(4-bromophenyl)amine (500 mg, 1.04 mmol), imidazole (423 mg, 6.22 mmol), K_2CO_3 (573 mg, 4.15 mmol) and $CuSO_4$ (6.5 mg, 0.041 mmol) was kept for heating at 150^oC under inert atmosphere for 60 hours. Upon cooling to room temperature, CH_2Cl_2 (50 ml) was poured into the reaction mixture and washed several times with deionised water. The organic layer was evaporated under reduced pressure yield a pale coloured powder. The obtained ligand was recyrstallized from a mixture of MeOH:CH_2Cl_2 (1:1) in yield of 68%.

Synthesis of Compound IPM-MOF-201: A mixture of ligand (8.86 mg, 0.02 mmol), $NiSO_4 \cdot xH_2O$ (5.60 mg, 0.02 mmol), 4,4'-biphenyldisulfonic acid [H_2BPSA] (9.45 mg, 0.03 mmol), N,N-dimethylformamide (2 ml) and water (1 ml), was placed in a teflon capped pyrex tube, and heated at 130^oC for 48 hours followed by slow cooling to room temperature. The compound was filtered and washed with water and methanol several times. Pale green colour crystals of compound IPM-MOF-201 viz. $[\{Ni(L)_2\}\cdot(BPSA)\cdot xG]_n$ were isolated in ~40% yield. These crystals were dipped in MeOH solution for 2 days prior to heating it under vacuum at 75^oC to obtain the guest free phase. We were unable to locate the highly disordered guest solvent molecules and uncoordinated anion in the structure crystallographically. From the SQUEEZE function of PLATON the formula for the guest-free phase was estimated to be $[\{Ni(L)_2\}\cdot(BPSA)]_n$. Anal. Calcd.: C/N, 4.04; N/S, 3.06; C/S, 12.35. Found: C/N, 3.98; N/S, 3.15; C/S, 12.57.

pH Stability Test: ~50 mg of the activated compound IPM-MOF-201 was dipped in 5 ml of separate pH solution (pH = 4, 10.01, 12.45, 14) and stirred at room temperature for 24 hours. The respective solutions were filtered off (the supernatant was collected and submitted for ICP-AES analyses) and the residue was washed with deionised water multiple times, followed by washing with methanol. All the phases were then degassed by heating under reduced pressure for further characterization. For gas adsorption studies, the compounds were pre-treated at 120^oC under vacuum before measurement.

To validate the base resistance of the compound, 20 mg of the activated phase of **IPM-MOF-201** was added to a solution of ~1N NaOD (0.5 ml, 40 wt%; Sigma Aldrich) in D_2O (4.5 ml; Sigma Aldrich) and kept at room temperature for 24 hours. This mixture was centrifuged and ICP-AES analysis was performed on the supernatant. The residue was then treated with $CDCl_3$ (3.0 ml; Sigma Aldrich) and the NMR spectra for this supernatant was also recorded. The residue obtained after the second step was used for further characterization (Scheme S3).

Dye Exchange Studies: ~10 mg of activated crystalline powder of IPM-MOF-201 was dipped in aqueous solution of methyl orange [MO] (1 mM, 2 ml) for 2 days at room temperature to yield the dye-encapsulated product. After anion exchange the compound was washed with deionised water and methanol several times to remove any dye adsorbed on the surface of the compound. The air-dried compounds were then used for further characterization. The same protocol was followed for other dyes viz. indigo carmine [IC], alizarin red S [ARS], methyl red [MR], 4-phenylazophenol [PAP]. Similarly, the dye exchange experiments were carried out at other pH conditions (pH = 4, 10.01, 12.45, 14). For

the UV-vis measurements, the standard deviation was calculated from five blank measurements of the dye solutions. The standard deviations for MO, IC, ARS, MR, PAP dyes were calculated to be 0.00589, 0.00728, 0.00187, 0.00083, 0.0013 respectively.

Synthesis of analogous Ni-MOFs: Metal salts of Ni^{2+} (NO_3^-, Cl^-, SiF_6^{2-}, Br^-, OTf^-; 0.02 mmol each) were added separately to the mixture of ligand (8.86 mg, 0.02 mmol), 4,4'-biphenyldisulfonic acid [BPSA] (9.45 mg, 0.03 mmol) in N,N'-dimethylformamide (DMF; 2 ml) and water (1 ml). The mixture was heated at 130°C for 48 hours in a Pyrex tube. Upon cooling the compounds were washed with water and methanol and dried in air for further characterization.

Characterization of the free anion: The presence of the free anion ($BPSA^{2-}$) was characterized by digesting the MOF. To minimize the content of the transition metal ion Ni(II) in the NMR sample, a 2-step protocol was employed for validating the presence of the free anion (Scheme S2). To 20 mg of the activated phase of **IPM-MOF-201** in 0.5 ml D_2O, 0.2 ml D_3PO_4 (85% in D_2O) was added. To this solution NaOD (40 wt%) was added dropwise till the pH became neutral. As the pH reached neutral heavy precipitation was observed. This was separated by centrifugation and the supernatant was used to record the ^{1}H-NMR spectrum.

Water Stability Test: 20 mg of activated compound **IPM-MOF-201** was dipped in 5 ml of deionised water and kept at room temperature for 7 days. Subsequently, the compound was filtered off and dried in air for further characterization. For FESEM images, the compound was kept in deionised water for 30 days.

Control Experiment: Compound **IPM-MOF-201(Co)** was synthesized following above protocol, replacing $NiSO_4{\cdot}xH_2O$ with $CoSO_4{\cdot}7H_2O$ (5.62 mg, 0.02 mmol). The crystalline powder thus obtained was filtered off and washed with water and methanol. It was activated under reduced pressure at 75°C and used for further characterization. For checking the pH stability, 10 mg of the crystalline powder was dipped in pH=12.45 buffer and pH=14 solution for 24 hours at room temperature. Subsequently it was washed with deionised water and acetonitrile, and dried in air for further characterization. The Co-MOF & Cd-MOF used for comparison were synthesized according to reported protocols (Yao et al., 2011; Liu et al., 2014).

Dye Content Calculation: The dye content in the solution was calculated using a reported protocol (Song et al., 2015). Time dependent UV-vis absorbance of the supernatant were recorded. The absorbance maxima corresponding to each dye in different pH was chosen to compute the dye content using the formula:

$$D = (A_t/A_o) \times 100\%$$

where D is the dye content in the corresponding pH solution, A_o characteristic absorbance of the solution before addition of the MOF, A_t is the absorbance of the solution at different time intervals.

Blank Dye Solution Experiment: The UV-vis spectra of blank aqueous dye solutions [~0.05mM] (without addition of MOF) was recorded at two time intervals (t=0 and t=24h) to validate the ion-exchange experiments in the presence of MOF.

Recycling Experiment: To an aqueous solution of MO (0.09 mM) ~10 mg of the activated phase of IPM-MOF-201 was added. After keeping it for 24 hours, the supernatant was collected and its UV-vis spectrum was recorded. The filtrate was air-dried and added to an aqueous solution of tetrabutylammonium chloride (50wt%, 2 ml). The supernatant was

collected after 24 hours and its UV-vis spectrum was monitored. The filtrate was air-dried and this cycle was repeated further.

MOF-loaded Column Experiment: A glass tube with narrow opening was used for this experiment which was plugged by thick cotton at the bottom. ~20 mg of the activated phase of IPM-MOF-201 was filled followed by addition of a uniform layer of white sand. This set-up was rinsed with deionised water 2-3 times before further use. A mixture of MO and MB (0.05 mM, 5 ml each) in deionised water was added to this column. The eluted MB solution was collected and then deionised water was purged until no further dye eluted out. Following this tetrabutyl ammonium chloride aqueous solution (50wt%) was passed to elute the trapped MO dye. This was passed until no further MO dye passed out.

Physical Measurements: Powder X-ray diffraction patterns were recorded on Bruker D8 Advanced X-Ray diffractometer using Cu Kα radiation (λ= 1.5406 Å) in 5° to 40° 2θ range with a scan speed of $1.2^{o}min^{-1}$. The IR Spectra were acquired by using NICOLET 6700 FT-IR spectrophotometer using KBr pellet in 400-4000 cm^{-1} range. UV spectra were recorded on Shimadzu UV 2600 Spectrophotometer having stirring attachment. The SEM images & EDX data were obtained using FEI Quanta 3D dual beam ESEM. Thermogravimetric analysis profiles were recorded on Perkin-Elmer STA6000, TGA analyser under N_2 atmosphere with heating rate of $10^{o}C$/min. Gas adsorption measurements were performed using BelSorp-Max instrument (Bel Japan). Solvent adsorption measurements were performed on Bel-Aqua instrument (Bel Japan). Prior to adsorption measurements, the activated samples were heated at $130^{o}C$ under vacuum for 6 hours using BelPrepvacII. ^{1}H & ^{13}C NMR spectra were recorded on a JEOL 400 MHz or Bruker 400 MHz spectrometer.

X-ray Structural Studies: Single-crystal X-ray data of compound IPM-MOF-201 was collected at 100 K on a Bruker D8 Venture Duo X-ray diffractometer equipped with Microfocus X-ray source (operated at 50W; 50kV/1mA), graded multilayer optics for monochromatic Mo Kα radiation (λ = 0.71073Å) focused X-ray beam and Photon 100 CMOS chip based detector system. Crystal was mounted on nylon CryoLoops (Hampton Research) with Paraton-N (Hampton Research). The data integration and reduction were processed with SAINT (*SAINT Plus*, 2004) software. A multi-scan absorption correction was applied to the collected reflections (Krause et al., 2015). The structure was solved by the direct method using SHELXTL (Sheldrick, 2008) and was refined on F^2 by full-matrix least-squares technique using the SHELXL-2014/7 (Sheldrick, 2015) program package within the WINGX(Farrugia, 2009) programme. All non-hydrogen atoms were refined anisotropically. All hydrogen atoms were located in successive difference Fourier maps and they were treated as riding atoms using SHELXL default parameters. The structures were examined using the *Adsym* subroutine of PLATON to assure that no additional symmetry could be applied to the models. High disorder was observed for the guest solvent molecules and the anion (BPSA). The SQUEEZE option (Spek, 2015) was used to eliminate the contribution of disordered guest molecules and anion. The number of the anions required to maintain the electrical neutrality was estimated and the molecular formula was calculated accordingly.

SUPPLEMENTAL REFERENCES

SAINT Plus, (2004). version 7.03; Bruker AXS Inc.: Madison, WI.

Krause, L., Herbst-Irmer, R., Sheldrick, G. M., and Stalke, D. (2015). Comparison of silver and molybdenum microfocus X-ray sources for single-crystal structure determination. J. Appl. Cryst. *48*, 3-10.

Sheldrick, G. M. (2008). A short history of *SHELX*. Acta. Cryst. *A64*, 112-122.

Sheldrick, G. M. (2015). Crystal structure refinement with *SHELXL*. Acta. Cryst. *C71*, 3-8.

Farrugia, L.(2009). *WinGX*, version 1.80.05; University of Glasgow: Glasgow, Scotland.

Spek, A. L. (2015). *PLATON* SQUEEZE: a tool for the calculation of the disordered solvent contribution to the calculated structure factors. Acta. Cryst. *C71*, 9-18.
